# *In silico* construction of a multi-epitope vaccine (RGME-VAC/ATS-1) against the *Rickettsia* genus using immunoinformatics

**DOI:** 10.1590/0074-02760240201

**Published:** 2025-03-21

**Authors:** Andrei Giacchetto Felice, Thaís Cristina Vilela Rodrigues, Pedro Henrique Marques, Felipe Lucas Zen, Marcela Rezende Lemes, Rafael Obata Trevisan, Bruno Silva Andrade, Carlo José Freire de Oliveira, Vasco Ariston de Carvalho Azevedo, Sandeep Tiwari, Siomar de Castro Soares

**Affiliations:** 1 Universidade Federal do Triângulo Mineiro, Instituto de Ciências Biológicas e Naturais, Programa de Pós-Graduação em Medicina Tropical e Infectologia, Uberaba, MG, Brasil; 2 Universidade Federal de Minas Gerais, Departamento de Genética, Ecologia e Evolução, Belo Horizonte, MG, Brasil; 3 Universidade Federal de Minas Gerais, Instituto de Ciências Biológicas, Programa de Pós-Graduação em Bioinformática, Belo Horizonte, MG, Brasil; 4 Universidade Estadual do Sudoeste da Bahia, Departamento de Ciências Biológicas, Vitória da Conquista, BA, Brasil; 5 Universidade Federal do Triângulo Mineiro, Instituto de Ciências Biológicas e Naturais, Departamento de Microbiologia, Imunologia e Parasitologia, Uberaba, MG, Brasil; 6 Universidade Federal da Bahia, Instituto de Biologia, Programa de Pós-Graduação em Microbiologia, Salvador, BA, Brasil; 7 Universidade Federal da Bahia, Instituto de Ciências da Saúde, Programa de Pós-Graduação em Imunologia, Salvador, BA, Brasil

**Keywords:** Rickettsia, proteins, bioinformatics, vaccines

## Abstract

**BACKGROUND:**

*Rickettsia* is a genus of Gram-negative bacteria that causes various diseases, including epidemic typhus, Rocky Mountain spotted fever, and Mediterranean spotted fever. Ticks transmit these diseases and commonly found in developing regions with poor sanitation. As a result, it is difficult to estimate the number of these diseases cases, making it challenging to create prevention and diagnostic mechanisms.

**OBJECTIVES:**

Thus, this study aimed to develop an *in silico* multi-epitope vaccine against *Rickettsia*.

**METHODS:**

Eight proteins were previously identified as potential vaccine candidates through reverse vaccinology and were screened for epitopes that bind to MHC class I and II molecules. The epitopes were then analysed for antigenicity, allergenicity, and toxicity. The selected epitopes were linked with AAY and GPGPG sequences peptide and a known adjuvant, the B-chain of *Escherichia coli* heat-labile enterotoxin, to form a chimeric multi-epitope protein. The protein’s three-dimensional structure was predicted, and molecular docking analysis was performed against the toll-like receptor 4 (TLR4). Finally, the immune response to the protein was simulated using C-ImmSim tool.

**FINDINGS:**

A total of 26 immunogenic epitopes, formed the multi-epitope vaccine RGME-VAC/ATS-1. The vaccine showed excellent immunogenic parameters and was predicted to do not be toxic or allergenic to the host. It also showed good potential stimulation of immune cells, with a propensity to generate memory cells and elicit IFN-γ secretion.

**MAIN CONCLUSIONS:**

The *in silico* validations suggest that our study successfully designed an innovative multi-epitope vaccine against *Rickettsia*, addressing the challenges posed by the elusive nature of diseases caused by this genus. We provide a promising potential for further experimental exploration and the development of targeted prevention and diagnostic strategies for these diseases.

The genus *Rickettsia* comprises obligatory intracellular Gram-negative bacteria that primarily target endothelial cells.^1^ ^,^ ^2^ Rickettsiae are considered emerging pathogens and inhabit a wide range of arthropod vectors, such as fleas, ticks, mites, and lice.^3^ Traditionally, rickettsiae are categorised into two main groups for causing harmful human diseases. The first group encompasses species responsible for typhus (TG), and the second group, the spotted fever group (SFG), predominantly includes species causing spotted fever. There is also a two other groups, the transitional group that exhibits characteristics of TG and SFG groups and the ancestral group, nowadays believed to be ancestral species for the other groups. Additionally, health-disease relationships between these microorganisms belonging to these last two groups and humans have not yet been discovered.^1^ ^,^ ^4^ ^,^ ^5^ ^,^ ^6^ ^,^ ^7^

Previously it was suggested that these bacteria undergo vertical transmission processes in vectors, aiding in maintaining natural infection. However, some must complete their life cycle in multiple hosts to ensure survival.^8^ *Rickettsia* transmission generally occurs through salivary products or inoculation of infected vector faeces into wounds. After the bacteria are phagocytosed and replicate in lymph nodes, they disseminate through the bloodstream to endothelial cells.^9^ ^,^ ^10^ This infection results in symptoms such as nausea, vomiting, eschars at the tick bite site, maculopapular rash, fever, and multiple organ failure in more severe cases.^3^ ^,^ ^4^ ^,^ ^11^

*Rickettsia* has a global distribution based on the prevalence of their vectors in each region. Despite being well-known organisms, rickettsioses are neglected because they are more prevalent in developing countries, further complicating the assessment of the number of cases of these diseases.^12^ TG group species transmitted by lice and fleas have a tropism for tropical and subtropical regions and unhygienic conditions.^13^ ^,^ ^14^ ^,^ ^15^ ^,^ ^16^ On the other hand, the SFG group is primarily transmitted by ticks and can be found throughout the Americas and in regions such as Europe, Africa, and Asia.^3^ ^,^ ^17^ ^,^ ^18^

As mentioned above, diagnosis and treatment also become challenging due to their presence in developing regions and being considered neglected diseases. Recognising clinical symptoms and having knowledge of local epidemiology and travel history to endemic areas are crucial for differential diagnosis from other febrile illnesses. This diagnosis can be achieved through bacterial culture, polymerase chain reaction (PCR), and indirect immunofluorescence (IFA).^4^ ^,^ ^19^ ^,^ ^20^ ^,^ ^21^ Despite the availability of these diagnostic methods, not all laboratories have access to them, further complicating rickettsia identification. Since diagnosis is often difficult and time-consuming, treatment may be hindered. Some drug treatments have shown *in vitro* efficacy, such as doxycycline (tetracyclines) against the overall group, and some variations among them, such as amoxicillin (beta-lactam) and gentamicin (aminoglycoside). When not initiated immediately, the disease prognosis becomes poor.^22^ ^,^ ^23^

The first attempts at immunisation began shortly after the First World War, isolating whole cells of *Rickettsia prowazekii* and/or *Rickettsia rickettsia*.^24^ Tested on guinea pigs, both species were also used in the Second World War by the German army, as an attempt to survive the unsanitary conditions that possibly contained disease transmissions.^25^ ^,^ ^26^ Other isolated test attempts with *R. rickettsii* inactivated by formalin and developed in chicken fibroblasts were used, the *in vitro* tests produced interesting results in monkeys, but did not prevent it in humans.^27^ *Orientia tsutsugamushi* was also isolated from rat lungs for further tests on C3h/HeN mice in the 1940s, and despite being a species closely related to *Rickettsia* and even considered to be in the same genus in the past, it unfortunately did not produce satisfactory results in humans.^25^ The difficulty of studying the virulence factors of this bacterium during the last century led to promising tests on animals, but not on humans, who were still infected with these bacteria.^28^ ^,^ ^29^ As a result, tests were restricted to these strains and no vaccine was developed that had reliable data and even today, they are no longer used and there is currently no vaccine.^24^

Currently, there are no means of prevention against the bacteria as a whole, only measures against their vectors.^30^ This can be explained by the fact that *Rickettsia* species are obligate intracellular organisms, which complicates the entire process of cultivation and isolation of the pathogen, in addition to the need for high-security laboratories to handle them. When all the difficulties have been overcome, obligate intracellular microorganisms have the capacity to produce a Th1-type immune response, which has great potential against these types of microorganisms. This type of response preferentially is responsible for stimulating a potential differentiation of CD4+ lymphocytes into producers of cytokines such as IFN-γ, an important molecule in the regulation and survival of T cells, as well as helping to stimulate the production of antibodies and immunoglobulins by B lymphocytes. It also has a differentiation pattern of CD8+ lymphocytes for the production of cytokines such as IFN-γ, TNF and LT-α, also causing stimulation of B cells.^31^ ^,^ ^32^

Fields reverse vaccinology and immunoinformatic have shown promising strategies for predicting potential vaccines *in silico*. The reverse vaccinology (RV) makes it possible to search for proteins with great potential to connect with important cells of the immune system, such as the histocompatibility complex, thus facilitating a possible stimulation of the immune response.^33^ ^,^ ^34^ ^,^ ^35^ Using bioinformatics tools, multi-epitope vaccines can be developed by combining epitopes of the proteins suggested by RV, with high potential to elicit immune responses whether cellular or humoral. These vaccines are created using genomic sequences conserved among all microorganism strains. They are tested for non-homology to the host genome, helping to avoid possible adverse reactions, often observed in inactivated or attenuated vaccines.^36^ ^,^ ^37^ ^,^ ^38^ Moreover, some multi-epitope vaccines have already been developed with the similar methodologies against bacteria such as *Staphylococcus aureus*,^39^ *Mycoplasma pneumoniae*,^40^ *Mycobacterium leprae*,^41^ and even against viruses causing influenza,^42^ yellow fever,^43^ and have been considered potential *in silico* effective candidate vaccines. Multi-epitope vaccines have also been tested in *in vivo* models and have also shown promising results against *Helicobacter pylori* ^44^ and chronic lymphocytic choriomeningitis virus (LCMV)^45^ infections.

Therefore, developing new prevention strategies against *Rickettsia* becomes necessary, and *in silico* approaches emerge as an efficient and agile alternative for searching for new vaccine targets against rickettsioses.

## MATERIALS AND METHODS

No unexpected or unusually high safety hazards were encountered

*Selection of antigenic proteins* - A previous study utilising 47 complete genomes of the *Rickettsia* genus identified, through reverse vaccinology, eight proteins belonging to the core genome with high binding affinity to MHCI/MHCII molecules.^33^ It is known that proteins belonging to the core genome have a high level of expression and, consequently, a high potential to be considered immunogenic.^46^ Therefore, those eight proteins [Supplementary data (Table I)] were selected for constructing the multi-epitope vaccine. For the next tools they were submitted in FASTA format for epitope prediction and then they were submitted in primary sequences in the next analyses.

Epitope prediction

*MHCI/CTL binding epitopes* - Two online software tools were used to predict epitopes of the selected proteins that binds the major histocompatibility complex (MHC), molecules that are presented to cytotoxic T lymphocytes (CTL) to induce an immune response, improving the confidence of the analysis: The Immune Epitope Database (IEDB)^47^ and the NetCTL 1.2 server.^48^ IEDB is a database with various epitopes already classified as highly or lowly immunogenic and has a specific tool for predicting MHCI-binding epitopes. To achieve maximum population coverage in this prediction, we used the 27 most prevalent MHCI human alleles worldwide with a length of nine amino acids, excluding from the analysis all with size 10 [Supplementary data (Table II)], described by Greenbaum et al.;^49^ The NetMHCpan 4.1 EL prediction method was used and the result with the epitopes sorted in a decreasing way by prediction score and exported in XHTML table format. NetCTL was used to screening the epitopes regarding the stages of the antigen presentation process, including proteasomal C-terminal cleavage, efficient transport, and strong MHCI binding affinity. For the NetCTL analysis, each of the proteins was submitted in FASTA format and then the allelic supertypes that most closely matched the alleles used in the IEDB’s MHCI binding tool were chosen [Supplementary data (Table II)]. For the rest of the analyses, the default parameters were maintained, with 0.15 for weight on C terminal cleavage, 0.05 for weight on TAP transport efficiency and 0.75 for threshold for epitope identification, and the results classified using the combined score option.

*MHCII/HTL binding epitopes* - To predict epitopes that bind to MHC II and interact with helper T lymphocytes (HTL), two software tools were also used: the MHC II epitope prediction tool from IEDB^47^ and the NetMHCII 2.3 server.^50^ ^,^ ^51^ MHC II binding grooves are slightly larger than those of MHCI, accommodating epitopes ranging from 13 to 25 amino acids in length. Therefore, we selected 15 amino acid length sequences, which is considered the standard size. The IEDB’s MHC II epitope binding tool was used with its default parameters, choosing all the alleles available for human (HLA-DR) selecting the option “Select full HLA reference set” [Supplementary data (Table II)], classifying the epitopes by percentile rank and exporting the result in XHTML table. For NetMHCII web server, each protein was submitted in FASTA format, after this, a peptide length of 15 was set in our analysis. Then, we used the loci HLA-DR, HLA-DP and HLA-DQ, selecting the same alleles tested in web server IEDB MHCII binding epitope [Supplementary data (Table II)]. The values for other options were used in default mode, being -99.9 for threshold, two for threshold for strong binder (% rank) and 10 for threshold for weak binder (% rank). In addition, the result was classified/sorted by affinity.

*B-cell binding epitopes* - The ABCpred tool utilised artificial neural networks (ANN) and support vector machines (SVM) approaches and was used to predict linear B-cell epitopes. A 16-residue epitope size was selected, with a threshold of 0.51, along with standard parameters for improved prediction.^52^ ^,^ ^53^ ^,^ ^54^

*Filtering of the best epitopes* - Once predicted, we first applied filters to reduce the number of epitopes, selecting only the best affinity. For the MHCI epitopes predicted by IEDB, we first set those with a percentile rank < 1. Then, we use Microsoft Excel (2019) tool to eliminate duplicated epitopes. For the MHCI epitopes predicted by NetCTL, we used those with a quality rating of “<- E” by the program itself and then used the duplicate removal tool. For the MHCII epitopes predicted by the IEDB tool, we initially separated the epitopes with a percentile rank < 10. Since there were still many epitopes, we performed an additional filtration using the Toxinpred web service^55^ to exclude predicted toxic epitopes. Subsequently, we used the duplicate removal tool and then selected epitopes with an IC50 < 500 for further analysis. Finally, for the MHCII epitopes predicted by the NetMHCII tool, we decided on those with a quality filter of “SB” (strong binder) by the program itself and then used the duplicate removal tool. All epitopes predicted by ABCpred were used. After filtering, an *in-house* script was used to overlap the results of the two programs, first for the filtered MHCI predicted epitopes, from IEDB and NetCTL, and then the development of this overlap with B-cell epitopes. Following this result, the Class I immunogenicity tool^56^ from IEDB was used to predict the immunogenicity of the remaining MHCI epitopes. Thus, the remaining epitopes underwent to another curation, manually at this time, to remove epitopes with up to two differences in amino acid composition, in order to reduce the epitope quantity further. Finally, these epitopes were submitted to the Vaxijen tool,^57^ with a threshold of 0.7, to select the most immunogenic epitopes. For the filtered MHCII epitopes, the same procedure was carried out: an *in-house* script was used to overlap the results of IEDB vs NetMHCII and then the result vs B-cell epitopes. As mentioned before, MHCII epitopes underwent removal were also selected by removing the very similar ones and then, subsequently submitted to the Vaxijen tool with the same threshold of 0.7.

*Construction of the multi-epitope protein* - The final epitopes that passed through all the filtering criteria were merged to create the sequence of the multi-epitope protein. For this process, another *in-house* script was used, which generated 1000 random models with permutations of the epitopes in different positions and, subsequently, submit them to Vaxijen where only the most antigenic sequences are selected. This sequence was then reinserted into the same script, which added the peptide ligands AAY and GPGPG between CTL/B epitopes and only GPGPG between HTL/B epitopes,^36^ where the ligands are essential for various functions such as protein stabilisation and antigen presentation, for example.^58^ Additionally, the precursor sequence of the B-chain of *Escherichia coli* heat-labile enterotoxin (GenBank Accession: ALO79813.1), a known adjuvant was added,^59^ followed by the peptide sequence EAAAK^60^ to, to enhance the immunogenic activity of the multi-epitope line. In addition to stimulating an increase in dendritic cells and their ability to present antigens to T cells, although not yet tested for Rickettsiae, this adjuvant was chosen because it has been used in research with *O. tsutsugamushi*, which has already been considered to belong to the *Rickettsia* genus due to its similarities, and showed promising results in stimulating an immune response and the subsequent acquired immunity when used.^61^ ^,^ ^62^

*Prediction of secondary, tertiary, and conformational B-cell epitope structures* - The PSIPRED WebService^63^ was used to predict the secondary structure of the protein based on neural networks and the position-specific scoring matrix (PSSM). For the tertiary structure, the ColabFold tool^64^ was used, an online predictor that relies on a rapid homology search with other programs like MMseqs2 with AlphaFold2 or RoseTTAFold, hosted at https://colab.research.google.com/github/sokrypton/ColabFold/blob/main/AlphaFold2.ipynb. The best structure generated by ColabFold was submitted to the GalaxyRefine tool,^65^ which refines the protein structure by adjusting side-chain regions to allowed regions. Finally, the PROCHECK tool^66^ through the SAVES v6.0 WebService generated a Ramachandran plot to assess the quality of the structure. In addition to the linear epitopes already mentioned, conformational B-cell epitopes are also regions of amino acids essential for influencing the host’s immune response. The ElliPro server^67^ was used to predict such epitopes with its default parameters.

*Evaluation of the multi-epitope protein and interaction with the host* - RGME-VAC/ATS-1 was subjected to various programs to validate the multi-epitope sequence, including Vaxijen to determine its antigenicity and the Vaxign web service^68^ to confirm its binding ability to MHCI/II. The Allertop v2.0 tool^69^ was used for allergenicity analysis, and the ToxinPred tool was used for toxicity assessment. The ProtParam tool^70^ also was used to analyse the amino acid composition, the isoelectric point (pI) of the protein, the aliphatic index, the molecular weight, the half-life coefficient, and the grand average of hydropathicity (GRAVY). The Protein-Sol tool^71^ applied *E. coli* data to assess the solubility index. Lastly, the sequence was subjected to BLAST analysis to search homology with host proteins.^72^ All programs were used with their default parameters.

*Molecular docking and molecular dynamics in protein-receptor interaction* - The toll-like receptor type 4 (TLR-4) was chosen as the receptor for this analysis due to its importance for the *Rickettsia* genus.^73^ ^,^ ^74^ ^,^ ^75^ After this selection, the TLR-4 receptor structure was retrieved from the PDB database (PDB ID: 3FXI).^76^ With Chimera software^77^ we removed water molecules, ligands, and other side chains. Subsequently, molecular docking was performed between the vaccine and the TLR-4 structure using the Cluspro v2.0 program.^78^ This program divides the analysis into three steps: rigid anchoring in billions of conformations, root mean square deviation (RMSD) in clusters of 1000 structures representing the most likely conformations, and refinement of systems based on energy minimisation of the complex. The LigPlot+ program was used to visualise the amino acid residues in the protein-receptor interaction. Molecular dynamics analysis was carried out using the Gromacs v 2023.1 program.^79^ This program analyses macromolecule vibrations and projects interaction dynamics between the protein and the TLR-4 receptor structures. The system simulation was performed with the CHARMM36 all-atom force field.^80^ The protein model was solvated with SPC/E 216 water model in a cubic box with a 1.8 nm distance from the edge of the complex and, NaCl ions were added to neutralise the system. In addition, the energy minimisation, equilibration phase with temperature around 300k and pressure around 1 bar were performed to evaluate the equilibration of the system. Finally, the production of the molecular dynamics was performed in 100 nano seconds (ns), among RGME-VAC/ATS-1 and TLR-4 receptor and only TLR-4 alone, with the intention to observe if the complex between the protein and TLR4 receptor can be considered more stable that only TLR-4 alone.

*In silico cloning* - *In silico* cloning analysis was conducted to assess the potential for our multi-epitope construct to be cloned and expressed in a vector. To achieve this, a tool called Jcat^81^ was employed for reverse translation and codon adaptation. The *E. coli* K12 expression system was utilised with the cDNA sequence of the protein. Codon adaptation was assessed based on the CAI index, which should exceed 0.8, and an average GC content ranging from 30 to 70%. The output of this program is referred to as the insert. The insert was virtually inserted into an *E. coli* pET28a(+) vector with EagI and BamHI restriction enzymes introduced at the beginning and end of the sequence, respectively. Both enzymes were not naturally present in our construct after codon adaptation and exhibited similar experimental characteristics; therefore, they were chosen. This procedure was performed using SnapGene^82^ with its default parameters.

*Assessment of potential host immune response* - The ability of the epitopes to induce IFN-γ was predicted using the IFNEpitope server;^83^ to induce IL-6 was predicted by IL6pred, with a threshold 0.11;^84^ and TNF-α was made by TNFepitope, with a threshold 0.45.^85^ Finally, to describe the immune response generated by the created multi-epitope vaccine, the C-ImmSim^86^ server performed immune stimulations. Data generated by this program strongly represent the actual and robust development of immunity in humans.^86^ Three injections were simulated, each with a four-week interval,^87^ ^,^ ^88^ ^,^ ^89^ and each was tested with the adjuvant and the adjuvant sequence alone to compare efficacy of our vaccine.

*Computing capacity* - Initial analyses such as antigenic protein selection, epitope prediction, filtering, protein construction, protein structure prediction, vaccine quality assessment, interaction with the host, molecular docking and *in silico* cloning were mostly done using programs available on websites, and were then carried out on an Acer Nitro 5 computer, Intel Core i5 9th Gen, 8 Threads, 24 GB RAM, 1 TB SSD, Nvidia GTX 1650 GPU and with Linux Ubuntu 23.04 LTS as the operational system. The molecular dynamics analysis was carried out on a Supermicro Server, Intel Xeon E5-2680, 40 threads, 128 GB RAM, 15 TB SSD, Nvidia RTX 4060 GPU and with Linux Ubuntu 23.04 LTS as the operational system.

## RESULTS

*The best predicted epitopes for MHCI/CTL, MHCII/HTL, and B-cells* - For MHCI/CTL binding, 55,728 epitopes were predicted by the IEDB tool, and 2,064 epitopes were predicted by the NetCTL tool when considering all eight proteins. Following the same reasoning, for MHCII/HTL binding, 53,116 epitopes were predicted for the eight proteins by the IEDB tool, and 3,358 epitopes were predicted by the NetMHCII tool. Finally, a total of 194 epitopes binding to B-cells were expected by the ABCpred tool. After applying the filtering criteria, the epitopes were overlapped, resulting in 13 epitopes for the CTL/B complex and 13 for the HTL/B complex, which were used for further analysis (Table). A more detailed description of the quantity of epitopes filtered at each stage can be found in the Supplementary data (Table III). No epitopes from the WP_012149185.1 protein met the minimum threshold of 0.7 for Vaxijen, so for our vaccine, it wasn’t used. However, it showed good binding to immune system cells, and future work may consider including it for *in vitro* and *in vivo* tests.

**TABLE:** List the final epitopes selected for constructing the multi-epitope protein

Proteins (ID)	Epitope CTL/B	Immunogenicity Vaxijen (threshold > 0,7)	Epitope HTL/B	Immunogenicity Vaxijen (threshold > 0,7)
WP_012148219.1	KTTRGTVGL	1,21	IHTKMDRTKGVEVLN	0,8
	QVTAGTGGW	1	EYKIMPGLLPYAEIS	0,76
	RVNFFTPKM	0,8	NSDTSIKLEGFYLLE	0,82
	TANTGGNRY	2,1	-	-
WP_012148227.1	TGQFHIAPY	0,9	PKLEYKPNLVGNKTV	1,2
	GQIGVDFYA	0,85	-	-
WP_012148552.1	SSFKITFFF	2,1	GMHIMLYDLKGPLNV	0,97
	-	-	SKMVKVDYPFLIADN	0,98
WP_012148811.1	NECGEVNPI	1,2	-	-
WP_012148819.1	PYIFIATFF	1,7	MALKRDYPKYYPLFS	0,7
	FLLPYIFIA	2,7	-	-
WP_012149174.1	KSTRGNIVK	1,1	MENQWYLKLNAGTMI	0,9
	GTVINVHLK	1,6	NANGITLTVNNPSLT	1,3
	SSMENQWYL	0,8	KNKINFAYQLSLGTS	0,8
	-	-	YQLSLGTSFEVAQGV	0,8
WP_012149175.1	-	-	RTNISYKLTLGTSAQ	1
	-	-	ATSVKLKSNMTVSVD	1,3

ID: identity; CTL: cytotoxic T lymphocytes; HTL: helper T lymphocytes.

**Fig. 1:**
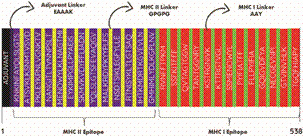
the image represents all selected epitopes interspersed with their respective linkers. Following the adjuvant (black), EAAAK adjuvant linker (dark blue), the epitopes of the helper t lymphocyte (HTL/B) complex (purple) interspersed with their GPGPG linker (yellow) are shown, followed by the epitopes of the cytotoxic T lymphocyte (CTL/B) complex (red) incorporated with their AAY ligands (green). The final structure comprises 538 amino acids.

*Twenty-six epitopes were used to design the multi-epitope vaccine RGME-VAC/ATS-1* - The multi-epitope protein was built with 13 epitopes from the HTL/B complex interspersed with GPGPG peptide linkers and 13 epitopes from the CTL/B complex interwoven with AAY peptide linkers. The adjuvant heat-labile enterotoxin of *E. coli* was added at the beginning of the sequence, followed by an EAAAK peptide linker. Thus, including the adjuvant, the three types of added linkers, and the 26 selected epitopes from the HTL/B and CTL/B complexes, the total length of the multi-epitope protein was 538 amino acids. The final sequence of the protein is depicted in Fig. 1.

**Fig. 2:**
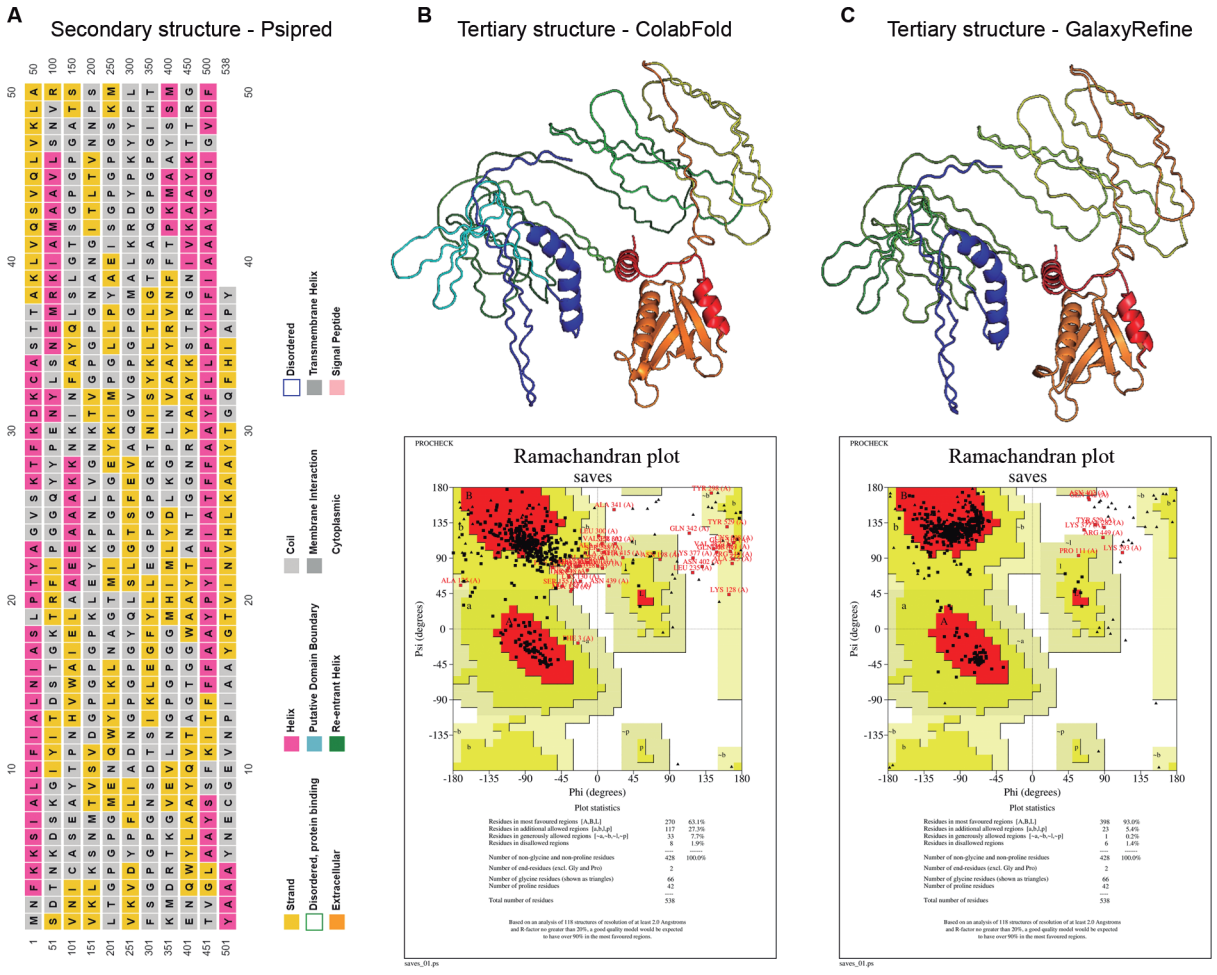
results from the Psipred, ColabFold, GalaxyRefine, and SAVES programs for protein structures. In (A), the secondary structure predicted by Psipred is shown with an explanatory legend of the colour coding for each amino acid in the sequence. In (B), the top portion shows the 3D structure predicted by the ColabFold program and, below, the Ramachandran plot generated by the SAVES web service, with 63.1% of amino acids in favourable regions. In (C), the top portion shows the 3D structure of the protein refined by GalaxyRefine and, below, the Ramachandran plot generated by the SAVES web service, with 93% of amino acids in favourable regions.

*The 3D structure of the protein showed over 90% of residues in favoured regions* - The entire structure of the protein (538 AA) was subjected to the Psipred program. According to the program, the protein structure exhibited 49.64% loops, 30.29% β sheets, and 20.07% helices (Fig. 2A). The best 3D structure predicted by ColabFold showed 63.1% of amino acids in favoured regions, 27.3% in additional allowed regions, 7.7% in generously allowed regions, and 1.9% in the disallowed areas, according to the Ramachandran plot in Fig. 2B. The 3D structure refined by GalaxyRefine increased to 93% of amino acids in favoured regions, 5.4% in additional allowed regions, 0.2% in generously allowed regions, and 1.4% in disallowed regions, as per the Ramachandran plot in Fig. 2C. The Ellipro program predicted 10 discontinuous B-cell epitopes for the multi-epitope protein [Supplementary data (Table IV)].

*The analysis of the chimeric vaccine RGME-VAC/ATS-1 exhibited high quality parameters* - The Vaxijen software considered the designed vaccine as antigenic, with a score of 0.9788. Allertop found no protein epitopes considered allergenic, and Toxinpred did not find any toxic epitopes for humans. These findings indicate significant immunogenic potential and safety for the multi-epitope protein. The protein’s molecular mass was calculated as 57,135.45 (57.14 kDa) with a theoretical pI 9.36. It also showed an instability index of 18.89, associated with an aliphatic index of stability measured at 76.62, which, according to Protparam program parameters, suggests stability. This program also calculated the protein’s half-life: 30 h for mammalian reticulocytes *in vitro*, > 20 h for yeast *in vivo*, and > 10 h for *E. coli in vivo*. The protein exhibited an average sequence hydrophobicity (GRAVY) of -0.089. Finally, according to the Protein-Sol tool, the protein’s solubility was 0.427 (soluble vaccine), and in the BLAST analysis against the human proteome (Taxid: 9606), no significant homology was found.

**Fig. 3:**
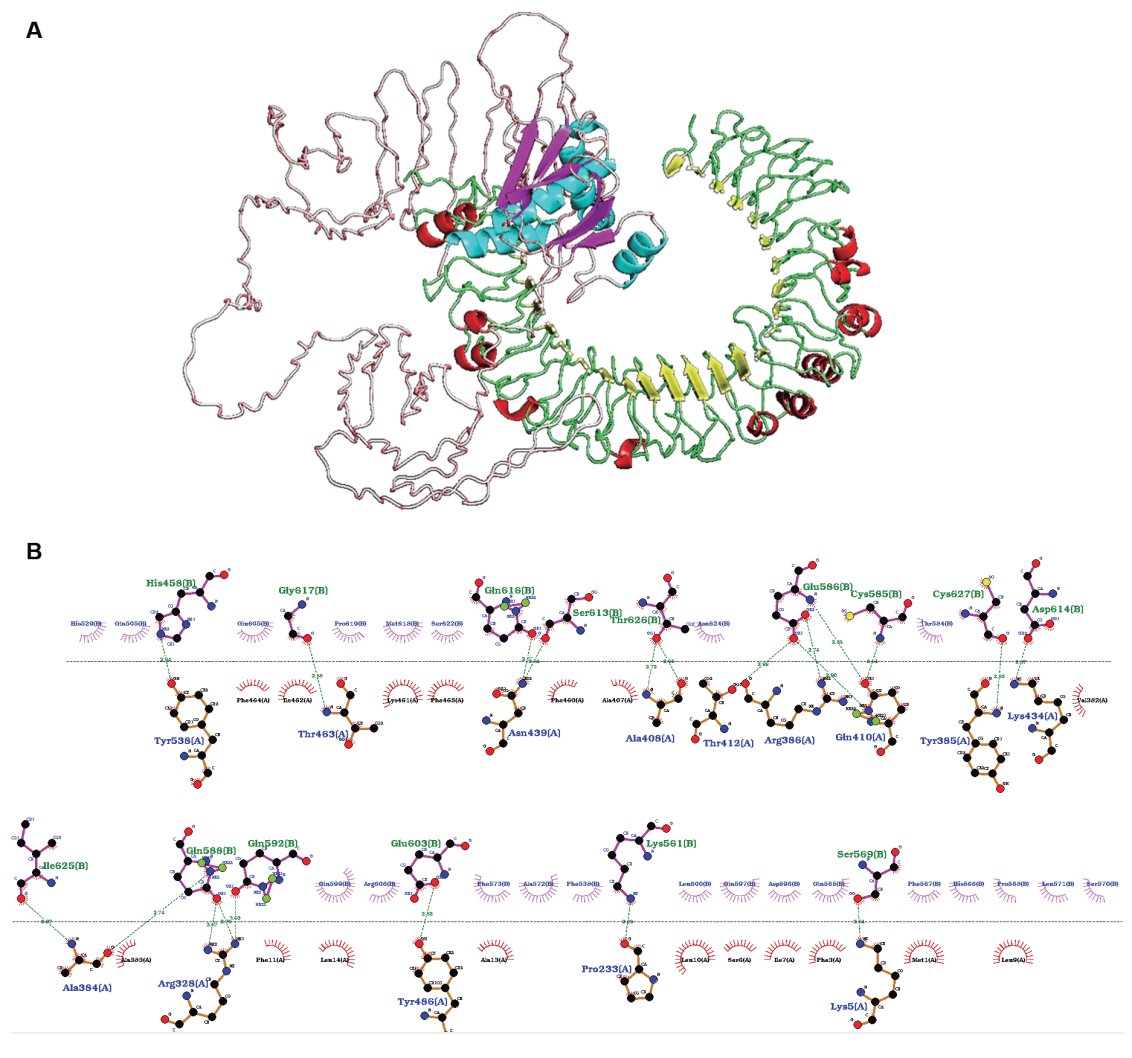
molecular docking technique applied to the multi-epitope protein and toll-like receptor 4 (TLR4). In (A), the interaction generated by the ClusPro program is observed between the protein (on the left, predominantly light gray) and the TLR4 (on the right, mainly green). In (B), the visualisation of the interaction of residues participating in the molecular binding generated with the LigPlot+ program. In this case, hydrogen bonds are represented in green, semicircles below the dashed line in red represent hydrophobic interactions.

**Fig. 4:**
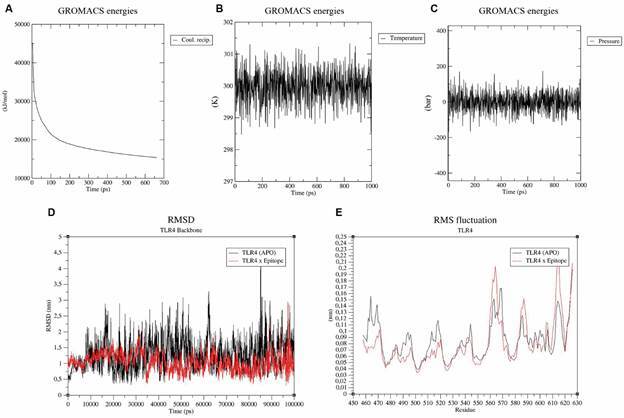
representative graphs of the molecular dynamics of the complex performed with the Gromacs program. The graph A evaluates the energy minimisation of the system, which was around 15 thousand kj/mol in around 700 ps. The graph B demonstrates that the system balanced at around 300k temperature in 10 ns. C graph demonstrates that the system balanced at around 1 bar pressure in 10 ns. The graph D represents the root mean square deviation (RMSD) of the complex (in red line) with a variation among 0,5 nm to 3 nm from the initial position) and the only TLR-4 alone (in black line) with a variation among 0,5 nm to 4 nm from the initial position). The graph E demonstrates the root mean square fluctuation (RMSF) of the interaction region complex (in red line) and the interaction region of TLR-4 alone (in black line) with a variation among 0.04 nm to 0.21 nm from the initial position.

*The RGME-VAC/ATS-1 shows high potential for interaction with TLR4 receptor* - Molecular docking analysis showed that our multi-epitope vaccine exhibited a strong binding affinity with TLR4 receptor, featuring 21 hydrogen bonds and 17 hydrophobic interactions among residues with 22 corresponding atoms (Fig. 3B). According to the ClusPro program, Model 0 ranked best for protein-receptor binding, presenting a cluster with 53 members and a weighted energy score of -1,132.7 (Fig. 3A). The protein-TLR4 complex was used for molecular dynamics simulation of the interaction using the Gromacs program. First, it was performed a minimisation phase to ensure the protein had an appropriate geometry and could have its energy stabilised, which proved to be possible, with a final and stabilised energy of approximately 15,000 kj/mol and which was stabilised at around 700 ps (Fig. 4A). After this process, the system was tested for an equilibrium phase, where within 1000 ps they underwent a process of stimulation of the residues and even with all the stimulation, the system remained stable at around 300 k temperature (Fig. 4B) and around 1 bar pressure (Fig. 4C). This phase helps to check the stability of the complex to be tested for molecular dynamics. Finally, the final dynamics production process was carried out at 100,000 ps (100 ns), generating RMSD, root mean square fluctuation (RMSF) results for the complex (in red line) and only TLR-4 alone (in black line), which are shown in Fig. 4D-E, respectively. For the RMSD, the interaction region of the complex showed greater stability than the interaction region of TLR-4 alone, with its representative line having a smaller variation in position (among 0,5 nm to 3 nm from the initial position) than the line representing the receptor (among 0,5 nm to 4 nm from the initial position). For the RMSF test, the amino acid residues present in the interaction region of the complex also had a smaller fluctuation in almost all the regions shown in the graph compared to the interaction region of TLR-4 alone, and both regions had fluctuations ranging from 0.04 nm to 0.21 nm from the initial position.

**Fig. 5:**
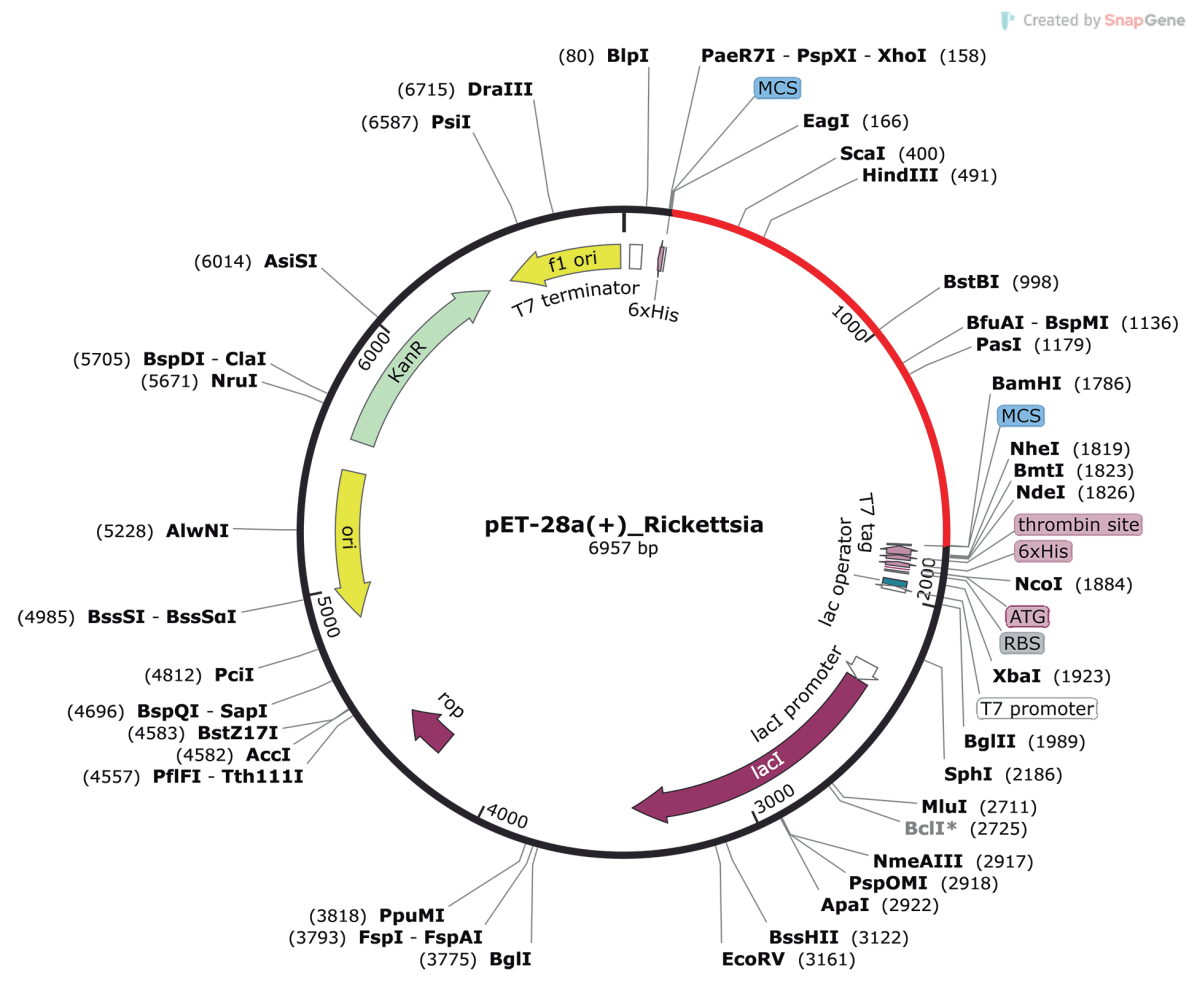
*in silico* cloning of the multi-epitope construct. The optimised construct is depicted in red and inserted into the *Escherichia coli* pET28a(+) vector (in black) through the EagI and BamHI restriction enzymes.

*Expression and cloning in a vector* - The codon adaptation analysis conducted the Jcat program revealed that our construct had a GC content of 51.67% and a CAI index of 1.0, falling within the allowed range. The EagI and BamHI restriction enzymes were employed to insert the construct into the pET28a(+) expression vector. Surrounding the circular image are representations of all the restriction enzymes capable of cleaving the vector when connected to the black part of the circle and those capable of cleaving the insert when connected to the red part. The final clone measured 6957 bp (Fig. 5).

**Fig. 6:**
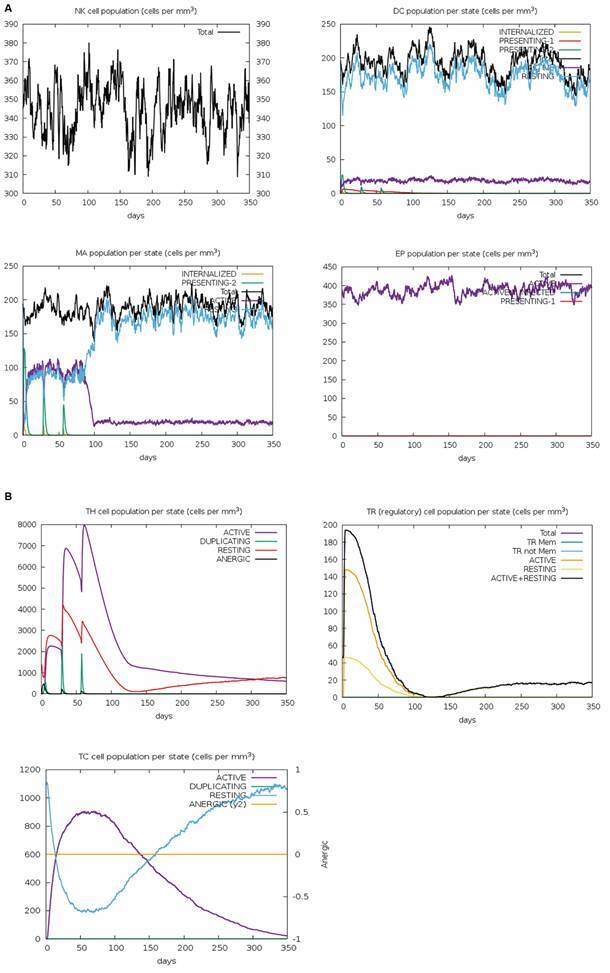
graphs of the *in silico* immunological simulation generated using the C-Imm-Simm program about the multiepitope vaccine with adjuvant. In panel A, the simulation data for the populations of natural killer cells (NK), dendritic cells (DC), macrophages (MA), and epithelial cells (EP) are represented. In panel B, the simulation data for the populations of helper T cells (TH), regulatory T cells (Treg), and cytotoxic T cells (TC) are shown.

**Fig. 7:**
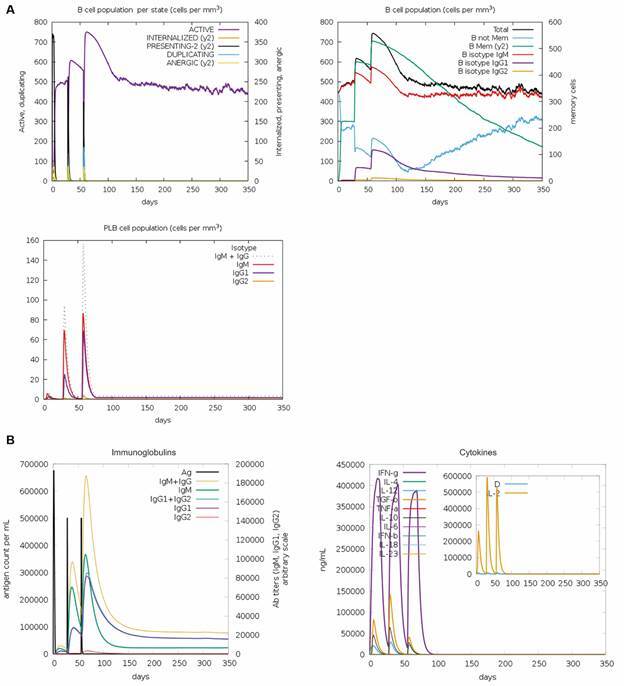
graphs of the *in silico* immunological simulation generated using the C-Imm-Simm program about the multiepitope vaccine without adjuvant. The simulation data for B cell populations and plasma B cells are depicted in panel A. The simulation data related to immunoglobulins and cytokines are shown in panel B.

*A candidate vaccine with the multi-epitope protein may elicit a robust immune* response - The IFNepitope program identified 559 epitopes. Among these, 153 were confirmed as positive, inducing IFN-gamma production, while 406 were negative, failing to induce this cytokine [Supplementary data (Table V)]. The sequence of our multi-epitope protein was submitted, without adjuvant, in IL6pred and TNFepitope for prediction of the Interleukin-6 and Tumour Necrosis Factor Alpha, respectively. The IL6pred software identified 397 epitopes, which among these, 28 were confirmed as positive for IL6 induction and 369 were confirmed as negative for IL6 induction [Supplementary data (Table VI)]. The TNFepitope also identified 397 epitopes, which among these, 102 were classified as TNF-inducer and 295 as TNF non-inducer [Supplementary data (Table VII)]. The results of the immune simulation show that a vaccine based on our protein can stimulate the activation of natural killer cells throughout the time after injection and macrophages after the first and second doses. Dendritic cells mostly appear resting, but a portion remains active after injection. Epithelial cells appear busy throughout time (Fig. 6A). The prediction indicated increased populations of helper T cells, regulatory T cells, and cytotoxic T cells during vaccine administration, with greater activation of helper T cells, followed by cytotoxic T cells, and regulatory T cells (Fig. 6B). The administration of this vaccine also strongly demonstrated an increase in active memory B cell populations throughout the *in silico* injection period, accompanied by a decrease in non-memory B cell populations and an increase in the levels of IgM and IgG1 B cell isotypes (Fig. 7A). Finally, an increase in immunoglobulins, primarily the sum of IgM and IgG, was observed, along with a significant rise in IFN-γ and a modest increase in TGF-β, followed by a decrease in all cytokines after vaccine administration (Fig. 7B). To conclude, we also performed an *in silico* simulation of vaccine administration composed solely of the adjuvant to evaluate our multi-epitope protein [Supplementary data (Figure)]. The simulation revealed reduced innate immune cells, particularly dendritic cells [Supplementary data (Figure A)]. T cell populations were also activated, although they were smaller in number, and cytotoxic T cells remained resting compared to the multi-epitope protein [Supplementary data (Figure B)]. A similar stimulation pattern was observed for B cells, albeit with lower B cell production and fewer B cells related to the presentation state [Supplementary data (Figure C)]. Finally, cytokine production followed a similar pattern, unlike immunoglobulin production, which seemed to have different mechanisms, with stimulation more dependent on the adjuvant [Supplementary data (Figure D)].

## DISCUSSION

Vaccination is widely recognised as the most effective method for preventing a wide range of diseases. Recent approaches to vaccine development, such as subunit vaccines, are rooted in immunoinformatic studies. These approaches are gaining prominence due to their advantages, including accelerated vaccine production and the identification of more effective immunogens. Among subunit vaccines, the so-called multi-epitope vaccine stands out. This type of vaccine allows for the selection of immunogens capable of stimulating both innate and adaptive immune responses, further promoting the development of adaptive memory.^37^ ^,^ ^38^

Immunoinformatics tools were employed to identify the best epitopes within the core genome proteins. This selection is crucial as it encompasses diverse species and lineages, minimising the likelihood of bacterial mutations leading to immune resistance. Those core epitopes were described as potential vaccine candidates against *Rickettsia* bacteria by Felice et al.^33^ Core genome proteins, often referred to as central proteins, play a pivotal role in representing the entire set of conserved genes in organisms under study. This enables us to gain insights into species diversity within this set and, consequently, how they behave.^46^

Researchers’ primary concern in developing vaccines, researchers’ primary concern is the vaccine’s efficacy for the global population. This work adhered to this premise by selecting the most commonly found worldwide MHC alleles. Subsequently, epitopes of the proteins that bind to MHCI/CTL, MHCII/HTL complexes, and B cells were chosen. This approach aims to stimulate both cellular and humoral immunity. Consequently, 26 epitopes from seven proteins were selected to construct a multi-epitope protein for a chimeric peptide vaccine against *Rickettsia* bacteria.

These 26 epitopes were derived from the following proteins: WP_012148219.1, WP_012149174.1, and WP_012149175.1, belonging to the Porin family; WP_012148227.1, a member of the OmpW family; WP_012148552.1, known as a copper chaperone; WP_012148811.1, a dioxygenase; and finally, WP_012148819.1, a D-alanyl-D-alanine carboxypeptidase. The first four proteins belong to a more prominent family called outer membrane proteins (OMPs), found within Gram-negative bacteria’s outer membrane sheets. These OMPs form barrel/porin structures within the membrane that serve various functions, including aiding in membrane structure, cell adhesion, lipid metabolism, and the transport of hydrophilic substances. This transport is hindered by the presence of lipopolysaccharides in the outer membrane.^90^ ^,^ ^91^ These functions have been extensively explored as data have shown that these channels assist in the pathogenesis of specific organisms, conferring resistance related to the survival of these organisms by inhibiting or facilitating the transport of substances across the membrane.^92^

Chaperones have functions related to the degradation of unwanted intracellular components, crucial for cellular adaptation to environmental changes and responses to various types of stresses from the intra or extracellular milieu.^93^ Dioxygenases and carboxypeptidases are biochemical molecules that initiate the degradation and cleavage of aromatic compounds and basic amino acids metabolically active in bacterial cell survival processes.^94^ ^,^ ^95^ All functions associated with these proteins demonstrate interactions in the pathogen/host relationship and the diseases caused. This supports the idea that the vaccine based on the selected epitopes enhances efficacy and the induction of a robust immune response in the global population against rickettsiae. Additionally, ligands were used between the epitopes chosen in the final protein to enhance antigen processing and presentation within the host, aid in structural flexibility, and reduce the likelihood of junctional antigen formation.^58^

It is known that classical vaccines may use the entire pathogen, which can possess proteins similar to those of the host, inducing various types of adverse reactions. Following the same line as the vaccines in this work, our protein was carefully designed to have no homology with the host, thus avoiding such adverse reactions. The protein was also evaluated for toxicity and allergenicity, and it was considered non-toxic and non-allergenic to humans. Another way to assess interaction with the host is related to factors such as half-life, molecular dynamics, and vaccine solubility. A low half-life or solubility could jeopardise this protein’s practical application as a prevention measure.^96^ ^,^ ^97^ Our construct had a half-life of at least 10 h in *E. coli* cells *in vivo*, with a solubility of 0.427, below the threshold of 0.45; it was considered stable and had a hydropathicity index of -0.089. Although our multi-epitope vaccine contradicts findings from works such as Vilela-Rodrigues et al.^40^ and Nandi^98^ and presents this limitation regarding hydropathicity, tending to interact with an aqueous environment, the other results seem to align with their patterns, suggesting that it may be considered a promising vaccine for *in vitro* and *in vivo* testing.

The interaction stage with host cells and its underlying mechanisms are critical for understanding the inflammatory process triggered by these bacteria’s infection. Mechanisms such as adhesion to host cells, phagocytosis induction, acquisition, or degradation of nutrients, for example, resulting from the functions of proteins used in our vaccine, help to understand the inflammatory process generated as an immune response.^99^

The method of biological damage and the production of reactive oxygen species is initiated whenever the pathogen is recognised by Toll-like receptors^74^ ^,^ ^100^ ^,^ ^101^ and results in inflammatory cytokines, such as, mainly, IL6, TNF-α and IFN-γ.^73^ ^,^ ^75^ ^,^ ^102^ According to the literature, both TLR2 and TLR4 are known as good Toll-like receptors for interaction in rickettsiosis, however TLR2 could be present a more exacerbated response and cause pathological effects.^75^ The TLR4 receptor was used for the docking analyses with our vaccine, and the interaction region of complex TLR4 - RGME-VAC/ATS-1 show to have a flexibility but with a good stability when subjected to the movements of molecular dynamics, if they are compared with the flexibility of the interaction region of the only TLR-4 alone when subjected to the same movements of molecular dynamics. Also, the two molecular dynamics performed show a maintained temperature (around 300k) and pressure stability (1 bar) within a system that simulates something similar to what the molecules would face in a bacterial infection process in their host. In addition, the residues had a small fluctuation in the RMSF tests (0.04 nm to 0.21 nm from the initial position) and little change in position by the RMSD test (0.5 nm to 3 nm from the initial position) when are analysed the movements of complex TLR4 - RGME-VAC/ATS-1 in relation of only TLR-4 alone. These stability results could suggest that our protein is able to block the mobility of the receptor in the interaction region.

Based on this, we resolved tested, also *in silico*, if our vaccine, when recognised by TLR, be able to stimulate an immune response. At first, the RGME-VAC/ATS-1 was be able to stimulate, during the injection times, cells as macrophages, natural killers and dendritic. Several studies have already managed to prove that these cells have to a bacterial activity when there is the presence of a rickettsial infection, including being more active due to the presence of the aforementioned cytokines.^76^ ^,^ ^103^ ^,^ ^104^ These cells are recruited and directed to the site of infection, in rickettsiosis’ case, endothelial cells, and then perform the phagocytosis of infected cells, which are mainly responsible for the innate immune response.^105^ After this, our vaccine also was tested in terms of its capacity to induce the mainly cytokines. A diversity of epitopes of our vaccine presents a capacity of induce IL6, TNF-α and IFN-γ, and then, when recognise by toll-like receptors, are capable of stimulating vias as Nf-κB, and induce several others inflammatory cytokines and others cells that are very important in innate or adaptative response.^105^

The RGME-VAC/ATS-1 also was be able to stimulate a possible adaptative immune response, because, also during the injection time, there is an increase of T Helper, T cytotoxic, T regulatory and B cells. T and B cells are the main cell types responsible for the adaptive immune response. They are recruited to the site of infection, and through the release of cytokines act to eliminate pathogens.^105^ A range of studies involving rickettsial infections have shown the importance of these cells in fighting the infection,^106^ ^,^ ^107^ ^,^ ^108^ which shows that the actions of releasing effector molecules by T helper cells and the cytotoxic action of the cytotoxic T cell itself, are the main responsible for controlling the infection.

Finally, the action of B cells becomes very important, especially when it comes to *R. prowazekii*, as the typhus caused by it can reactivate after many years and cause Brill-Zinsser disease.^109^ ^,^ ^110^ B-cells are responsible for producing antibodies and maybe memory cells, that could be in a response faster after a reinfection. Some works show that the antibodies produced in the presence of T-cells provide better protection than those generated without them,^103^ ^,^ ^111^ ^,^ ^112^ which is another reason to consider RGME-VAC/ATS-1 as a good candidate for fighting *Rickettsia* infections.

*Rickettsia* species are responsible for various human diseases in humans, such as spotted fevers and typhus. These diseases are considered highly contagious, and there are currently no preventive measures that are genuinely effective against them. As a result, the development of a vaccine is the main goal of researchers of the field to reduce their prevalence worldwide. This study used an *in silico* immunoinformatic approach to construct a multi-epitope vaccine effective against all *Rickettsia* species, generating innate and adaptive immune responses in the host.

In general, all *in silico* validations performed here support the idea that our vaccine is a strong candidate to act against infection caused by these bacteria, which not show toxic and allergic potential when compared to the human host and showed good physio-chemical parameters. In addition, after the *in silico* immune simulation tests, the vaccine showed good stimulation of T cells and memory-generating B cells; also, several epitopes of our protein had great capacity to induce the production of cytokines such as IFN-γ, IL6 and TNF. All these cells and molecules are consistent with the Th1-type immune response pattern, which is much more stimulated when the microorganisms are obligate intracellular organisms, as is the case with species of the *Rickettsia* genus.

However, *in vitro* and *in vivo*, further validations are needed to confirm its efficacy and safety in humans.

## References

[1] Lima DS, Farias EVS, Ferreira DA, Nascimento do LEA, Luis JAS, Lima IO (2020). Aspectos do gênero Rickettsia uma revisão sistemática. Educ Ci e Sal.

[2] Schroeder CLC, Narra HP, Rojas M, Sahni A, Patel J, Khanipov K (2015). Bacterial small rnas in the genus Rickettsia. BMC Genomics.

[3] Parola P, Paddock CD, Socolovschi C, Labruna MB, Mediannikov O, Kernif T (2013). Update on tick-borne rickettsioses around the world a geographic approach. Clin Microbiol Rev.

[4] Blanton LS (2019). The rickettsioses a pratical update. Infect Dis Clin North Am.

[5] Galvão MAM, Silva LJ, Nascimento EMM, Calic SB, Sousa R, Bacellar F (2005). Riquetsioses no Brasil e Portugal ocorrência, distribuição e diagnóstico. Rev Saúde Publica.

[6] Gillespie JJ, Beier MS, Rahman MS, Ammerman NC, Shallom JM, Purkayastha A (2007). Plasmids and rickettsial evolution insight from Rckettsia felis. PLoS One.

[7] Stothard DR, Fuerst PA (1995). Evolutionary analysis of the spotted fever and thyphus groups of Rickettsia using 16S rrna gene sequences. Syst Appl Microbiol.

[8] Davoust B, Mediannikov O, Marie JL, Socolovschi C, Parola P, Raoult D (2010). Are vertebrates reservoir hosts for Rickettsia. Acad Vet.

[9] Colonne PM, Eremeeva ME, Sahni SK (2011). Beta interferon-mediated activation of signal transducer and activator of transcription protein 1 interferes with Rickettsia conorii replication in human endothelial cells. Infect Immun.

[10] Rydkina E, Turpin LC, Sahni SK (2010). Rickettsia rickettsii infection of human macrovascular and microvascular endothelial cells reveals activation of both common and cell type-specific host response mechanisms. Infect Immun.

[11] Gaywee J, Sunyakumthorn P, Rodkvamtook W, Ruang-areerate T, Mason CJ, Sirisopana N (2007). Human infection with Rickettsia sp related to R. japonica, Thailand. Emerg Infect Dis.

[12] Bermúdez S, Troyo A (2018). A review of the genus Rickettsia in Central America. Res Rep Trop Med.

[13] Angelakis E, Bechah Y, Raoult D (2016). The history of epidemic typhus. Microbiol Spectr.

[14] Burns JN, Acuna-Soto R, Stahle DW (2014). Drought and epidemic typhus, central Mexico, 1655-1918. Emerg Infect Dis.

[15] Kuo CC, Wardrop N, Chang CT, Wang HC, Atkinson PM (2017). Significance of major international seaports in the distribution of murine typhus in Taiwan. PLoS Negl Trop Dis.

[16] Blanton LS, Walker DH (2017). Flea-borne rickettsioses and Rickettsiae. Am J Trop Med Hyg.

[17] Tarragona EL, Soares JF, Costa FB, Labruna MB, Nava S (2016). Vectorial competence of Amblyomma tonelliae to transmit Rickettsia rickettsii. Med Vet Entomol.

[18] Straily A, Drexler N, Cruz-Loustaunau D, Paddock CD, Alvarez-Hernandez G (2016). Notes from the field community-based prevention of rocky mountain spotted fever - Sonora, Mexico, 2016. MMWR Morb Mortal Wkly Rep.

[19] Fang R, Blanton LS, Walker DH (2017). Rickettsiae as emerging infectious agents. Clin Lab Med.

[20] Peniche-Lara G, Zavala-Velazquez J, Dzul-Rosado K, Walker DH, Zavala-Castro J (2013). Simple method to differentiate among Rickettsia species. J Mol Microbiol Biotechnol.

[21] Biggs HM, Behravesh CB, Bradley KK, Dahlgren FS, Drexler NA, Dumler JS (2016). Diagnosis and management of tickborne rickettsial diseases rocky mountain spotted fever and other spotted fever group rickettsioses, ehrlichioses, and anaplasmosis - United States. MMWR Recomm Rep.

[22] Vanrompay D, Nguyen TLA, Cutler SJ, Butaye P (2018). Antimicrobial resistance in Chlamydiales, Rickettsia, Coxiella, and other intracellular pathogens. Microbiol Spectr.

[23] Akram SM, Ladd M, King KC (2022). StatPearls.

[24] Osterloh A (2021). Vaccine design and vaccination strategies against Rickettsiae. Vaccines (Basel).

[25] Walker DH (2009). The realities of biodefense vaccines against Rickettsia. Vaccine.

[26] Weigl R (1947). Immunization against typhus fever in Poland during World War II. Tex Rep Biol Med.

[27] Gonder JC, Kenyon RH, Pedersen CE (1979). Evaluation of a killed rocky mountain spotted fever vaccine in Cynomolgus monkeys. J Clin Microbiol.

[28] Osterloh A (2020). The neglected challenge vaccination against Rickettsiae. PLoS Negl Trop Dis.

[29] Clements ML, Wisseman CL, Woodward TE, Fiset P, Dumler JS, McNamee W (1983). Reactogenicity, immunogenicity, and efficacy of a chick embryo cell-derived vaccine for rocky mountain spotted fever. J Infect Dis.

[30] Vaughn MF, Funkhouser SW, Lin FC, Fine J, Juliano JJ, Apperson CS (2014). Long-lasting permethrin impregnated uniforms. Am J Prev Med.

[31] Mosmann TR, Sad S, Krishnan L, Wegmann TG, Guilbert LJ, Belosevic M (2007). Differentiation of subsets of CD4 + and CD8 + T Cells. Ciba Found Symp.

[32] Annunziato F, Romagnani C, Romagnani S (2015). The 3 major types of innate and adaptive cell-mediated effector immunity. J Allergy Clin Immunol.

[33] Felice AG, Alves LG, Freitas ASF, Rodrigues TCV, Jaiswal AK, Tiwari S (2021). Pan-genomic analyses of 47 complete genomes of the Rickettsia genus and prediction of new vaccine targets and virulence factors of the species. J Biomol Struct Dyn.

[34] Prado LCS, Felice AG, Rodrigues TCV, Tiwari S, Andrade BS, Kato RB (2021). New putative therapeutic targets against Serratia marcescens using reverse vaccinology and subtractive genomics. J Biomol Struct Dyn.

[35] Felice AG, Santos LNQ, Kolossowski I, Zen FL, Alves LG, Rodrigues TCV (2021). Comparative genomics of Bordetella pertussis and prediction of new vaccines and drug targets. J Biomol Struct Dyn.

[36] Chauhan V, Rungta T, Goyal K, Singh MP (2019). Designing a multi-epitope based vaccine to combat Kaposi Sarcoma utilizing immunoinformatics approach. Sci Rep.

[37] Sette A, Fikes J (2003). Epitope-based vaccines an update on epitope identification, vaccine design and delivery. Curr Opin Immunol.

[38] Sette A, Newman M, Livingston B, McKinney D, Sidney J, Ishioka G (2002). Optimizing vaccine design for cellular processing, MHC binding and TCR recognition. Tissue Antgens.

[39] Hajighahramani N, Nezafat N, Eslami M, Negahdaripour M, Rahmatabadi SS, Ghasemi Y (2017). Immunoinformatics analysis and in silico designing of a novel multi-epitope peptide vaccine against Staphylococcus aureus. Infect Genet Evol.

[40] Rodrigues TCV, Jaiswal AK, Lemes MR, da Silva MV, Sales-Campos H, Alcântara LCJ (2022). An immunoinformatics-based designed multi-epitope candidate vaccine (mpme-VAC/STV-1) against Mycoplasma pneumoniae. Comput Biol Med.

[41] Lemes MR, Rodrigues TCV, Jaiswal AK, Tiwari S, Sales-Campos H, Andrade-Silva LE (2022). In silico designing of a recombinant multi-epitope antigen for leprosy diagnosis. J Genet Eng Biotechnol.

[42] Lu Y, Ding J, Liu W, Chen YH (2002). A candidate vaccine against influenza virus intensively improved the immunogenicity of a neutralizing epitope. Int Arch Allergy Immunol.

[43] Tosta SFO, Passos MS, Kato R, Salgado Á, Xavier J, Jaiswal AK (2021). Multi-epitope based vaccine against yellow fever virus applying immunoinformatics approaches. J Biomol Struct Dyn.

[44] Zhou WY, Shi Y, Wu C, Zhang WJ, Mao XH, Guo G (2009). Therapeutic efficacy of a multi-epitope vaccine against Helicobacter pylori infection in BALB/c mice model. Vaccine.

[45] He R, Yang X, Liu C, Chen X, Wang L, Xiao M (2018). Efficient control of chronic LCMV infection by a CD4 T cell epitope-based heterologous prime-boost vaccination in a murine model. Cell Mol Immunol.

[46] Juncker AS, Larsen MV, Weinhold N, Nielsen M, Brunak S, Lund O (2009). Systematic characterisation of cellular localisation and expression profiles of proteins containing MHC ligands. PLoS One.

[47] Vita R, Mahajan S, Overton JA, Dhanda SK, Martini S, Cantrell JR (2019). The Immune Epitope Database (IEDB) 2018 update. Nucleic Acids Res.

[48] Larsen MV, Lundegaard C, Lamberth K, Buus S, Lund O, Nielsen M (2007). Large-scale validation of methods for cytotoxic T-lymphocyte epitope prediction. BMC Bioinformatics.

[49] Greenbaum J, Sidney J, Chung J, Brander C, Peters B, Sette A (2011). Functional classification of class II human leukocyte antigen (HLA) molecules reveals seven different supertypes and a surprising degree of repertoire sharing across supertypes. Immunogenetics.

[50] Jensen KK, Andreatta M, Marcatili P, Buus S, Greenbaum JA, Yan Z (2018). Improved methods for predicting peptide binding affinity to MHC class II molecules. Immunology.

[51] Rana A, Akhter Y (2016). A multi-subunit based, thermodynamically stable model vaccine using combined immunoinformatics and protein structure based approach. Immunobiology.

[52] Chen J, Liu H, Yang J, Chou KC (2007). Prediction of linear B-cell epitopes using amino acid pair antigenicity scale. Amino Acids.

[53] El-Manzalawy Y, Dobbs D, Honavar V (2008). Predicting flexible length linear B-cell epitopes. Comput Syst Bioinformatics Conf.

[54] Saha S, Raghava GPS (2006). Prediction of continuous B-cell epitopes in an antigen using recurrent neural network. Proteins.

[55] Gupta S, Kapoor P, Chaudhary K, Gautam A, Kumar R, Raghava GPS (2013). In silico approach for predicting toxicity of peptides and proteins. PLoS One.

[56] Calis JJA, Maybeno M, Greenbaum JA, Weiskopf D, De Silva AD, Sette A (2013). Properties of MHC class I presented peptides that enhance immunogenicity. PLoS Comput Biol.

[57] Doytchinova IA, Flower DR (2007). VaxiJen a server for prediction of protective antigens, tumour antigens and subunit vaccines. BMC Bioinformatics.

[58] Chen X, Zaro JL, Shen WC (2013). Fusion protein linkers property, design and functionality. Adv Drug Deliv Rev.

[59] Ma Y (2016). Recent advances in nontoxic Escherichia coli heat-labile toxin and its derivative adjuvants. Expert Rev Vaccines.

[60] Li G, Huang Z, Zhang C, Dong BJ, Guo RH, Yue HW (2016). Construction of a linker library with widely controllable flexibility for fusion protein design. Appl Microbiol Biotechnol.

[61] Choi S, Jeong HJ, Ju YR, Gill B, Hwang KJ, Lee J (2014). Protective immunity of 56-kDa type-specific antigen of Orientia tsutsugamushi causing scrub typhus. J Microbiol Biotechnol.

[62] Choi S, Jeong HJ, Hwang KJ, Gill B, Ju YR, Lee YS (2017). A recombinant 47-kDa outer membrane protein induces an immune response against Orientia tsutsugamushi strain boryong. Am Soc Trop Med Hyg.

[63] Ward JJ, McGuffin LJ, Buxton BF, Jones DT (2003). Secondary structure prediction with support vector machines. Bioinformatics.

[64] Mirdita M, Schütze K, Moriwaki Y, Heo L, Ovchinnikov S, Steinegger M (2022). ColabFold making protein folding accessible to all. Nat Methods.

[65] Heo L, Park H, Seok C (2013). GalaxyRefine protein structure refinement driven by side-chain repacking. Nucleic Acids Res.

[66] Laskowski RA, MacArthur MW, Moss DS, Thornton JM (1993). PROCHECK a program to check the stereochemical quality of protein structures. J Appl Crystallogr.

[67] Ponomarenko J, Bui HH, Li W, Fusseder N, Bourne PE, Sette A (2008). ElliPro a new structure-based tool for the prediction of antibody epitopes. BMC Bioinformatics.

[68] He Y, Xiang Z, Mobley HLT (2010). Vaxign the first web-based vaccine design program for reverse vaccinology and applications for vaccine development. J Biomed Biotechnol.

[69] Dimitrov I, Bangov I, Flower DR, Doytchinova I (2014). AllerTOP v 2 - a server for in silico prediction of allergens. J Mol Model.

[70] Wilkins MR, Gasteiger E, Bairoch A, Sanchez JC, Williams KL, Appel RD (1999). Protein identification and analysis tools on the ExPASy server. Methods Mol Biol.

[71] Hebditch M, Carballo-Amador MA, Charonis S, Curtis R, Warwicker J (2017). Protein-Sol a web tool for predicting protein solubility from sequence. Bioinformatics.

[72] Altschul SF, Gish W, Miller W, Myers EW, Lipman DJ (1990). Basic local alignment search tool. J Mol Biol.

[73] Jordan JM, Woods ME, Olano J, Walker DH (2008). The absence of toll-like receptor 4 signaling in C3H/HeJ mice predisposes them to overwhelming rickettsial infection and decreased protective Th1 responses. Infect Immun.

[74] Rumfield C, Hyseni I, McBride JW, Walker DH, Fang R (2020). Activation of ASC Inflammasome driven by toll-like receptor 4 contributes to host immunity against Rickettsial infection. Infect Immun.

[75] Osterloh A (2017). Immune response against Rickettsiae lessons from murine infection models. Med Microbiol Immunol.

[76] Berman HM (2000). The protein data bank. Nucleic Acids Res.

[77] Pettersen EF, Goddard TD, Huang CC, Couch GS, Greenblatt DM, Meng EC (2004). UCSF Chimera - A visualization system for exploratory research and analysis. J Comput Chem.

[78] Kozakov D, Hall DR, Xia B, Porter KA, Padhorny D, Yueh C (2017). The ClusPro web server for protein-protein docking. Nat Protoc.

[79] Abraham MJ, Murtola T, Schulz R, Páll S, Smith JC, Hess B (2015). GROMACS: high performance molecular simulations through multi-level parallelism from laptops to supercomputers. SoftwareX.

[80] Huang J, MacKerell AD (2013). CHARMM36 all-atom additive protein force field Validation based on comparison to NMR data. J Comput Chem.

[81] Grote A, Hiller K, Scheer M, Munch R, Nortemann B, Hempel DC (2005). JCat: a novel tool to adapt codon usage of a target gene to its potential expression host. Nucleic Acids Res.

[82] SnapGene (2024). SnapGene.

[83] Dhanda SK, Vir P, Raghava GP (2013). Designing of interferon-gamma inducing MHC class-II binders. Biol Direct.

[84] Dhall A, Patiyal S, Sharma N, Usmani SS, Raghava GPS (2023). A web-based method for the identification of IL6-based immunotoxicity in vaccine candidates. Methods Mol Biol.

[85] Dhall A, Patiyal S, Choudhury S, Jain S, Narang K, Raghava GPS (2023). TNFepitope a webserver for the prediction of TNF-a inducing epitopes. Comput Biol Med.

[86] Rapin N, Lund O, Bernaschi M, Castiglione F (2010). Computational immunology meets bioinformatics the use of prediction tools for molecular binding in the simulation of the immune system. PLoS One.

[87] Strikas RA (2015). Advisory committee on immunization practices recommended immunization schedules for persons aged 0 through 18 years - United States, 2015. MMWR Morb Mortal Wkly Rep.

[88] Castiglione F, Mantile F, De Berardinis P, Prisco A (2012). How the interval between prime and boost injection affects the immune response in a computational model of the immune system. Comput Math Methods Med.

[89] Qamar MTU, Rehman A, Tusleem K, Ashfaq UA, Qasim M, Zhu X (2020). Designing of a next generation multiepitope based vaccine (MEV) against SARS-COV-2 immunoinformatics and in silico approaches. PLoS One.

[90] De la Cruz MA.Calva E (2010). The complexities of porin genetic regulation. Microb Physiol.

[91] Hong H, Patel DR, Tamm LK, van den Berg B (2006). The outer membrane protein OmpW forms an eight-stranded ß-barrel with a hydrophobic channel. J Biol Chem.

[92] Saier-Junior MH (2007). Active transport in communication, protection and nutrition. J Mol Microbiol Biotechnol.

[93] Bejarano E, Cuervo AM (2010). Chaperone-mediated autophagy. Proc Am Thorac Soc.

[94] Doble M, Kumar A (2005). Biotreatment of industrial effluents.

[95] Matthews KW, Mueller-Ortiz SL, Wetsel RA (2004). Carboxypeptidase N a pleiotropic regulator of inflammation. Mol Immunol.

[96] Mathur D, Prakash S, Anand P, Kaur H, Agrawal P, Mehta A (2016). PEPlife a repository of the half-life of peptides. Sci Rep.

[97] Smialowski P, Martin-Galiano AJ, Mikolajka A, Girschick T, Holak TA, Frishman D (2007). Protein solubility sequence based prediction and experimental verification. Bioinformatics.

[98] Nandi A, Solanki V, Tiwari V, Srivastava S, Ghosh S (2022). Designing of multi-epitope vaccine construct employing immuno-informatics approach to combat multi-tick species infestations. Preprint.

[99] Walker DH, Valbuena GA, Olano JP (2003). Pathogenic mechanisms of diseases caused by Rickettsia. Ann N Y Acad Sci.

[100] Jordan JM, Woods ME, Soong L, Walker DH (2009). Rickettsiae stimulate dendritic cells through toll-like receptor 4, leading to enhanced NK cell activation in vivo. J Infect Dis.

[101] Quevedo-Diaz MA, Song C, Xiong Y, Chen H, Wahl LM, Radulovic S (2010). Involvement of TLR2 and TLR4 in cell responses to Rickettsia akari. J Leukoc Biol.

[102] Turco J, Winkler HH (2002). Rickettsial infection and immunity.

[103] Papp S, Moderzynski K, Rauch J, Heine L, Kuehl S, Richardt U (2016). Liver necrosis and lethal systemic inflammation in a murine model of Rickettsia typhi infection role of neutrophils, macrophages and NK cells. PLoS Negl Trop Dis.

[104] Feng HM, Walker DH (2000). Mechanisms of intracellular killing of Rickettsia conorii in infected human endothelial cells, hepatocytes, and macrophages. Infect Immun.

[105] Abbas AK, Lichtman AH, Pillai S (2015). Imunologia celular e molecular.

[106] Moderzynski K, Papp S, Rauch J, Heine L, Kuehl S, Richardt U (2016). CD4+ T cells are as protective as CD8+ T cells against Rickettsia typhi infection by activating macrophage bactericidal activity. PLoS Negl Trop Dis.

[107] Walker DH, Olano JP, Feng HM (2001). Critical role of cytotoxic T lymphocytes in immune clearance of Rickettsial infection. Infect Immun.

[108] Moderzynski K, Heine L, Rauch J, Papp S, Kuehl S, Richardt U (2017). Cytotoxic effector functions of T cells are not required for protective immunity against fatal Rickettsia typhi infection in a murine model of infection role of TH1 and TH17 cytokines in protection and pathology. PLoS Negl Trop Dis.

[109] Lutwick L (2001). Brill-Zinsser disease. Lancet.

[110] Stein A, Purgus R, Olmer M, Raoult D (1999). Brill-Zinsser disease in France. Lancet.

[111] Feng HM, Whitworth T, Olano JP, Popov VL, Walker DH (2004). Fc-dependent polyclonal antibodies and antibodies to outer membrane proteins A and B, but not to lipopolysaccharide, protect SCID mice against fatal Rickettsia conorii infection. Infect Immun.

[112] Osterloh A, Papp S, Moderzynski K, Kuehl S, Richardt U, Fleischer B (2016). Persisting Rickettsia typhi causes fatal central nervous system inflammation. Infect Immun.

