# Supplementary material for: In silico construction of a multi-epitope vaccine (RGME-VAC/ATS-1) against the Rickettsia genus using immunoinformatics
Source: Mem Inst Oswaldo Cruz. 2025 Mar 21;120:e240201. doi: 10.1590/0074-02760240201 (PMC11932644; doi:10.1590/0074-02760240201)
Supplement: Supplementary file 1 [file 1678-8060-mioc-120-e240201-s.pdf]

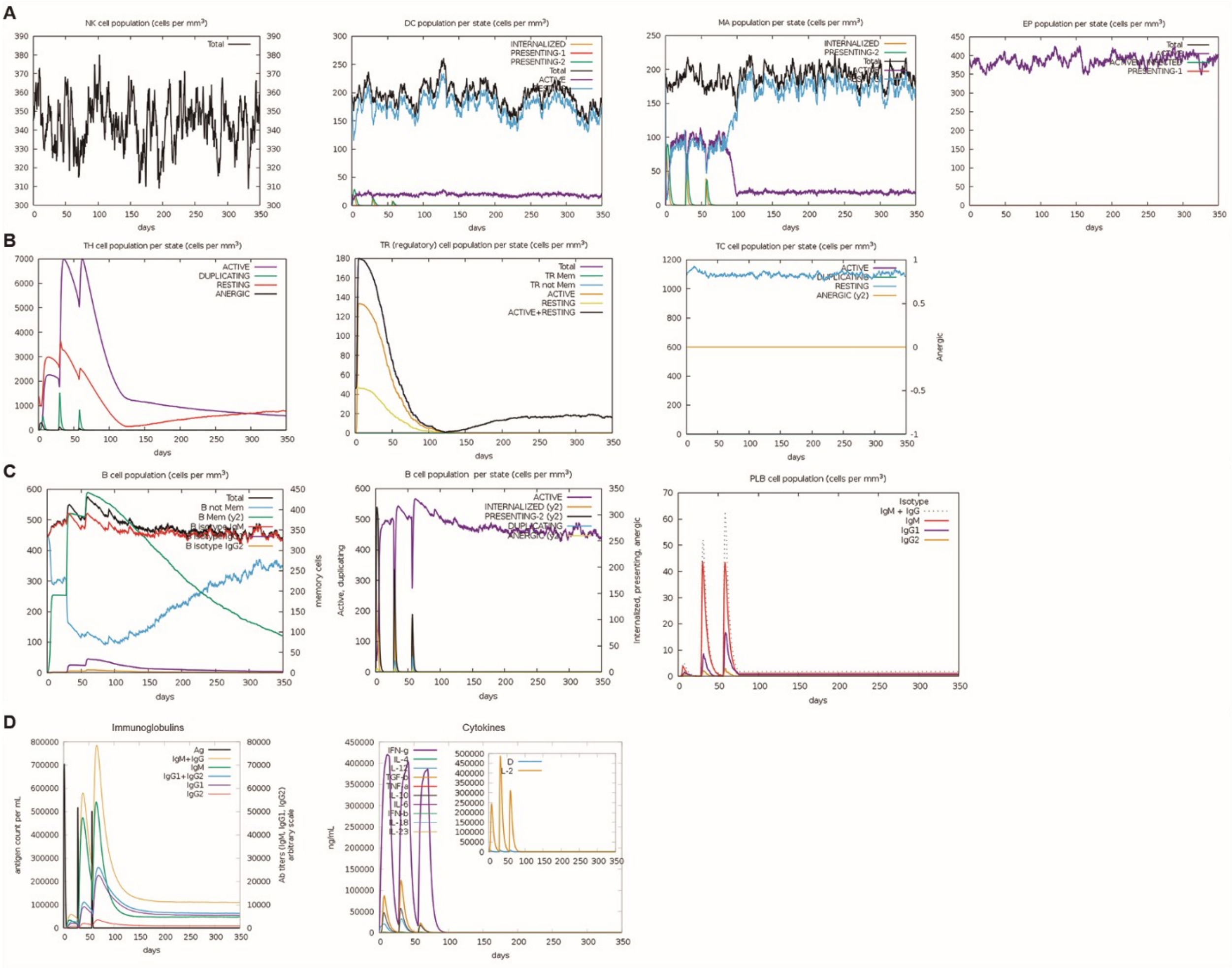

In (A), the simulation data for the populations of natural killer (NK) cells, dendritic cells (DC), macrophages (MA), and epithelial cells (EP) are represented. In (B), simulation data for the populations of T helper cells (TH), regulatory T cells (Treg), and cytotoxic T cells (TC) are represented. In (C), the simulation data for B-cell and B-cell populations in plasma are plotted. In (D), the simulation data related to immunoglobulins and cytokines, respectively, are represented.

cc BY

TABLE I

List of the eight proteins considered as candidate vaccine targets by the literature. The table below shows the National Center of Biotechnology Information (NCBI) database ID and the name of each of the proteins

ID protein	Name
WP_012148219.1	Porine
WP_012148227.1	OmpW protein family
WP_012148552.1	Copper chaperone PCu(A)C
WP_012148811.1	Dioxygenase
WP_012148819.1	D-alanyl-D-alanine carboxypeptidase
WP_012149174.1	Porine family of proteins
WP_012149175.1	Porins protein family
WP_012149185.1	Alpha/beta hydrolase

ID: identity.

TABLE II

Alleles used in each analysis for the stage epitope prediction in this work

Alleles	
IEDB - MHC I binding epitope	HLA-A*01:01 9<br>HLA-A*02:01 9<br>HLA-A*02:03 9<br>HLA-A*02:06 9<br>HLA-A*03:01 9<br>HLA-A*11:01 9<br>HLA-A*23:01 9<br>HLA-A*24:02 9<br>HLA-A*26:01 9<br>HLA-A*30:01 9<br>HLA-A*30:02 9<br>HLA-A*31:01 9<br>HLA-A*32:01 9<br>HLA-A*33:01 9<br>HLA-A*68:01 9<br>HLA-A*68:02 9<br>HLA-B*07:02 9<br>HLA-B*08:01 9<br>HLA-B*15:01 9<br>HLA-B*35:01 9<br>HLA-B*40:01 9<br>HLA-B*44:02 9<br>HLA-B*44:03 9<br>HLA-B*51:01 9<br>HLA-B*53:01 9<br>HLA-B*57:01 9<br>HLA-B*58:01 9
NetCTL 1.2	Supertypes A1, A2, A3, A24, A26, B7, B8, B44 and B58

IEDB – MHC II binding epitope	HLA-DRB1*01:01<br>HLA-DRB1*03:01<br>HLA-DRB1*04:01<br>HLA-DRB1*04:05<br>HLA-DRB1*07:01<br>HLA-DRB1*08:02<br>HLA-DRB1*09:01<br>HLA-DRB1*11:01<br>HLA-DRB1*12:01<br>HLA-DRB1*13:02<br>HLA-DRB1*15:01<br>HLA-DRB3*01:01<br>HLA-DRB3*02:02<br>HLA-DRB4*01:01<br>HLA-DRB5*01:01<br>HLA-DQA1*05:01/DQB1*02:01<br>HLA-DQA1*05:01/DQB1*03:01<br>HLA-DQA1*03:01/DQB1*03:02<br>HLA-DQA1*04:01/DQB1*04:02<br>HLA-DQA1*01:01/DQB1*05:01<br>HLA-DQA1*01:02/DQB1*06:02<br>HLA-DPA1*02:01/DPB1*01:01<br>HLA-DPA1*01:03/DPB1*02:01<br>HLA-DPA1*01:03/DPB1*04:01<br>HLA-DPA1*03:01/DPB1*04:02<br>HLA-DPA1*02:01/DPB1*05:01<br>HLA-DPA1*02:01/DPB1*14:01

NetMHCII 2.3	HLA-DRB1*01:01<br>HLA-DRB1*03:01<br>HLA-DRB1*04:01<br>HLA-DRB1*04:05<br>HLA-DRB1*07:01<br>HLA-DRB1*08:02<br>HLA-DRB1*09:01<br>HLA-DRB1*11:01<br>HLA-DRB1*12:01<br>HLA-DRB1*13:02<br>HLA-DRB1*15:01<br>HLA-DRB3*01:01<br>HLA-DRB3*02:02<br>HLA-DRB4*01:01<br>HLA-DRB5*01:01<br>HLA-DQA1*05:01/DQB1*02:01<br>HLA-DQA1*05:01/DQB1*03:01<br>HLA-DQA1*03:01/DQB1*03:02<br>HLA-DQA1*04:01/DQB1*04:02<br>HLA-DQA1*01:01/DQB1*05:01<br>HLA-DQA1*01:02/DQB1*06:02<br>HLA-DPA1*02:01/DPB1*01:01<br>HLA-DPA1*01:03/DPB1*02:01<br>HLA-DPA1*01:03/DPB1*04:01<br>HLA-DPA1*03:01/DPB1*04:02<br>HLA-DPA1*02:01/DPB1*05:01<br>HLA-DPA1*02:01/DPB1*14:01

TABLE III

Overlap technique between MHCI/CTL and MHCII/HTL complexes with B-cells for each of the proteins used in the work

MHC I					
	IEDB vs NetCTL	MHC I vs Cel B	Class I immunogenicity for MHC I > 0.1	Removal of up to 2 AA	Vaxijen (cut off 0.7)
WP_012148219.1	86	70	17	15	4
WP_012148227.1	48	36	11	7	2
WP_012148552.1	30	16	1	1	1
WP_012148811.1	35	21	3	3	1
WP_012148819.1	57	50	16	10	2
WP_012149174.1	45	39	7	6	3
WP_012149175.1	37	23	6	5	0
WP_012149185.1	42	21	5	5	0
Total	380	276	66	52	13
MHC II					
	IEDB vs NetMHCII	MHC II vs Cel B	Removal of up to 2 AA	Vaxijen (cut off 0.7)	
WP_012148219.1	58	15	10	3	
WP_012148227.1	51	9	5	1	
WP_012148552.1	42	4	3	2	
WP_012148811.1	17	1	1	0	
WP_012148819.1	54	6	5	1	
WP_012149174.1	50	15	10	4	
WP_012149175.1	33	7	5	2	
WP_012149185.1	23	2	2	0	
Total	328	59	41	13	

MHC I: histocompatibility complex molecules type 1; CTL: cytotoxic T lymphocytes; MHC II: histocompatibility complex molecules type 2; HTL: T helper lymphocytes; IEDB: the Immune Epitope Database.

TABLE IV

Prediction of discontinued epitopes of B cells

Epitopes	Residues	Number of residues	Scores
1	A:H349, A:T350, A:K351	3	0.929
2	A:R449, A:G450, A:T451, A:V452, A:G453, A:L454, A:A455, A:A456, A:Y457	9	0.849
3	A:L525, A:K526, A:A527, A:A528, A:Y529, A:T530, A:G531, A:Q532, A:F533, A:H534	10	0.828
4	A:L272, A:G273, A:T274, A:S275, A:F276, A:E277, A:V278, A:A279, A:Q280, A:G281, A:V282, A:G283, A:P284, A:G285, A:P286, A:G287, A:M288, A:A289, A:L290	19	0.824
5	A:S138, A:L139, A:G140, A:T141, A:S142, A:G143, A:P144, A:G145, A:P146, A:G147, A:A148, A:T149, A:S150, A:V151, A:K152	15	0.821
6	A:Y297, A:P299, A:L300, A:F301, A:S302, A:G303, A:P304, A:G305, A:P306, A:G307, A:N308, A:S309, A:D310, A:T311, A:S312, A:I313, A:K314	17	0.808
7	A:L153, A:K154, A:S155, A:N156, A:M157, A:T158, A:V159, A:S160, A:V161, A:D162, A:G163, A:P164, A:G165, A:P166, A:G167, A:P168, A:K169, A:L170, A:E171, A:Y172, A:K173, A:P174, A:N175, A:L176, A:V177, A:G178, A:N179, A:K180	28	0.762
8	A:E507, A:C508, A:G509, A:E510, A:V511, A:N512, A:P513, A:I514	8	0.737
9	A:G338, A:T339, A:S340, A:A341, A:Q342, A:G343, A:P344, A:G345, A:P346, A:G347, A:I348, A:V358, A:E359, A:V360, A:L361, A:N362, A:G363, A:P364, A:G365, A:P366, A:G367, A:G368, A:K393	23	0.725
10	A:M352, A:D353, A:R354, A:T355, A:K356, A:G357	6	0.7

TABLE V

Prediction of stimulation of IFNepitope in each epitope of our construct

Número	Sequência/Epítopo	Método	Resultado	Score
1	GPGPGEYKIMPGLLP	SVM	POSITIVE	0.00081751901
2	AAAKMAENPNIDDLP	SVM	NEGATIVE	-0.0083222059
3	AAKMAENPNIDDLPA	SVM	NEGATIVE	-0.33371237
4	AKMAENPNIDDLPAP	SVM	NEGATIVE	-0.45271403
5	KMAENPNIDDLPAPL	SVM	NEGATIVE	-0.4840845
6	MAENPNIDDLPAPLL	SVM	NEGATIVE	-0.4134494
7	AENPNIDDLPAPLLA	SVM	NEGATIVE	-0.71224553
8	ENPNIDDLPAPLLAA	SVM	NEGATIVE	-0.45431911
9	NPNIDDLPAPLLAAL	SVM	NEGATIVE	-0.74891022
10	PNIDDLPAPLLAALG	SVM	NEGATIVE	-0.52187068
11	NIDDLPAPLLAALGA	SVM	NEGATIVE	-0.57934186
12	IDDLPAPLLAALGAA	SVM	NEGATIVE	-0.6699177
13	DDLPAPLLAALGAAD	SVM	NEGATIVE	-0.72351262
14	DLPAPLLAALGAADL	SVM	NEGATIVE	-0.4164655
15	LPAPLLAALGAADLA	SVM	NEGATIVE	-0.29926524
16	PAPLLAALGAADLAL	SVM	NEGATIVE	-0.19584417
17	ELTQEALGTVASQTR	SVM	POSITIVE	0.0053471244
18	GYLEAATNRYNELVE	SVM	POSITIVE	0.0099973701
19	EGYVDQAVELTQEAL	SVM	POSITIVE	0.013816801
20	YNELVERGEAALQRL	SVM	POSITIVE	0.015511647
21	AYNECGEVNPIAAYG	SVM	POSITIVE	0.018395856
22	IELRDKFTTEELRKA	SVM	POSITIVE	0.019814547
23	VDQAVELTQEALGTV	SVM	POSITIVE	0.021024276
24	FIELRDKFTTEELRK	SVM	POSITIVE	0.022702707
25	TRGTVGLAAYSSFKI	SVM	POSITIVE	0.025585894
26	NECGEVNPIAAYGTV	SVM	POSITIVE	0.026568109
27	FIAAAYGQIGVDFYA	SVM	POSITIVE	0.030906254
28	LALATVNDLIANLRE	SVM	NEGATIVE	-0.033176546
29	ALATVNDLIANLRER	SVM	NEGATIVE	-0.22745613
30	LATVNDLIANLRERA	SVM	NEGATIVE	-0.047548487
31	FLLPYIFIAAAYGQI	SVM	POSITIVE	0.032212977
32	TVNDLIANLRERAEE	SVM	NEGATIVE	-0.16534372
33	VNDLIANLRERAEET	SVM	NEGATIVE	-0.65707349
34	NDLIANLRERAEETR	SVM	NEGATIVE	-0.59219489
35	VAAYRVNFFTPKMAA	SVM	POSITIVE	0.035810386
36	AYGTVINVHLKAAYT	SVM	POSITIVE	0.041909028
37	AAYKTTRGTVGLAAY	SVM	POSITIVE	0.043361796
38	SQTAFEDASARAEGY	SVM	POSITIVE	0.044101115

39	NRYNELVERGEAALQ	SVM	POSITIVE	0.046841932
40	GEAALQRLRSQTAFE	SVM	POSITIVE	0.047961904
41	EDASARAEGYVDQAV	SVM	POSITIVE	0.051516293
42	VGNKTVGPGPGNANG	SVM	POSITIVE	0.055051808
43	LTLGTSAQGPGPGIH	SVM	POSITIVE	0.055145257
44	SIKLEGFYLLEGPGP	SVM	POSITIVE	0.060315712
45	RGEAALQRLRSQTAF	SVM	POSITIVE	0.060445107
46	QVTAGTGGWAAYTAN	SVM	POSITIVE	0.062536902
47	TLTVNNPSLTGPGPG	SVM	POSITIVE	0.068458746
48	AAYTANTGGNRYAAY	SVM	POSITIVE	0.071460395
49	AETRTRVEERRARLT	SVM	NEGATIVE	-0.070375625
50	ETRTRVEERRARLTK	SVM	NEGATIVE	-0.26865463
51	TRTRVEERRARLTKF	SVM	NEGATIVE	-0.1707879
52	RTRVEERRARLTKFQ	SVM	NEGATIVE	-0.33710604
53	TRVEERRARLTKFQE	SVM	NEGATIVE	-0.5377821
54	RVEERRARLTKFQED	SVM	NEGATIVE	-0.5641927
55	VEERRARLTKFQEDL	SVM	NEGATIVE	-0.6915817
56	EERRARLTKFQEDLP	SVM	NEGATIVE	-0.86347699
57	ERRARLTKFQEDLPE	SVM	NEGATIVE	-0.81750768
58	RRARLTKFQEDLPEQ	SVM	NEGATIVE	-0.70032566
59	RARLTKFQEDLPEQF	SVM	NEGATIVE	-0.75787356
60	ARLTKFQEDLPEQFI	SVM	NEGATIVE	-0.76045003
61	RLTKFQEDLPEQFIE	SVM	NEGATIVE	-0.79239767
62	LTKFQEDLPEQFIEL	SVM	NEGATIVE	-0.62274853
63	TKFQEDLPEQFIELR	SVM	NEGATIVE	-0.50992006
64	KFQEDLPEQFIELRD	SVM	NEGATIVE	-0.43037405
65	FQEDLPEQFIELRDK	SVM	NEGATIVE	-0.46237585
66	QEDLPEQFIELRDKF	SVM	NEGATIVE	-0.54791675
67	EDLPEQFIELRDKFT	SVM	NEGATIVE	-0.63503871
68	DLPEQFIELRDKFTT	SVM	NEGATIVE	-0.39834432
69	LPEQFIELRDKFTTE	SVM	NEGATIVE	-0.39696285
70	PEQFIELRDKFTTEE	SVM	NEGATIVE	-0.57994486
71	EQFIELRDKFTTEEL	SVM	NEGATIVE	-0.17647669
72	QFIELRDKFTTEELR	SVM	NEGATIVE	-0.030704287
73	AAYKSTRGNIVKAAY	SVM	POSITIVE	0.071611421
74	CGEVNPIAAYGTVIN	SVM	POSITIVE	0.072554322
75	TRGNIVKAAYKTTRG	SVM	POSITIVE	0.075834649
76	LRDKFTTEELRKAAE	SVM	NEGATIVE	-0.097349091
77	RDKFTTEELRKAAEG	SVM	NEGATIVE	-0.082332581
78	DKFTTEELRKAAEGY	SVM	NEGATIVE	-0.040332333
79	KFTTEELRKAAEGYL	SVM	NEGATIVE	-0.094277929

80	FTTEELRKAAEGYLE	SVM	NEGATIVE	-0.017476923
81	PSLTGPGPGMENQWY	SVM	POSITIVE	0.075968726
82	ATVNDLIANLRERAE	SVM	POSITIVE	0.076253348
83	AADLALATVNDLIAN	SVM	POSITIVE	0.076778464
84	VNPIAAYGTVINVHL	SVM	POSITIVE	0.078877951
85	LVGNKTVGPGPGNAN	SVM	POSITIVE	0.08247417
86	PGNSDTSIKLEGFYL	SVM	POSITIVE	0.082718777
87	SAQGPGPGIHTKMD	SVM	POSITIVE	0.085461753
88	ARAEGYVDQAVELTQ	SVM	POSITIVE	0.085530564
89	AAYRVNFFTPKMAAY	SVM	POSITIVE	0.089416816
90	IAAYGTVINVHLKAA	SVM	POSITIVE	0.094507614
91	PNLVGNKTVGPGPGN	SVM	POSITIVE	0.095238414
92	YLEAATNRYNELVER	SVM	NEGATIVE	-0.055192107
93	PIAAYGTVINVHLKA	SVM	POSITIVE	0.095878676
94	ELRDKFTTEELRKAA	SVM	POSITIVE	0.096391677
95	AYGQIGVDFYAAAYN	SVM	POSITIVE	0.096642774
96	TNISYKLTLGTSAQG	SVM	POSITIVE	0.097038842
97	AAYFLLPYIFIAAAY	SVM	POSITIVE	0.097397326
98	RYNELVERGEAALQR	SVM	POSITIVE	0.099048937
99	VNNPSLTGPGPGMEN	SVM	POSITIVE	0.10153799
100	TNRYNELVERGEAAL	SVM	POSITIVE	0.10441409
101	NELVERGEAALQRLR	SVM	NEGATIVE	-0.064146658
102	ELVERGEAALQRLRS	SVM	NEGATIVE	-0.31591531
103	LVERGEAALQRLRSQ	SVM	NEGATIVE	-0.20848673
104	VERGEAALQRLRSQT	SVM	NEGATIVE	-0.10304081
105	ERGEAALQRLRSQTA	SVM	NEGATIVE	-0.07642814
106	YAAYKSTRGNIVKAA	SVM	POSITIVE	0.11124026
107	YLLEGPGPGRTNISY	SVM	POSITIVE	0.11252606
108	EAALQRLRSQTAFED	SVM	NEGATIVE	-0.079015532
109	AALQRLRSQTAFEDA	SVM	NEGATIVE	-0.15268613
110	ALQRLRSQTAFEDAS	SVM	NEGATIVE	-0.34101947
111	LQRLRSQTAFEDASA	SVM	NEGATIVE	-0.36158074
112	QRLRSQTAFEDASAR	SVM	NEGATIVE	-0.21286094
113	RLRSQTAFEDASARA	SVM	NEGATIVE	-0.20318048
114	LRSQTAFEDASARAE	SVM	NEGATIVE	-0.091971595
115	RSQTAFEDASARAEG	SVM	NEGATIVE	-0.048411334
116	EGFYLLEGPGPGRTN	SVM	POSITIVE	0.11729918
117	QTAFEDASARAEGYV	SVM	NEGATIVE	-0.045260359
118	TAFEDASARAEGYVD	SVM	NEGATIVE	-0.054936166
119	AFEDASARAEGYVDQ	SVM	NEGATIVE	-0.154556
120	FEDASARAEGYVDQA	SVM	NEGATIVE	-0.061549755

121	DLIANLRERAEETRA	SVM	POSITIVE	0.12148915
122	IKLEGFYLLEGPGPG	SVM	POSITIVE	0.12402411
123	YAAAYNECGEVNPIA	SVM	POSITIVE	0.12723401
124	TTRGTVGLAAYSSFK	SVM	POSITIVE	0.12761045
125	AYKSTRGNIVKAAYK	SVM	POSITIVE	0.13281497
126	TMIGPGPGEYKIMPG	SVM	POSITIVE	0.13458411
127	AEGYVDQAVELTQEA	SVM	NEGATIVE	-0.039107974
128	AGTGGWAAYTANTGG	SVM	POSITIVE	0.13658353
129	GYVDQAVELTQEALG	SVM	NEGATIVE	-0.11955742
130	YVDQAVELTQEALGT	SVM	NEGATIVE	-0.11440732
131	RGTVGLAAYSSFKIT	SVM	POSITIVE	0.14007839
132	DQAVELTQEALGTVA	SVM	NEGATIVE	-0.084015188
133	QAVELTQEALGTVAS	SVM	NEGATIVE	-0.15625201
134	AVELTQEALGTVASQ	SVM	NEGATIVE	-0.092045326
135	VELTQEALGTVASQT	SVM	NEGATIVE	-0.059555371
136	ADLALATVNDLIANL	SVM	POSITIVE	0.14128778
137	TGQFHIAPY	SVM	POSITIVE	0.14189666
138	MIGPGPGEYKIMPGL	SVM	POSITIVE	0.14258261
139	LEAATNRYNELVERG	SVM	POSITIVE	0.14280402
140	RYAAYKSTRGNIVKA	SVM	POSITIVE	0.14750068
141	KLEGFYLLEGPGPGR	SVM	POSITIVE	0.14849352
142	GPGPGNSDTSIKLEG	SVM	POSITIVE	0.14960645
143	AAAYNECGEVNPIAA	SVM	POSITIVE	0.14968424
144	GRTNISYKLTLGTSA	SVM	POSITIVE	0.15096884
145	PGPGIHTKMD	SVM	POSITIVE	0.15930109
146	YQVTAGTGGWAAYTA	SVM	POSITIVE	0.16270366
147	AAYQVTAGTGGWAAY	SVM	POSITIVE	0.16511064
148	EAATNRYNELVERGE	SVM	POSITIVE	0.17927253
149	AATNRYNELVERGEA	SVM	POSITIVE	0.17927253
150	ATNRYNELVERGEAA	SVM	POSITIVE	0.17927253
151	TLGTSAQGPGPGIHT	SVM	POSITIVE	0.18080032
152	NTGGNRYAAYKSTRG	SVM	POSITIVE	0.18580132
153	GERAAKLVGIELKNK	SVM	NEGATIVE	-0.12147046
154	ERAAKLVGIELKNKI	SVM	NEGATIVE	-0.41396249
155	RAAKLVGIELKNKIN	SVM	NEGATIVE	-0.19259786
156	AAKLVGIELKNKINF	SVM	NEGATIVE	-0.1915538
157	AKLVGIELKNKINFA	SVM	NEGATIVE	-0.31868965
158	KLVGIELKNKINFAY	SVM	NEGATIVE	-0.34470609
159	LVGIELKNKINFAYQ	SVM	NEGATIVE	-0.12721729
160	VGIELKNKINFAYQL	SVM	NEGATIVE	-0.11427754
161	GIELKNKINFAYQLS	SVM	NEGATIVE	-0.20831596

162	IELKNKINFAYQLSL	SVM	NEGATIVE	-0.3869098
163	ELKNKINFAYQLSLG	SVM	NEGATIVE	-0.31783601
164	LKNKINFAYQLSLGT	SVM	NEGATIVE	-0.52445227
165	KNKINFAYQLSLGTS	SVM	NEGATIVE	-0.55575013
166	NKINFAYQLSLGTSG	SVM	NEGATIVE	-0.63870886
167	KINFAYQLSLGTSGP	SVM	NEGATIVE	-0.95307898
168	INFAYQLSLGTSGPG	SVM	NEGATIVE	-10.184.834
169	NFAYQLSLGTSGPGP	SVM	NEGATIVE	-0.97514565
170	FAYQLSLGTSGPGPG	SVM	NEGATIVE	-0.7746566
171	AYQLSLGTSGPGPGA	SVM	NEGATIVE	-0.76924683
172	YQLSLGTSGPGPGAT	SVM	NEGATIVE	-0.90028228
173	QLSLGTSGPGPGATS	SVM	NEGATIVE	-0.89839153
174	LSLGTSGPGPGATSV	SVM	NEGATIVE	-0.85513829
175	SLGTSGPGPGATSVK	SVM	NEGATIVE	-0.6978686
176	LGTSGPGPGATSVKL	SVM	NEGATIVE	-0.76675732
177	GTSGPGPGATSVKLK	SVM	NEGATIVE	-0.82693225
178	TSGPGPGATSVKLKS	SVM	NEGATIVE	-0.81012001
179	SGPGPGATSVKLKSN	SVM	NEGATIVE	-0.92714853
180	GPGPGATSVKLKSNM	SVM	NEGATIVE	-0.98571887
181	PGPGATSVKLKSNMT	SVM	NEGATIVE	-0.8708089
182	GPGATSVKLKSNMTV	SVM	NEGATIVE	-12.871.509
183	PGATSVKLKSNMTVS	SVM	NEGATIVE	-0.87389666
184	GATSVKLKSNMTVSV	SVM	NEGATIVE	-0.76196648
185	ATSVKLKSNMTVSVD	SVM	NEGATIVE	-0.61868005
186	TSVKLKSNMTVSVDG	SVM	NEGATIVE	-0.79884227
187	SVKLKSNMTVSVDGP	SVM	NEGATIVE	-10.118.588
188	VKLKSNMTVSVDGPG	SVM	NEGATIVE	-11.670.819
189	KLKSNMTVSVDGPGP	SVM	NEGATIVE	-10.669.956
190	LKSNMTVSVDGPGPG	SVM	NEGATIVE	-0.85366904
191	KSNMTVSVDGPGPGP	SVM	NEGATIVE	-0.71678664
192	SNMTVSVDGPGPGPK	SVM	NEGATIVE	-0.79266066
193	NMTVSVDGPGPGPKL	SVM	NEGATIVE	-0.76674984
194	MTVSVDGPGPGPKLE	SVM	NEGATIVE	-0.53222368
195	TVSVDGPGPGPKLEY	SVM	NEGATIVE	-0.43960322
196	VSVDGPGPGPKLEYK	SVM	NEGATIVE	-0.51418992
197	SVDGPGPGPKLEYKP	SVM	NEGATIVE	-0.52287797
198	VDGPGPGPKLEYKPN	SVM	NEGATIVE	-0.5172335
199	DGPGPGPKLEYKPNL	SVM	NEGATIVE	-0.53581897
200	GPGPGPKLEYKPNLV	SVM	NEGATIVE	-0.60970824
201	PGPGPKLEYKPNLVG	SVM	NEGATIVE	-0.60764339
202	GPGPKLEYKPNLVGN	SVM	NEGATIVE	-0.75549418

203	PGPKLEYKPNLVGNK	SVM	NEGATIVE	-0.72348396
204	GPKLEYKPNLVGNKT	SVM	NEGATIVE	-10.337.245
205	PKLEYKPNLVGNKTV	SVM	NEGATIVE	-0.90038399
206	KLEYKPNLVGNKTVG	SVM	NEGATIVE	-0.41959291
207	LEYKPNLVGNKTVGP	SVM	NEGATIVE	-0.62741765
208	EYKPNLVGNKTVGPG	SVM	NEGATIVE	-0.50356374
209	YKPNLVGNKTVGPGP	SVM	NEGATIVE	-0.77810594
210	KPNLVGNKTVGPGPG	SVM	NEGATIVE	-0.32285321
211	EGYLEAATNRYNELV	SVM	POSITIVE	0.18595309
212	NLVGNKTVGPGPGNA	SVM	NEGATIVE	-0.00077354888
213	AALGAADLALATVND	SVM	POSITIVE	0.19038392
214	WAAYTANTGGNRYAA	SVM	POSITIVE	0.19286017
215	GNKTVGPGPGNANGI	SVM	NEGATIVE	-0.11467578
216	NKTVGPGPGNANGIT	SVM	NEGATIVE	-0.46074431
217	KTVGPGPGNANGITL	SVM	NEGATIVE	-0.45846355
218	TVGPGPGNANGITLT	SVM	NEGATIVE	-0.21058253
219	VGPGPGNANGITLTV	SVM	NEGATIVE	-0.21058253
220	GPGPGNANGITLTVN	SVM	NEGATIVE	-0.3519491
221	PGPGNANGITLTVNN	SVM	NEGATIVE	-0.25116477
222	GPGNANGITLTVNNP	SVM	NEGATIVE	-0.44479488
223	PGNANGITLTVNNPS	SVM	NEGATIVE	-0.28321988
224	GNANGITLTVNNPSL	SVM	NEGATIVE	-0.5739404
225	NANGITLTVNNPSLT	SVM	NEGATIVE	-0.48927408
226	ANGITLTVNNPSLTG	SVM	NEGATIVE	-0.57787533
227	NGITLTVNNPSLTGP	SVM	NEGATIVE	-0.61248747
228	GITLTVNNPSLTGPG	SVM	NEGATIVE	-0.25126042
229	ITLTVNNPSLTGPGP	SVM	NEGATIVE	-0.37543856
230	SARAEGYVDQAVELT	SVM	POSITIVE	0.19607458
231	EVNPIAAYGTVINVH	SVM	POSITIVE	0.20688699
232	TVNNPSLTGPGPGME	SVM	NEGATIVE	-0.014119672
233	AAYGTVINVHLKAAY	SVM	POSITIVE	0.2098964
234	NNPSLTGPGPGMENQ	SVM	NEGATIVE	-0.0047159632
235	NPSLTGPGPGMENQW	SVM	NEGATIVE	-0.010134443
236	IGPGPGEYKIMPGLL	SVM	POSITIVE	0.21231044
237	SLTGPGPGMENQWYL	SVM	NEGATIVE	-0.12938235
238	LTGPGPGMENQWYLK	SVM	NEGATIVE	-0.18046298
239	TGPGPGMENQWYLKL	SVM	NEGATIVE	-0.38898278
240	GPGPGMENQWYLKLN	SVM	NEGATIVE	-0.18668857
241	PGPGMENQWYLKLNA	SVM	NEGATIVE	-0.14642392
242	GPGMENQWYLKLNAG	SVM	NEGATIVE	-0.34121278
243	PGMENQWYLKLNAGT	SVM	NEGATIVE	-0.17444882

244	GMENQWYLKLNAGTM	SVM	NEGATIVE	-0.064187547
245	MENQWYLKLNAGTMI	SVM	NEGATIVE	-0.04436069
246	ENQWYLKLNAGTMIG	SVM	NEGATIVE	-0.21880781
247	NQWYLKLNAGTMIGP	SVM	NEGATIVE	-0.63838174
248	QWYLKLNAGTMIGPG	SVM	NEGATIVE	-0.47000225
249	WYLKLNAGTMIGPGP	SVM	NEGATIVE	-0.62041533
250	YLKLNAGTMIGPGPG	SVM	NEGATIVE	-0.40561541
251	LKLNAGTMIGPGPGE	SVM	NEGATIVE	-0.39641993
252	KLNAGTMIGPGPGEY	SVM	NEGATIVE	-0.28128392
253	LNAGTMIGPGPGEYK	SVM	NEGATIVE	-0.35728398
254	NAGTMIGPGPGEYKI	SVM	NEGATIVE	-0.4823715
255	AGTMIGPGPGEYKIM	SVM	NEGATIVE	-0.26023432
256	GTMIGPGPGEYKIMP	SVM	NEGATIVE	-0.46243093
257	LTVNNPSLTGPGPGM	SVM	POSITIVE	0.21383011
258	SDTSIKLEGFYLLEG	SVM	POSITIVE	0.21573808
259	TGGWAAYTANTGGNR	SVM	POSITIVE	0.21950658
260	ECGEVNPIAAYGTVI	SVM	POSITIVE	0.22297771
261	PGPGEYKIMPGLLPY	SVM	NEGATIVE	-0.081710037
262	GPGEYKIMPGLLPYA	SVM	NEGATIVE	-0.45888939
263	PGEYKIMPGLLPYAE	SVM	NEGATIVE	-0.47504599
264	GEYKIMPGLLPYAEI	SVM	NEGATIVE	-0.81632467
265	EYKIMPGLLPYAEIS	SVM	NEGATIVE	-0.6842841
266	YKIMPGLLPYAEISG	SVM	NEGATIVE	-0.78555028
267	KIMPGLLPYAEISGP	SVM	NEGATIVE	-10.631.175
268	IMPGLLPYAEISGPG	SVM	NEGATIVE	-0.68605427
269	MPGLLPYAEISGPGP	SVM	NEGATIVE	-0.76998373
270	PGLLPYAEISGPGPG	SVM	NEGATIVE	-0.36433537
271	GLLPYAEISGPGPGS	SVM	NEGATIVE	-0.84522185
272	LLPYAEISGPGPGSK	SVM	NEGATIVE	-10.900.427
273	LPYAEISGPGPGSKM	SVM	NEGATIVE	-11.323.852
274	PYAEISGPGPGSKMV	SVM	NEGATIVE	-12.700.792
275	YAEISGPGPGSKMVK	SVM	NEGATIVE	-10.571.596
276	AEISGPGPGSKMVKV	SVM	NEGATIVE	-10.829.879
277	EISGPGPGSKMVKVD	SVM	NEGATIVE	-10.272.501
278	ISGPGPGSKMVKVDY	SVM	NEGATIVE	-10.183.881
279	SGPGPGSKMVKVDYP	SVM	NEGATIVE	-1.366.205
280	GPGPGSKMVKVDYPF	SVM	NEGATIVE	-10.820.981
281	PGPGSKMVKVDYPFL	SVM	NEGATIVE	-0.8275652
282	GPGSKMVKVDYPFLI	SVM	NEGATIVE	-0.92315445
283	PGSKMVKVDYPFLIA	SVM	NEGATIVE	-0.44153551
284	GSKMVKVDYPFLIAD	SVM	NEGATIVE	-0.47197913

285	SKMVKVDYPFLIADN	SVM	NEGATIVE	-0.37520278
286	KMVKVDYPFLIADNG	SVM	NEGATIVE	-0.36995935
287	MVKVDYPFLIADNGP	SVM	NEGATIVE	-0.58326539
288	VKVDYPFLIADNGPG	SVM	NEGATIVE	-0.37476913
289	KVDYPFLIADNGPGP	SVM	NEGATIVE	-0.53671927
290	VDYPFLIADNGPGPG	SVM	NEGATIVE	-0.20747583
291	DYPFLIADNGPGPGY	SVM	NEGATIVE	-0.11816894
292	YPFLIADNGPGPGYQ	SVM	NEGATIVE	-0.087526193
293	PFLIADNGPGPGYQL	SVM	NEGATIVE	-0.053297345
294	FLIADNGPGPGYQLS	SVM	NEGATIVE	-0.39887687
295	LIADNGPGPGYQLSL	SVM	NEGATIVE	-0.52702483
296	IADNGPGPGYQLSLG	SVM	NEGATIVE	-0.72373305
297	ADNGPGPGYQLSLGT	SVM	NEGATIVE	-0.87996177
298	DNGPGPGYQLSLGTS	SVM	NEGATIVE	-0.91378502
299	NGPGPGYQLSLGTSF	SVM	NEGATIVE	-0.99592658
300	GPGPGYQLSLGTSFE	SVM	NEGATIVE	-0.83712817
301	PGPGYQLSLGTSFEV	SVM	NEGATIVE	-0.72884912
302	GPGYQLSLGTSFEVA	SVM	NEGATIVE	-0.86504631
303	PGYQLSLGTSFEVAQ	SVM	NEGATIVE	-0.43800422
304	GYQLSLGTSFEVAQG	SVM	NEGATIVE	-0.38446651
305	YQLSLGTSFEVAQGV	SVM	NEGATIVE	-0.55028793
306	QLSLGTSFEVAQGVG	SVM	NEGATIVE	-0.6796587
307	LSLGTSFEVAQGVGP	SVM	NEGATIVE	-0.75344882
308	SLGTSFEVAQGVGPG	SVM	NEGATIVE	-0.6253869
309	LGTSFEVAQGVGPGP	SVM	NEGATIVE	-0.70497141
310	GTSFEVAQGVGPGPG	SVM	NEGATIVE	-0.36510859
311	TSFEVAQGVGPGPGM	SVM	NEGATIVE	-0.33047011
312	SFEVAQGVGPGPGMA	SVM	NEGATIVE	-0.098965602
313	FEVAQGVGPGPGMAL	SVM	NEGATIVE	-0.11968773
314	EVAQGVGPGPGMALK	SVM	NEGATIVE	-0.14676456
315	VAQGVGPGPGMALKR	SVM	NEGATIVE	-0.10916247
316	AQGVGPGPGMALKRD	SVM	NEGATIVE	-0.081824753
317	QGVGPGPGMALKRDY	SVM	NEGATIVE	-0.0014119525
318	GVGPGPGMALKRDYP	SVM	NEGATIVE	-0.29558011
319	VGPGPGMALKRDYPK	SVM	NEGATIVE	-0.81385926
320	GPGPGMALKRDYPKY	SVM	NEGATIVE	-0.66087395
321	PGPGMALKRDYPKYY	SVM	NEGATIVE	-0.5418075
322	GPGMALKRDYPKYYP	SVM	NEGATIVE	-0.71435069
323	PGMALKRDYPKYYPL	SVM	NEGATIVE	-0.40607288
324	GMALKRDYPKYYPLF	SVM	NEGATIVE	-0.44461694
325	MALKRDYPKYYPLFS	SVM	NEGATIVE	-0.52293236

326	ALKRDYPKYYPLFSG	SVM	NEGATIVE	-0.60232406
327	LKRDYPKYYPLFSGP	SVM	NEGATIVE	-0.78760055
328	KRDYPKYYPLFSGPG	SVM	NEGATIVE	-0.7023448
329	RDYPKYYPLFSGPGP	SVM	NEGATIVE	-0.78468921
330	DYPKYYPLFSGPGPG	SVM	NEGATIVE	-0.42400323
331	YPKYYPLFSGPGPGN	SVM	NEGATIVE	-0.28829713
332	PKYYPLFSGPGPGNS	SVM	NEGATIVE	-0.37995864
333	KYYPLFSGPGPGNSD	SVM	NEGATIVE	-0.2351915
334	YYPLFSGPGPGNSDT	SVM	NEGATIVE	-0.26901535
335	YPLFSGPGPGNSDTS	SVM	NEGATIVE	-0.60199335
336	PLFSGPGPGNSDTSI	SVM	NEGATIVE	-0.37477089
337	LFSGPGPGNSDTSIK	SVM	NEGATIVE	-0.33132092
338	FSGPGPGNSDTSIKL	SVM	NEGATIVE	-0.21874709
339	SGPGPGNSDTSIKLE	SVM	NEGATIVE	-0.17702502
340	EAAAKMAENPNIDDL	SVM	POSITIVE	0.22408368
341	ALGAADLALATVNDL	SVM	POSITIVE	0.2269467
342	GPGNSDTSIKLEGFY	SVM	NEGATIVE	-0.1103031
343	TTEELRKAAEGYLEA	SVM	POSITIVE	0.23080213
344	GNSDTSIKLEGFYLL	SVM	NEGATIVE	-0.065420044
345	NSDTSIKLEGFYLLE	SVM	NEGATIVE	-0.023329883
346	DLALATVNDLIANLR	SVM	POSITIVE	0.23304405
347	DTSIKLEGFYLLEGP	SVM	NEGATIVE	-0.047076169
348	TSIKLEGFYLLEGPG	SVM	NEGATIVE	-0.028646032
349	GTGGWAAYTANTGGN	SVM	POSITIVE	0.23699595
350	VGERAAKLVGIELKN	SVM	POSITIVE	0.24884831
351	APLLAALGAADLALA	SVM	POSITIVE	0.25404264
352	GEVNPIAAYGTVINV	SVM	POSITIVE	0.25441553
353	RAEGYVDQAVELTQE	SVM	POSITIVE	0.25934691
354	GFYLLEGPGPGRTNI	SVM	NEGATIVE	-0.27713361
355	FYLLEGPGPGRTNIS	SVM	NEGATIVE	-0.11217144
356	AEGYLEAATNRYNEL	SVM	POSITIVE	0.26370284
357	LLEGPGPGRTNISYK	SVM	NEGATIVE	-0.046314171
358	LEGPGPGRTNISYKL	SVM	NEGATIVE	-0.1471395
359	EGPGPGRTNISYKLT	SVM	NEGATIVE	-0.1755268
360	GPGPGRTNISYKLTL	SVM	NEGATIVE	-0.20472778
361	PGPGRTNISYKLTLG	SVM	NEGATIVE	-0.029313913
362	GPGRTNISYKLTLGT	SVM	NEGATIVE	-0.30618056
363	PGRTNISYKLTLGTS	SVM	NEGATIVE	-0.13961339
364	RTNISYKLTLGTSAQ	SVM	POSITIVE	0.27056192
365	DASARAEGYVDQAVE	SVM	POSITIVE	0.27419673
366	GGWAAYTANTGGNRY	SVM	POSITIVE	0.27490477

367	NISYKLTLGTSAQGP	SVM	NEGATIVE	-0.13711911
368	ISYKLTLGTSAQGPG	SVM	NEGATIVE	-0.095125701
369	SYKLTLGTSAQGPGP	SVM	NEGATIVE	-0.45694781
370	YKLTLGTSAQGPGPG	SVM	NEGATIVE	-0.21092397
371	KLTLGTSAQGPGPGI	SVM	NEGATIVE	-0.036915205
372	IAAAYGQIGVDFYAA	SVM	POSITIVE	0.28221447
373	TQEALGTVASQTRAV	SVM	POSITIVE	0.28301464
374	LGTSAQGPGPGIHTK	SVM	NEGATIVE	-0.10368019
375	GTSAQGPGPGIHTKM	SVM	NEGATIVE	-0.090647472
376	TSAQGPGPGIHTKMD	SVM	NEGATIVE	-0.090959896
377	LGAADLALATVNDLI	SVM	POSITIVE	0.29475444
378	AQGPGPGIHTKMD	SVM	NEGATIVE	-0.024570467
379	QGPGPGIHTKMD	SVM	NEGATIVE	-0.013180579
380	GPGPGIHTKMD	SVM	NEGATIVE	-0.023156261
381	ASARAEGYVDQAVEL	SVM	POSITIVE	0.30587225
382	GPGIHTKMD	SVM	NEGATIVE	-0.14375157
383	RTKGVEVLNGPGPGG	SVM	NEGATIVE	-0.10823802
384	TKGVEVLNGPGPGGM	SVM	NEGATIVE	-0.29710325
385	KGVEVLNGPGPGGMH	SVM	NEGATIVE	-0.41909509
386	GVEVLNGPGPGGMHI	SVM	NEGATIVE	-0.26436672
387	VEVLNGPGPGGMHIM	SVM	NEGATIVE	-0.61374913
388	EVLNGPGPGGMHIML	SVM	NEGATIVE	-0.5410201
389	VLNGPGPGGMHIMLY	SVM	NEGATIVE	-0.5225017
390	LNGPGPGGMHIMLYD	SVM	NEGATIVE	-0.68046189
391	NGPGPGGMHIMLYDL	SVM	NEGATIVE	-0.66511339
392	GPGPGGMHIMLYDLK	SVM	NEGATIVE	-0.67329397
393	PGPGGMHIMLYDLKG	SVM	NEGATIVE	-0.65010033
394	GPGGMHIMLYDLKGP	SVM	NEGATIVE	-10.222.806
395	PGGMHIMLYDLKGPL	SVM	NEGATIVE	-0.87208497
396	GGMHIMLYDLKGPLN	SVM	NEGATIVE	-0.9990277
397	GMHIMLYDLKGPLNV	SVM	NEGATIVE	-10.703.545
398	MHIMLYDLKGPLNVA	SVM	NEGATIVE	-10.634.026
399	HIMLYDLKGPLNVAA	SVM	NEGATIVE	-0.75410297
400	IMLYDLKGPLNVAAY	SVM	NEGATIVE	-0.78763233
401	MLYDLKGPLNVAAYR	SVM	NEGATIVE	-0.62631051
402	LYDLKGPLNVAAYRV	SVM	NEGATIVE	-0.5003443
403	YDLKGPLNVAAYRVN	SVM	NEGATIVE	-0.52141954
404	DLKGPLNVAAYRVNF	SVM	NEGATIVE	-0.4777662
405	LKGPLNVAAYRVNFF	SVM	NEGATIVE	-0.33148922
406	KGPLNVAAYRVNFFT	SVM	NEGATIVE	-0.37214865
407	GPLNVAAYRVNFFTP	SVM	NEGATIVE	-0.37969539

408	PLNVAAYRVNFFTPK	SVM	NEGATIVE	-0.12716089
409	LNVAAYRVNFFTPKM	SVM	NEGATIVE	-0.21902906
410	NVAAYRVNFFTPKMA	SVM	NEGATIVE	-0.079865622
411	LTQEALGTVASQTRA	SVM	POSITIVE	0.30595246
412	IANLRERAEETRAET	SVM	POSITIVE	0.30682484
413	AYRVNFFTPKMAAYS	SVM	NEGATIVE	-0.10422298
414	YRVNFFTPKMAAYSS	SVM	NEGATIVE	-0.3775773
415	RVNFFTPKMAAYSSM	SVM	NEGATIVE	-0.67444568
416	VNFFTPKMAAYSSME	SVM	NEGATIVE	-0.66881444
417	NFFTPKMAAYSSMEN	SVM	NEGATIVE	-0.51183719
418	FFTPKMAAYSSMENQ	SVM	NEGATIVE	-0.66179478
419	FTPKMAAYSSMENQW	SVM	NEGATIVE	-0.68141827
420	TPKMAAYSSMENQWY	SVM	NEGATIVE	-0.56608184
421	PKMAAYSSMENQWYL	SVM	NEGATIVE	-0.62227546
422	KMAAYSSMENQWYLA	SVM	NEGATIVE	-0.53475378
423	MAAYSSMENQWYLAA	SVM	NEGATIVE	-0.34911677
424	AAYSSMENQWYLAAY	SVM	NEGATIVE	-0.26365417
425	AYSSMENQWYLAAYQ	SVM	NEGATIVE	-0.15746494
426	YSSMENQWYLAAYQV	SVM	NEGATIVE	-0.33498103
427	SSMENQWYLAAYQVT	SVM	NEGATIVE	-0.38314134
428	SMENQWYLAAYQVTA	SVM	NEGATIVE	-0.34487992
429	MENQWYLAAYQVTAG	SVM	NEGATIVE	-0.30993475
430	ENQWYLAAYQVTAGT	SVM	NEGATIVE	-0.48385965
431	NQWYLAAYQVTAGTG	SVM	NEGATIVE	-0.57966716
432	QWYLAAYQVTAGTGG	SVM	NEGATIVE	-0.51852304
433	WYLAAYQVTAGTGGW	SVM	NEGATIVE	-0.11832399
434	YLAAYQVTAGTGGWA	SVM	NEGATIVE	-0.043665071
435	LAAYQVTAGTGGWAA	SVM	NEGATIVE	-0.00016427105
436	ANLRERAEETRAETR	SVM	POSITIVE	0.30828533
437	AYQVTAGTGGWAAYT	SVM	NEGATIVE	-0.011873184
438	PGPGNSDTSIKLEGF	SVM	POSITIVE	0.3122424
439	GAADLALATVNDLIA	SVM	POSITIVE	0.31790981
440	VTAGTGGWAAYTANT	SVM	NEGATIVE	-0.025886878
441	GWAAYTANTGGNRYA	SVM	POSITIVE	0.31875773
442	NRYAAYKSTRGNIVK	SVM	POSITIVE	0.3275812
443	RKAAEGYLEAATNRY	SVM	POSITIVE	0.33922804
444	AAYGQIGVDFYAAAY	SVM	POSITIVE	0.34010472
445	LRKAAEGYLEAATNR	SVM	POSITIVE	0.34433339
446	RAETRTRVEERRARL	SVM	POSITIVE	0.34996506
447	LEGFYLLEGPGPGRT	SVM	POSITIVE	0.36412773
448	KAAEGYLEAATNRYN	SVM	POSITIVE	0.3645018

449	AYTANTGGNRYAAYK	SVM	NEGATIVE	-0.23441511
450	YTANTGGNRYAAYKS	SVM	NEGATIVE	-0.19718565
451	TANTGGNRYAAYKST	SVM	NEGATIVE	-0.13528146
452	ANTGGNRYAAYKSTR	SVM	NEGATIVE	-0.084446565
453	TAGTGGWAAYTANTG	SVM	POSITIVE	0.39957715
454	AAEGYLEAATNRYNE	SVM	POSITIVE	0.4059626
455	QEALGTVASQTRAVG	SVM	POSITIVE	0.40993056
456	NLRERAEETRAETRT	SVM	POSITIVE	0.41405318
457	ELRKAAEGYLEAATN	SVM	POSITIVE	0.42301994
458	EETRAETRTRVEERR	SVM	POSITIVE	0.43695956
459	LIANLRERAEETRAE	SVM	POSITIVE	0.45548391
460	EALGTVASQTRAVGE	SVM	POSITIVE	0.47919824
461	PLLAALGAADLALAT	SVM	POSITIVE	0.49926943
462	YKSTRGNIVKAAYKT	SVM	NEGATIVE	-0.21814903
463	KSTRGNIVKAAYKTT	SVM	NEGATIVE	-0.39225937
464	STRGNIVKAAYKTTR	SVM	NEGATIVE	-0.078681695
465	ALGTVASQTRAVGER	SVM	POSITIVE	0.52937284
466	RGNIVKAAYKTTRGT	SVM	NEGATIVE	-0.20089573
467	GNIVKAAYKTTRGTV	SVM	NEGATIVE	-0.56754019
468	NIVKAAYKTTRGTVG	SVM	NEGATIVE	-0.58871394
469	IVKAAYKTTRGTVGL	SVM	NEGATIVE	-0.37349172
470	VKAAYKTTRGTVGLA	SVM	NEGATIVE	-0.25098069
471	KAAYKTTRGTVGLAA	SVM	NEGATIVE	-0.17520884
472	EELRKAAEGYLEAAT	SVM	POSITIVE	0.55006559
473	AYKTTRGTVGLAAYS	SVM	NEGATIVE	-0.041432703
474	YKTTRGTVGLAAYSS	SVM	NEGATIVE	-0.2013011
475	KTTRGTVGLAAYSSF	SVM	NEGATIVE	-0.074321144
476	VASQTRAVGERAAKL	SVM	POSITIVE	0.55072089
477	TGGNRYAAYKSTRGN	SVM	POSITIVE	0.5532313
478	AEETRAETRTRVEER	SVM	POSITIVE	0.55920212
479	GTVGLAAYSSFKITF	SVM	NEGATIVE	-0.15417429
480	TVGLAAYSSFKITFF	SVM	NEGATIVE	-0.11339719
481	VGLAAYSSFKITFFF	SVM	NEGATIVE	-0.17431327
482	GLAAYSSFKITFFFA	SVM	NEGATIVE	-0.26243455
483	LAAYSSFKITFFFAA	SVM	NEGATIVE	-0.45249276
484	AAYSSFKITFFFAAY	SVM	NEGATIVE	-0.13233084
485	AYSSFKITFFFAAYP	SVM	NEGATIVE	-0.15977549
486	YSSFKITFFFAAYPY	SVM	NEGATIVE	-0.61950951
487	SSFKITFFFAAYPYI	SVM	NEGATIVE	-0.45366911
488	SFKITFFFAAYPYIF	SVM	NEGATIVE	-0.5448695
489	FKITFFFAAYPYIFI	SVM	NEGATIVE	-0.43000795

490	KITFFFAAYPYIFIA	SVM	NEGATIVE	-0.57581681
491	ITFFFAAYPYIFIAT	SVM	NEGATIVE	-0.63435883
492	TFFFAAYPYIFIATF	SVM	NEGATIVE	-0.65260825
493	FFFAAYPYIFIATFF	SVM	NEGATIVE	-0.50029468
494	FFAAYPYIFIATFFA	SVM	NEGATIVE	-0.46264627
495	FAAYPYIFIATFFAA	SVM	NEGATIVE	-0.47530544
496	AAYPYIFIATFFAAY	SVM	NEGATIVE	-0.51814797
497	AYPYIFIATFFAAYF	SVM	NEGATIVE	-0.68199196
498	YPYIFIATFFAAYFL	SVM	NEGATIVE	-0.89554833
499	PYIFIATFFAAYFLL	SVM	NEGATIVE	-0.63401709
500	YIFIATFFAAYFLLP	SVM	NEGATIVE	-0.38603493
501	IFIATFFAAYFLLPY	SVM	NEGATIVE	-0.59592354
502	FIATFFAAYFLLPYI	SVM	NEGATIVE	-0.42400306
503	IATFFAAYFLLPYIF	SVM	NEGATIVE	-0.68881184
504	ATFFAAYFLLPYIFI	SVM	NEGATIVE	-0.51799692
505	TFFAAYFLLPYIFIA	SVM	NEGATIVE	-0.68573776
506	FFAAYFLLPYIFIAA	SVM	NEGATIVE	-0.40326731
507	FAAYFLLPYIFIAAA	SVM	NEGATIVE	-0.018197573
508	TRAETRTRVEERRAR	SVM	POSITIVE	0.56407767
509	AYFLLPYIFIAAAYG	SVM	NEGATIVE	-0.016929804
510	YFLLPYIFIAAAYGQ	SVM	NEGATIVE	-0.22091168
511	AAAYGQIGVDFYAAA	SVM	POSITIVE	0.57637913
512	LLPYIFIAAAYGQIG	SVM	NEGATIVE	-0.15236759
513	LPYIFIAAAYGQIGV	SVM	NEGATIVE	-0.29150337
514	PYIFIAAAYGQIGVD	SVM	NEGATIVE	-0.31002772
515	YIFIAAAYGQIGVDF	SVM	NEGATIVE	-0.14951665
516	IFIAAAYGQIGVDFY	SVM	NEGATIVE	-0.32751191
517	TEELRKAAEGYLEAA	SVM	POSITIVE	0.58030736
518	TRAVGERAAKLVGIE	SVM	POSITIVE	0.62604005
519	ETRAETRTRVEERRA	SVM	POSITIVE	0.6328511
520	AVGERAAKLVGIELK	SVM	POSITIVE	0.63611803
521	GGNRYAAYKSTRGNI	SVM	POSITIVE	0.65128431
522	YGQIGVDFYAAAYNE	SVM	NEGATIVE	-0.062260931
523	GQIGVDFYAAAYNEC	SVM	NEGATIVE	-0.12662259
524	QIGVDFYAAAYNECG	SVM	NEGATIVE	-0.2272139
525	IGVDFYAAAYNECGE	SVM	NEGATIVE	-0.18350616
526	GVDFYAAAYNECGEV	SVM	NEGATIVE	-0.21775756
527	VDFYAAAYNECGEVN	SVM	NEGATIVE	-0.1407766
528	DFYAAAYNECGEVNP	SVM	NEGATIVE	-0.16003992
529	FYAAAYNECGEVNPI	SVM	NEGATIVE	-0.050204864
530	RERAEETRAETRTRV	SVM	POSITIVE	0.66965338

531	GNRYAAYKSTRGNIV	SVM	POSITIVE	0.67006343
532	AAYNECGEVNPIAAY	SVM	NEGATIVE	-0.028880976
533	ASQTRAVGERAAKLV	SVM	POSITIVE	0.67014771
534	YNECGEVNPIAAYGT	SVM	NEGATIVE	-0.14912259
535	TVASQTRAVGERAAK	SVM	POSITIVE	0.67359072
536	LAALGAADLALATVN	SVM	POSITIVE	0.6812668
537	LRERAEETRAETRTR	SVM	POSITIVE	0.6887185
538	SQTRAVGERAAKLVG	SVM	POSITIVE	0.70298562
539	ERAEETRAETRTRVE	SVM	POSITIVE	0.71554854
540	LLAALGAADLALATV	SVM	POSITIVE	0.75124621
541	NPIAAYGTVINVHLK	SVM	NEGATIVE	-0.005414537
542	GTVASQTRAVGERAA	SVM	POSITIVE	0.77149353
543	QTRAVGERAAKLVGI	SVM	POSITIVE	0.7913839
544	RAVGERAAKLVGIEL	SVM	POSITIVE	0.86574214
545	RAEETRAETRTRVEE	SVM	POSITIVE	0.86835363
546	YGTVINVHLKAAYTG	SVM	NEGATIVE	-0.25823997
547	GTVINVHLKAAYTGQ	SVM	NEGATIVE	-0.49112193
548	TVINVHLKAAYTGQF	SVM	NEGATIVE	-0.39419775
549	VINVHLKAAYTGQFH	SVM	NEGATIVE	-0.19730602
550	INVHLKAAYTGQFHI	SVM	NEGATIVE	-0.58936486
551	NVHLKAAYTGQFHIA	SVM	NEGATIVE	-0.55483486
552	VHLKAAYTGQFHIAP	SVM	NEGATIVE	-0.61972469
553	HLKAAYTGQFHIAPY	SVM	NEGATIVE	-0.74858747
554	LKAAYTGQFHIAPY	SVM	NEGATIVE	-0.6444112
555	KAAYTGQFHIAPY	SVM	NEGATIVE	-0.66480221
556	AAYTGQFHIAPY	SVM	NEGATIVE	-0.59809789
557	AYTGQFHIAPY	SVM	NEGATIVE	-0.44073237
558	YTGQFHIAPY	SVM	NEGATIVE	-0.2689636
559	LGTVASQTRAVGERA	SVM	POSITIVE	0.88559246

TABLE VI

Prediction of stimulation of IL6pred in each epitope of our construct

Start	Sequence	Score	Prediction
1	KNKINFAYQLSLGTS	0.02	IL-6 non-inducer
2	NKINFAYQLSLGTSG	0.02	IL-6 non-inducer
3	KINFAYQLSLGTSGP	0.02	IL-6 non-inducer
4	INFAYQLSLGTSGPG	0.02	IL-6 non-inducer
5	NFAYQLSLGTSGPGP	0.05	IL-6 non-inducer
6	FAYQLSLGTSGPGPG	0.03	IL-6 non-inducer
7	AYQLSLGTSGPGPGA	0.05	IL-6 non-inducer
8	YQLSLGTSGPGPGAT	0.05	IL-6 non-inducer
9	QLSLGTSGPGPGATS	0.04	IL-6 non-inducer
10	LSLGTSGPGPGATSV	0.05	IL-6 non-inducer
11	SLGTSGPGPGATSVK	0.02	IL-6 non-inducer
12	LGTSGPGPGATSVKL	0.02	IL-6 non-inducer
13	GTSGPGPGATSVKLK	0.01	IL-6 non-inducer
14	TSGPGPGATSVKLKS	0.0	IL-6 non-inducer
15	SGPGPGATSVKLKSN	0.01	IL-6 non-inducer
16	GPGPGATSVKLKSNM	0.01	IL-6 non-inducer
17	PGPGATSVKLKSNMT	0.01	IL-6 non-inducer
18	GPGATSVKLKSNMTV	0.03	IL-6 non-inducer
19	PGATSVKLKSNMTVS	0.02	IL-6 non-inducer
20	GATSVKLKSNMTVSV	0.02	IL-6 non-inducer
21	ATSVKLKSNMTVSVD	0.02	IL-6 non-inducer
22	TSVKLKSNMTVSVDG	0.01	IL-6 non-inducer
23	SVKLKSNMTVSVDGP	0.01	IL-6 non-inducer
24	VKLKSNMTVSVDGPG	0.01	IL-6 non-inducer
25	KLKSNMTVSVDGPGP	0.01	IL-6 non-inducer
26	LKSNMTVSVDGPGPG	0.01	IL-6 non-inducer
27	KSNMTVSVDGPGPGP	0.0	IL-6 non-inducer
28	SNMTVSVDGPGPGPK	0.01	IL-6 non-inducer
29	NMTVSVDGPGPGPKL	0.01	IL-6 non-inducer
30	MTVSVDGPGPGPKLE	0.01	IL-6 non-inducer
31	TVSVDGPGPGPKLEY	0.01	IL-6 non-inducer
32	VSVDGPGPGPKLEYK	0.03	IL-6 non-inducer
33	SVDGPGPGPKLEYKP	0.01	IL-6 non-inducer
34	VDGPGPGPKLEYKPN	0.01	IL-6 non-inducer
35	DGPGPGPKLEYKPNL	0.02	IL-6 non-inducer
36	GPGPGPKLEYKPNLV	0.01	IL-6 non-inducer
37	PGPGPKLEYKPNLVG	0.01	IL-6 non-inducer
38	GPGPKLEYKPNLVGN	0.03	IL-6 non-inducer

39	PGPKLEYKPNLVGNK	0.05	IL-6 non-inducer
40	GPKLEYKPNLVGNKT	0.07	IL-6 non-inducer
41	PKLEYKPNLVGNKTV	0.13	IL-6 inducer
42	KLEYKPNLVGNKTVG	0.04	IL-6 non-inducer
43	LEYKPNLVGNKTVGP	0.03	IL-6 non-inducer
44	EYKPNLVGNKTVGPG	0.04	IL-6 non-inducer
45	YKPNLVGNKTVGPGP	0.01	IL-6 non-inducer
46	KPNLVGNKTVGPGPG	0.02	IL-6 non-inducer
47	PNLVGNKTVGPGPGN	0.03	IL-6 non-inducer
48	NLVGNKTVGPGPGNA	0.03	IL-6 non-inducer
49	LVGNKTVGPGPGNAN	0.03	IL-6 non-inducer
50	VGNKTVGPGPGNANG	0.04	IL-6 non-inducer
51	GNKTVGPGPGNANGI	0.04	IL-6 non-inducer
52	NKTVGPGPGNANGIT	0.03	IL-6 non-inducer
53	KTVGPGPGNANGITL	0.01	IL-6 non-inducer
54	TVGPGPGNANGITLT	0.01	IL-6 non-inducer
55	VGPGPGNANGITLTV	0.01	IL-6 non-inducer
56	GPGPGNANGITLTVN	0.03	IL-6 non-inducer
57	PGPGNANGITLTVNN	0.04	IL-6 non-inducer
58	GPGNANGITLTVNNP	0.04	IL-6 non-inducer
59	PGNANGITLTVNNPS	0.02	IL-6 non-inducer
60	GNANGITLTVNNPSL	0.04	IL-6 non-inducer
61	NANGITLTVNNPSLT	0.03	IL-6 non-inducer
62	ANGITLTVNNPSLTG	0.05	IL-6 non-inducer
63	NGITLTVNNPSLTGP	0.04	IL-6 non-inducer
64	GITLTVNNPSLTGPG	0.03	IL-6 non-inducer
65	ITLTVNNPSLTGPGP	0.03	IL-6 non-inducer
66	TLTVNNPSLTGPGPG	0.03	IL-6 non-inducer
67	LTVNNPSLTGPGPGM	0.03	IL-6 non-inducer
68	TVNNPSLTGPGPGME	0.0	IL-6 non-inducer
69	VNNPSLTGPGPGMEN	0.01	IL-6 non-inducer
70	NNPSLTGPGPGMENQ	0.04	IL-6 non-inducer
71	NPSLTGPGPGMENQW	0.01	IL-6 non-inducer
72	PSLTGPGPGMENQWY	0.01	IL-6 non-inducer
73	SLTGPGPGMENQWYL	0.01	IL-6 non-inducer
74	LTGPGPGMENQWYLK	0.02	IL-6 non-inducer
75	TGPGPGMENQWYLKL	0.02	IL-6 non-inducer
76	GPGPGMENQWYLKLN	0.01	IL-6 non-inducer
77	PGPGMENQWYLKLNA	0.0	IL-6 non-inducer
78	GPGMENQWYLKLNAG	0.01	IL-6 non-inducer
79	PGMENQWYLKLNAGT	0.0	IL-6 non-inducer

80	GMENQWYLKLNAGTM	0.01	IL-6 non-inducer
81	MENQWYLKLNAGTMI	0.01	IL-6 non-inducer
82	ENQWYLKLNAGTMIG	0.01	IL-6 non-inducer
83	NQWYLKLNAGTMIGP	0.02	IL-6 non-inducer
84	QWYLKLNAGTMIGPG	0.01	IL-6 non-inducer
85	WYLKLNAGTMIGPGP	0.01	IL-6 non-inducer
86	YLKLNAGTMIGPGPG	0.03	IL-6 non-inducer
87	LKLNAGTMIGPGPGE	0.09	IL-6 non-inducer
88	KLNAGTMIGPGPGEY	0.0	IL-6 non-inducer
89	LNAGTMIGPGPGEYK	0.0	IL-6 non-inducer
90	NAGTMIGPGPGEYKI	0.01	IL-6 non-inducer
91	AGTMIGPGPGEYKIM	0.01	IL-6 non-inducer
92	GTMIGPGPGEYKIMP	0.01	IL-6 non-inducer
93	TMIGPGPGEYKIMPG	0.01	IL-6 non-inducer
94	MIGPGPGEYKIMPGL	0.0	IL-6 non-inducer
95	IGPGPGEYKIMPGLL	0.01	IL-6 non-inducer
96	GPGPGEYKIMPGLLP	0.02	IL-6 non-inducer
97	PGPGEYKIMPGLLPY	0.02	IL-6 non-inducer
98	GPGEYKIMPGLLPYA	0.01	IL-6 non-inducer
99	PGEYKIMPGLLPYAE	0.14	IL-6 inducer
100	GEYKIMPGLLPYAEI	0.15	IL-6 inducer
101	EYKIMPGLLPYAEIS	0.14	IL-6 inducer
102	YKIMPGLLPYAEISG	0.01	IL-6 non-inducer
103	KIMPGLLPYAEISGP	0.02	IL-6 non-inducer
104	IMPGLLPYAEISGPG	0.03	IL-6 non-inducer
105	MPGLLPYAEISGPGP	0.04	IL-6 non-inducer
106	PGLLPYAEISGPGPG	0.03	IL-6 non-inducer
107	GLLPYAEISGPGPGS	0.03	IL-6 non-inducer
108	LLPYAEISGPGPGSK	0.05	IL-6 non-inducer
109	LPYAEISGPGPGSKM	0.0	IL-6 non-inducer
110	PYAEISGPGPGSKMV	0.0	IL-6 non-inducer
111	YAEISGPGPGSKMVK	0.01	IL-6 non-inducer
112	AEISGPGPGSKMVKV	0.02	IL-6 non-inducer
113	EISGPGPGSKMVKVD	0.01	IL-6 non-inducer
114	ISGPGPGSKMVKVDY	0.01	IL-6 non-inducer
115	SGPGPGSKMVKVDYP	0.01	IL-6 non-inducer
116	GPGPGSKMVKVDYPF	0.02	IL-6 non-inducer
117	PGPGSKMVKVDYPFL	0.01	IL-6 non-inducer
118	GPGSKMVKVDYPFLI	0.01	IL-6 non-inducer
119	PGSKMVKVDYPFLIA	0.02	IL-6 non-inducer
120	GSKMVKVDYPFLIAD	0.05	IL-6 non-inducer

121	SKMVKVDYPFLIADN	0.01	IL-6 non-inducer
122	KMVKVDYPFLIADNG	0.0	IL-6 non-inducer
123	MVKVDYPFLIADNGP	0.04	IL-6 non-inducer
124	VKVDYPFLIADNGPG	0.03	IL-6 non-inducer
125	KVDYPFLIADNGPGP	0.02	IL-6 non-inducer
126	VDYPFLIADNGPGPG	0.03	IL-6 non-inducer
127	DYPFLIADNGPGPGY	0.01	IL-6 non-inducer
128	YPFLIADNGPGPGYQ	0.0	IL-6 non-inducer
129	PFLIADNGPGPGYQL	0.01	IL-6 non-inducer
130	FLIADNGPGPGYQLS	0.01	IL-6 non-inducer
131	LIADNGPGPGYQLSL	0.02	IL-6 non-inducer
132	IADNGPGPGYQLSLG	0.04	IL-6 non-inducer
133	ADNGPGPGYQLSLGT	0.05	IL-6 non-inducer
134	DNGPGPGYQLSLGTS	0.06	IL-6 non-inducer
135	NGPGPGYQLSLGTSF	0.06	IL-6 non-inducer
136	GPGPGYQLSLGTSFE	0.05	IL-6 non-inducer
137	PGPGYQLSLGTSFEV	0.03	IL-6 non-inducer
138	GPGYQLSLGTSFEVA	0.03	IL-6 non-inducer
139	PGYQLSLGTSFEVAQ	0.04	IL-6 non-inducer
140	GYQLSLGTSFEVAQG	0.05	IL-6 non-inducer
141	YQLSLGTSFEVAQGV	0.04	IL-6 non-inducer
142	QLSLGTSFEVAQGVG	0.07	IL-6 non-inducer
143	LSLGTSFEVAQGVGP	0.05	IL-6 non-inducer
144	SLGTSFEVAQGVGPG	0.03	IL-6 non-inducer
145	LGTSFEVAQGVGPGP	0.0	IL-6 non-inducer
146	GTSFEVAQGVGPGPG	0.04	IL-6 non-inducer
147	TSFEVAQGVGPGPGM	0.03	IL-6 non-inducer
148	SFEVAQGVGPGPGMA	0.03	IL-6 non-inducer
149	FEVAQGVGPGPGMAL	0.01	IL-6 non-inducer
150	EVAQGVGPGPGMALK	0.04	IL-6 non-inducer
151	VAQGVGPGPGMALKR	0.02	IL-6 non-inducer
152	AQGVGPGPGMALKRD	0.01	IL-6 non-inducer
153	QGVGPGPGMALKRDY	0.0	IL-6 non-inducer
154	GVGPGPGMALKRDYP	0.0	IL-6 non-inducer
155	VGPGPGMALKRDYPK	0.0	IL-6 non-inducer
156	GPGPGMALKRDYPKY	0.02	IL-6 non-inducer
157	PGPGMALKRDYPKYY	0.02	IL-6 non-inducer
158	GPGMALKRDYPKYYP	0.02	IL-6 non-inducer
159	PGMALKRDYPKYYPL	0.12	IL-6 inducer
160	GMALKRDYPKYYPLF	0.2	IL-6 inducer
161	MALKRDYPKYYPLFS	0.16	IL-6 inducer

162	ALKRDYPKYYPLFSG	0.09	IL-6 non-inducer
163	LKRDYPKYYPLFSGP	0.11	IL-6 inducer
164	KRDYPKYYPLFSGPG	0.03	IL-6 non-inducer
165	RDYPKYYPLFSGPGP	0.04	IL-6 non-inducer
166	DYPKYYPLFSGPGPG	0.01	IL-6 non-inducer
167	YPKYYPLFSGPGPGN	0.02	IL-6 non-inducer
168	PKYYPLFSGPGPGNS	0.0	IL-6 non-inducer
169	KYYPLFSGPGPGNSD	0.01	IL-6 non-inducer
170	YYPLFSGPGPGNSDT	0.01	IL-6 non-inducer
171	YPLFSGPGPGNSDTS	0.01	IL-6 non-inducer
172	PLFSGPGPGNSDTSI	0.01	IL-6 non-inducer
173	LFSGPGPGNSDTSIK	0.0	IL-6 non-inducer
174	FSGPGPGNSDTSIKL	0.0	IL-6 non-inducer
175	SGPGPGNSDTSIKLE	0.0	IL-6 non-inducer
176	GPGPGNSDTSIKLEG	0.03	IL-6 non-inducer
177	PGPGNSDTSIKLEGF	0.01	IL-6 non-inducer
178	GPGNSDTSIKLEGFY	0.0	IL-6 non-inducer
179	PGNSDTSIKLEGFYL	0.03	IL-6 non-inducer
180	GNSDTSIKLEGFYLL	0.01	IL-6 non-inducer
181	NSDTSIKLEGFYLLE	0.01	IL-6 non-inducer
182	SDTSIKLEGFYLLEG	0.04	IL-6 non-inducer
183	DTSIKLEGFYLLEGP	0.03	IL-6 non-inducer
184	TSIKLEGFYLLEGPG	0.02	IL-6 non-inducer
185	SIKLEGFYLLEGPGP	0.01	IL-6 non-inducer
186	IKLEGFYLLEGPGPG	0.04	IL-6 non-inducer
187	KLEGFYLLEGPGPGR	0.02	IL-6 non-inducer
188	LEGFYLLEGPGPGRT	0.04	IL-6 non-inducer
189	EGFYLLEGPGPGRTN	0.01	IL-6 non-inducer
190	GFYLLEGPGPGRTNI	0.01	IL-6 non-inducer
191	FYLLEGPGPGRTNIS	0.02	IL-6 non-inducer
192	YLLEGPGPGRTNISY	0.01	IL-6 non-inducer
193	LLEGPGPGRTNISYK	0.02	IL-6 non-inducer
194	LEGPGPGRTNISYKL	0.02	IL-6 non-inducer
195	EGPGPGRTNISYKLT	0.01	IL-6 non-inducer
196	GPGPGRTNISYKLTL	0.02	IL-6 non-inducer
197	PGPGRTNISYKLTLG	0.02	IL-6 non-inducer
198	GPGRTNISYKLTLGT	0.03	IL-6 non-inducer
199	PGRTNISYKLTLGTS	0.02	IL-6 non-inducer
200	GRTNISYKLTLGTSA	0.06	IL-6 non-inducer
201	RTNISYKLTLGTSAQ	0.03	IL-6 non-inducer
202	TNISYKLTLGTSAQG	0.18	IL-6 inducer

203	NISYKLTLGTSAQGP	0.08	IL-6 non-inducer
204	ISYKLTLGTSAQGPG	0.01	IL-6 non-inducer
205	SYKLTLGTSAQGPGP	0.01	IL-6 non-inducer
206	YKLTLGTSAQGPGPG	0.02	IL-6 non-inducer
207	KLTLGTSAQGPGPGI	0.01	IL-6 non-inducer
208	LTLGTSAQGPGPGIH	0.02	IL-6 non-inducer
209	TLGTSAQGPGPGIHT	0.02	IL-6 non-inducer
210	LGTSAQGPGPGIHTK	0.0	IL-6 non-inducer
211	GTSAQGPGPGIHTKM	0.01	IL-6 non-inducer
212	TSAQGPGPGIHTKMD	0.01	IL-6 non-inducer
213	SAQGPGPGIHTKMDR	0.02	IL-6 non-inducer
214	AQGPGPGIHTKMDRT	0.01	IL-6 non-inducer
215	QGPGPGIHTKMDRTK	0.01	IL-6 non-inducer
216	GPGPGIHTKMDRTKG	0.01	IL-6 non-inducer
217	PGPGIHTKMDRTKGV	0.01	IL-6 non-inducer
218	GPGIHTKMDRTKGVE	0.01	IL-6 non-inducer
219	PGIHTKMDRTKGVEV	0.01	IL-6 non-inducer
220	GIHTKMDRTKGVEVL	0.01	IL-6 non-inducer
221	IHTKMDRTKGVEVLN	0.01	IL-6 non-inducer
222	HTKMDRTKGVEVLNG	0.03	IL-6 non-inducer
223	TKMDRTKGVEVLNGP	0.01	IL-6 non-inducer
224	KMDRTKGVEVLNGPG	0.0	IL-6 non-inducer
225	MDRTKGVEVLNGPGP	0.01	IL-6 non-inducer
226	DRTKGVEVLNGPGPG	0.01	IL-6 non-inducer
227	RTKGVEVLNGPGPGG	0.02	IL-6 non-inducer
228	TKGVEVLNGPGPGGM	0.01	IL-6 non-inducer
229	KGVEVLNGPGPGGMH	0.01	IL-6 non-inducer
230	GVEVLNGPGPGGMHI	0.0	IL-6 non-inducer
231	VEVLNGPGPGGMHIM	0.02	IL-6 non-inducer
232	EVLNGPGPGGMHIML	0.03	IL-6 non-inducer
233	VLNGPGPGGMHIMLY	0.05	IL-6 non-inducer
234	LNGPGPGGMHIMLYD	0.04	IL-6 non-inducer
235	NGPGPGGMHIMLYDL	0.03	IL-6 non-inducer
236	GPGPGGMHIMLYDLK	0.03	IL-6 non-inducer
237	PGPGGMHIMLYDLKG	0.04	IL-6 non-inducer
238	GPGGMHIMLYDLKGP	0.03	IL-6 non-inducer
239	PGGMHIMLYDLKGPL	0.05	IL-6 non-inducer
240	GGMHIMLYDLKGPLN	0.06	IL-6 non-inducer
241	GMHIMLYDLKGPLNV	0.15	IL-6 inducer
242	MHIMLYDLKGPLNVA	0.1	IL-6 non-inducer
243	HIMLYDLKGPLNVAA	0.07	IL-6 non-inducer

244	IMLYDLKGPLNVAAY	0.06	IL-6 non-inducer
245	MLYDLKGPLNVAAYR	0.04	IL-6 non-inducer
246	LYDLKGPLNVAAYRV	0.05	IL-6 non-inducer
247	YDLKGPLNVAAYRVN	0.01	IL-6 non-inducer
248	DLKGPLNVAAYRVNF	0.01	IL-6 non-inducer
249	LKGPLNVAAYRVNFF	0.05	IL-6 non-inducer
250	KGPLNVAAYRVNFFT	0.02	IL-6 non-inducer
251	GPLNVAAYRVNFFTP	0.01	IL-6 non-inducer
252	PLNVAAYRVNFFTPK	0.03	IL-6 non-inducer
253	LNVAAYRVNFFTPKM	0.05	IL-6 non-inducer
254	NVAAYRVNFFTPKMA	0.02	IL-6 non-inducer
255	VAAYRVNFFTPKMAA	0.02	IL-6 non-inducer
256	AAYRVNFFTPKMAAY	0.06	IL-6 non-inducer
257	AYRVNFFTPKMAAYS	0.04	IL-6 non-inducer
258	YRVNFFTPKMAAYSS	0.13	IL-6 inducer
259	RVNFFTPKMAAYSSM	0.06	IL-6 non-inducer
260	VNFFTPKMAAYSSME	0.02	IL-6 non-inducer
261	NFFTPKMAAYSSMEN	0.02	IL-6 non-inducer
262	FFTPKMAAYSSMENQ	0.01	IL-6 non-inducer
263	FTPKMAAYSSMENQW	0.02	IL-6 non-inducer
264	TPKMAAYSSMENQWY	0.01	IL-6 non-inducer
265	PKMAAYSSMENQWYL	0.0	IL-6 non-inducer
266	KMAAYSSMENQWYLA	0.06	IL-6 non-inducer
267	MAAYSSMENQWYLAA	0.05	IL-6 non-inducer
268	AAYSSMENQWYLAAY	0.07	IL-6 non-inducer
269	AYSSMENQWYLAAYQ	0.05	IL-6 non-inducer
270	YSSMENQWYLAAYQV	0.02	IL-6 non-inducer
271	SSMENQWYLAAYQVT	0.02	IL-6 non-inducer
272	SMENQWYLAAYQVTA	0.11	IL-6 inducer
273	MENQWYLAAYQVTAG	0.09	IL-6 non-inducer
274	ENQWYLAAYQVTAGT	0.1	IL-6 non-inducer
275	NQWYLAAYQVTAGTG	0.02	IL-6 non-inducer
276	QWYLAAYQVTAGTGG	0.02	IL-6 non-inducer
277	WYLAAYQVTAGTGGW	0.02	IL-6 non-inducer
278	YLAAYQVTAGTGGWA	0.02	IL-6 non-inducer
279	LAAYQVTAGTGGWAA	0.01	IL-6 non-inducer
280	AAYQVTAGTGGWAAY	0.02	IL-6 non-inducer
281	AYQVTAGTGGWAAYT	0.02	IL-6 non-inducer
282	YQVTAGTGGWAAYTA	0.02	IL-6 non-inducer
283	QVTAGTGGWAAYTAN	0.01	IL-6 non-inducer
284	VTAGTGGWAAYTANT	0.01	IL-6 non-inducer

285	TAGTGGWAAYTANTG	0.03	IL-6 non-inducer
286	AGTGGWAAYTANTGG	0.15	IL-6 inducer
287	GTGGWAAYTANTGGN	0.1	IL-6 non-inducer
288	TGGWAAYTANTGGNR	0.05	IL-6 non-inducer
289	GGWAAYTANTGGNRY	0.07	IL-6 non-inducer
290	GWAAYTANTGGNRYA	0.05	IL-6 non-inducer
291	WAAYTANTGGNRYAA	0.04	IL-6 non-inducer
292	AAYTANTGGNRYAAY	0.04	IL-6 non-inducer
293	AYTANTGGNRYAAYK	0.03	IL-6 non-inducer
294	YTANTGGNRYAAYKS	0.09	IL-6 non-inducer
295	TANTGGNRYAAYKST	0.04	IL-6 non-inducer
296	ANTGGNRYAAYKSTR	0.06	IL-6 non-inducer
297	NTGGNRYAAYKSTRG	0.09	IL-6 non-inducer
298	TGGNRYAAYKSTRGN	0.09	IL-6 non-inducer
299	GGNRYAAYKSTRGNI	0.04	IL-6 non-inducer
300	GNRYAAYKSTRGNIV	0.02	IL-6 non-inducer
301	NRYAAYKSTRGNIVK	0.01	IL-6 non-inducer
302	RYAAYKSTRGNIVKA	0.02	IL-6 non-inducer
303	YAAYKSTRGNIVKAA	0.0	IL-6 non-inducer
304	AAYKSTRGNIVKAAY	0.01	IL-6 non-inducer
305	AYKSTRGNIVKAAYK	0.02	IL-6 non-inducer
306	YKSTRGNIVKAAYKT	0.01	IL-6 non-inducer
307	KSTRGNIVKAAYKTT	0.02	IL-6 non-inducer
308	STRGNIVKAAYKTTR	0.01	IL-6 non-inducer
309	TRGNIVKAAYKTTRG	0.02	IL-6 non-inducer
310	RGNIVKAAYKTTRGT	0.02	IL-6 non-inducer
311	GNIVKAAYKTTRGTV	0.01	IL-6 non-inducer
312	NIVKAAYKTTRGTVG	0.01	IL-6 non-inducer
313	IVKAAYKTTRGTVGL	0.07	IL-6 non-inducer
314	VKAAYKTTRGTVGLA	0.02	IL-6 non-inducer
315	KAAYKTTRGTVGLAA	0.04	IL-6 non-inducer
316	AAYKTTRGTVGLAAY	0.02	IL-6 non-inducer
317	AYKTTRGTVGLAAYS	0.02	IL-6 non-inducer
318	YKTTRGTVGLAAYSS	0.02	IL-6 non-inducer
319	KTTRGTVGLAAYSSF	0.01	IL-6 non-inducer
320	TTRGTVGLAAYSSFK	0.01	IL-6 non-inducer
321	TRGTVGLAAYSSFKI	0.01	IL-6 non-inducer
322	RGTVGLAAYSSFKIT	0.0	IL-6 non-inducer
323	GTVGLAAYSSFKITF	0.0	IL-6 non-inducer
324	TVGLAAYSSFKITFF	0.01	IL-6 non-inducer
325	VGLAAYSSFKITFFF	0.01	IL-6 non-inducer

326	GLAAYSSFKITFFFA	0.01	IL-6 non-inducer
327	LAAYSSFKITFFFAA	0.04	IL-6 non-inducer
328	AAYSSFKITFFFAAY	0.09	IL-6 non-inducer
329	AYSSFKITFFFAAYP	0.06	IL-6 non-inducer
330	YSSFKITFFFAAYPY	0.1	IL-6 non-inducer
331	SSFKITFFFAAYPYI	0.11	IL-6 inducer
332	SFKITFFFAAYPYIF	0.16	IL-6 inducer
333	FKITFFFAAYPYIFI	0.26	IL-6 inducer
334	KITFFFAAYPYIFIA	0.16	IL-6 inducer
335	ITFFFAAYPYIFIAT	0.07	IL-6 non-inducer
336	TFFFAAYPYIFIATF	0.08	IL-6 non-inducer
337	FFFAAYPYIFIATFF	0.11	IL-6 inducer
338	FFAAYPYIFIATFFA	0.09	IL-6 non-inducer
339	FAAYPYIFIATFFAA	0.06	IL-6 non-inducer
340	AAYPYIFIATFFAAY	0.06	IL-6 non-inducer
341	AYPYIFIATFFAAYF	0.11	IL-6 inducer
342	YPYIFIATFFAAYFL	0.14	IL-6 inducer
343	PYIFIATFFAAYFLL	0.15	IL-6 inducer
344	YIFIATFFAAYFLLP	0.15	IL-6 inducer
345	IFIATFFAAYFLLPY	0.15	IL-6 inducer
346	FIATFFAAYFLLPYI	0.14	IL-6 inducer
347	IATFFAAYFLLPYIF	0.14	IL-6 inducer
348	ATFFAAYFLLPYIFI	0.14	IL-6 inducer
349	TFFAAYFLLPYIFIA	0.14	IL-6 inducer
350	FFAAYFLLPYIFIAA	0.12	IL-6 inducer
351	FAAYFLLPYIFIAAA	0.08	IL-6 non-inducer
352	AAYFLLPYIFIAAAY	0.08	IL-6 non-inducer
353	AYFLLPYIFIAAAYG	0.08	IL-6 non-inducer
354	YFLLPYIFIAAAYGQ	0.06	IL-6 non-inducer
355	FLLPYIFIAAAYGQI	0.03	IL-6 non-inducer
356	LLPYIFIAAAYGQIG	0.01	IL-6 non-inducer
357	LPYIFIAAAYGQIGV	0.0	IL-6 non-inducer
358	PYIFIAAAYGQIGVD	0.0	IL-6 non-inducer
359	YIFIAAAYGQIGVDF	0.06	IL-6 non-inducer
360	IFIAAAYGQIGVDFY	0.06	IL-6 non-inducer
361	FIAAAYGQIGVDFYA	0.02	IL-6 non-inducer
362	IAAAYGQIGVDFYAA	0.01	IL-6 non-inducer
363	AAAYGQIGVDFYAAA	0.02	IL-6 non-inducer
364	AAYGQIGVDFYAAAY	0.01	IL-6 non-inducer
365	AYGQIGVDFYAAAYN	0.1	IL-6 non-inducer
366	YGQIGVDFYAAAYNE	0.02	IL-6 non-inducer

367	GQIGVDFYAAAYNEC	0.05	IL-6 non-inducer
368	QIGVDFYAAAYNECG	0.06	IL-6 non-inducer
369	IGVDFYAAAYNECGE	0.05	IL-6 non-inducer
370	GVDFYAAAYNECGEV	0.06	IL-6 non-inducer
371	VDFYAAAYNECGEVN	0.04	IL-6 non-inducer
372	DFYAAAYNECGEVNP	0.05	IL-6 non-inducer
373	FYAAAYNECGEVNPI	0.04	IL-6 non-inducer
374	YAAAYNECGEVNPIA	0.03	IL-6 non-inducer
375	AAAYNECGEVNPIAA	0.04	IL-6 non-inducer
376	AAYNECGEVNPIAAY	0.02	IL-6 non-inducer
377	AYNECGEVNPIAAYG	0.02	IL-6 non-inducer
378	YNECGEVNPIAAYGT	0.03	IL-6 non-inducer
379	NECGEVNPIAAYGTV	0.03	IL-6 non-inducer
380	ECGEVNPIAAYGTVI	0.02	IL-6 non-inducer
381	CGEVNPIAAYGTVIN	0.02	IL-6 non-inducer
382	GEVNPIAAYGTVINV	0.02	IL-6 non-inducer
383	EVNPIAAYGTVINVH	0.04	IL-6 non-inducer
384	VNPIAAYGTVINVHL	0.01	IL-6 non-inducer
385	NPIAAYGTVINVHLK	0.02	IL-6 non-inducer
386	PIAAYGTVINVHLKA	0.0	IL-6 non-inducer
387	IAAYGTVINVHLKAA	0.01	IL-6 non-inducer
388	AAYGTVINVHLKAAY	0.01	IL-6 non-inducer
389	AYGTVINVHLKAAYT	0.01	IL-6 non-inducer
390	YGTVINVHLKAAYTG	0.02	IL-6 non-inducer
391	GTVINVHLKAAYTGQ	0.02	IL-6 non-inducer
392	TVINVHLKAAYTGQF	0.02	IL-6 non-inducer
393	VINVHLKAAYTGQFH	0.07	IL-6 non-inducer
394	INVHLKAAYTGQFHI	0.1	IL-6 non-inducer
395	NVHLKAAYTGQFHIA	0.1	IL-6 non-inducer
396	VHLKAAYTGQFHIAP	0.01	IL-6 non-inducer
397	HLKAAYTGQFHIAPY	0.04	IL-6 non-inducer

TABLE VII

Prediction of stimulation of TNFepitope in each epitope of our construct

Seq ID	Pattern ID	Start	End	Sequence	ML_Score	Hybrid_score	Prediction
heat	Pattern_1	1	15	KNKINFAYQLSLGTS	0.44	0.44	TNF non-inducer
heat	Pattern_2	2	16	NKINFAYQLSLGTSG	0.43	0.43	TNF non-inducer
heat	Pattern_3	3	17	KINFAYQLSLGTSGP	0.4	0.4	TNF non-inducer
heat	Pattern_4	4	18	INFAYQLSLGTSGPG	0.37	0.37	TNF non-inducer
heat	Pattern_5	5	19	NFAYQLSLGTSGPGP	0.33	0.33	TNF non-inducer
heat	Pattern_6	6	20	FAYQLSLGTSGPGPG	0.36	0.36	TNF non-inducer
heat	Pattern_7	7	21	AYQLSLGTSGPGPGA	0.33	0.33	TNF non-inducer
heat	Pattern_8	8	22	YQLSLGTSGPGPGAT	0.36	0.36	TNF non-inducer
heat	Pattern_9	9	23	QLSLGTSGPGPGATS	0.36	0.36	TNF non-inducer
heat	Pattern_10	10	24	LSLGTSGPGPGATSV	0.37	0.37	TNF non-inducer
heat	Pattern_11	11	25	SLGTSGPGPGATSVK	0.35	0.35	TNF non-inducer
heat	Pattern_12	12	26	LGTSGPGPGATSVKL	0.35	0.35	TNF non-inducer
heat	Pattern_13	13	27	GTSGPGPGATSVKLK	0.41	0.41	TNF non-inducer
heat	Pattern_14	14	28	TSGPGPGATSVKLKS	0.41	0.41	TNF non-inducer
heat	Pattern_15	15	29	SGPGPGATSVKLKSN	0.42	0.42	TNF non-inducer
heat	Pattern_16	16	30	GPGPGATSVKLKSNM	0.46	0.46	TNF-inducer
heat	Pattern_17	17	31	PGPGATSVKLKSNMT	0.45	0.45	TNF non-inducer
heat	Pattern_18	18	32	GPGATSVKLKSNMTV	0.42	0.42	TNF non-inducer
heat	Pattern_19	19	33	PGATSVKLKSNMTVS	0.44	0.44	TNF non-inducer
heat	Pattern_20	20	34	GATSVKLKSNMTVSV	0.49	0.49	TNF-inducer
heat	Pattern_21	21	35	ATSVKLKSNMTVSVD	0.54	0.54	TNF-inducer
heat	Pattern_22	22	36	TSVKLKSNMTVSVDG	0.48	0.48	TNF-inducer
heat	Pattern_23	23	37	SVKLKSNMTVSVDGP	0.48	0.48	TNF-inducer
heat	Pattern_24	24	38	VKLKSNMTVSVDGPG	0.46	0.46	TNF-inducer
heat	Pattern_25	25	39	KLKSNMTVSVDGPGP	0.48	0.48	TNF-inducer
heat	Pattern_26	26	40	LKSNMTVSVDGPGPG	0.47	0.47	TNF-inducer
heat	Pattern_27	27	41	KSNMTVSVDGPGPGP	0.43	0.43	TNF non-inducer
heat	Pattern_28	28	42	SNMTVSVDGPGPGPK	0.38	0.38	TNF non-inducer
heat	Pattern_29	29	43	NMTVSVDGPGPGPKL	0.42	0.42	TNF non-inducer
heat	Pattern_30	30	44	MTVSVDGPGPGPKLE	0.4	0.4	TNF non-inducer
heat	Pattern_31	31	45	TVSVDGPGPGPKLEY	0.37	0.37	TNF non-inducer
heat	Pattern_32	32	46	VSVDGPGPGPKLEYK	0.41	0.41	TNF non-inducer
heat	Pattern_33	33	47	SVDGPGPGPKLEYKP	0.34	0.34	TNF non-inducer
heat	Pattern_34	34	48	VDGPGPGPKLEYKPN	0.35	0.35	TNF non-inducer
heat	Pattern_35	35	49	DGPGPGPKLEYKPNL	0.37	0.37	TNF non-inducer
heat	Pattern_36	36	50	GPGPGPKLEYKPNLV	0.4	0.4	TNF non-inducer
heat	Pattern_37	37	51	PGPGPKLEYKPNLVG	0.38	0.38	TNF non-inducer
heat	Pattern_38	38	52	GPGPKLEYKPNLVGN	0.32	0.32	TNF non-inducer

heat	Pattern_39	39	53	PGPKLEYKPNLVGNK	0.3	0.3	TNF non-inducer
heat	Pattern_40	40	54	GPKLEYKPNLVGNKT	0.31	0.31	TNF non-inducer
heat	Pattern_41	41	55	PKLEYKPNLVGNKTV	0.28	0.28	TNF non-inducer
heat	Pattern_42	42	56	KLEYKPNLVGNKTVG	0.36	0.36	TNF non-inducer
heat	Pattern_43	43	57	LEYKPNLVGNKTVGP	0.3	0.3	TNF non-inducer
heat	Pattern_44	44	58	EYKPNLVGNKTVGPG	0.32	0.32	TNF non-inducer
heat	Pattern_45	45	59	YKPNLVGNKTVGPGP	0.35	0.35	TNF non-inducer
heat	Pattern_46	46	60	KPNLVGNKTVGPGPG	0.37	0.37	TNF non-inducer
heat	Pattern_47	47	61	PNLVGNKTVGPGPGN	0.45	0.45	TNF non-inducer
heat	Pattern_48	48	62	NLVGNKTVGPGPGNA	0.44	0.44	TNF non-inducer
heat	Pattern_49	49	63	LVGNKTVGPGPGNAN	0.39	0.39	TNF non-inducer
heat	Pattern_50	50	64	VGNKTVGPGPGNANG	0.38	0.38	TNF non-inducer
heat	Pattern_51	51	65	GNKTVGPGPGNANGI	0.34	0.34	TNF non-inducer
heat	Pattern_52	52	66	NKTVGPGPGNANGIT	0.33	0.33	TNF non-inducer
heat	Pattern_53	53	67	KTVGPGPGNANGITL	0.36	0.36	TNF non-inducer
heat	Pattern_54	54	68	TVGPGPGNANGITLT	0.34	0.34	TNF non-inducer
heat	Pattern_55	55	69	VGPGPGNANGITLTV	0.34	0.34	TNF non-inducer
heat	Pattern_56	56	70	GPGPGNANGITLTVN	0.36	0.36	TNF non-inducer
heat	Pattern_57	57	71	PGPGNANGITLTVNN	0.33	0.33	TNF non-inducer
heat	Pattern_58	58	72	GPGNANGITLTVNNP	0.31	0.31	TNF non-inducer
heat	Pattern_59	59	73	PGNANGITLTVNNPS	0.32	0.32	TNF non-inducer
heat	Pattern_60	60	74	GNANGITLTVNNPSL	0.37	0.37	TNF non-inducer
heat	Pattern_61	61	75	NANGITLTVNNPSLT	0.48	0.48	TNF-inducer
heat	Pattern_62	62	76	ANGITLTVNNPSLTG	0.43	0.43	TNF non-inducer
heat	Pattern_63	63	77	NGITLTVNNPSLTGP	0.43	0.43	TNF non-inducer
heat	Pattern_64	64	78	GITLTVNNPSLTGPG	0.46	0.46	TNF-inducer
heat	Pattern_65	65	79	ITLTVNNPSLTGPGP	0.45	0.45	TNF non-inducer
heat	Pattern_66	66	80	TLTVNNPSLTGPGPG	0.44	0.44	TNF non-inducer
heat	Pattern_67	67	81	LTVNNPSLTGPGPGM	0.41	0.41	TNF non-inducer
heat	Pattern_68	68	82	TVNNPSLTGPGPGME	0.38	0.38	TNF non-inducer
heat	Pattern_69	69	83	VNNPSLTGPGPGMEN	0.38	0.38	TNF non-inducer
heat	Pattern_70	70	84	NNPSLTGPGPGMENQ	0.4	0.4	TNF non-inducer
heat	Pattern_71	71	85	NPSLTGPGPGMENQW	0.39	0.39	TNF non-inducer
heat	Pattern_72	72	86	PSLTGPGPGMENQWY	0.48	0.48	TNF-inducer
heat	Pattern_73	73	87	SLTGPGPGMENQWYL	0.48	0.48	TNF-inducer
heat	Pattern_74	74	88	LTGPGPGMENQWYLK	0.47	0.47	TNF-inducer
heat	Pattern_75	75	89	TGPGPGMENQWYLKL	0.5	0.5	TNF-inducer
heat	Pattern_76	76	90	GPGPGMENQWYLKLN	0.56	0.56	TNF-inducer
heat	Pattern_77	77	91	PGPGMENQWYLKLNA	0.57	0.57	TNF-inducer
heat	Pattern_78	78	92	GPGMENQWYLKLNAG	0.56	0.56	TNF-inducer
heat	Pattern_79	79	93	PGMENQWYLKLNAGT	0.6	0.6	TNF-inducer

heat	Pattern_80	80	94	GMENQWYLKLNAGTM	0.61	0.61	TNF-inducer
heat	Pattern_81	81	95	MENQWYLKLNAGTMI	0.59	0.59	TNF-inducer
heat	Pattern_82	82	96	ENQWYLKLNAGTMIG	0.58	0.58	TNF-inducer
heat	Pattern_83	83	97	NQWYLKLNAGTMIGP	0.58	0.58	TNF-inducer
heat	Pattern_84	84	98	QWYLKLNAGTMIGPG	0.57	0.57	TNF-inducer
heat	Pattern_85	85	99	WYLKLNAGTMIGPGP	0.58	0.58	TNF-inducer
heat	Pattern_86	86	100	YLKLNAGTMIGPGPG	0.53	0.53	TNF-inducer
heat	Pattern_87	87	101	LKLNAGTMIGPGPGE	0.5	0.5	TNF-inducer
heat	Pattern_88	88	102	KLNAGTMIGPGPGEY	0.47	0.47	TNF-inducer
heat	Pattern_89	89	103	LNAGTMIGPGPGEYK	0.43	0.43	TNF non-inducer
heat	Pattern_90	90	104	NAGTMIGPGPGEYKI	0.39	0.39	TNF non-inducer
heat	Pattern_91	91	105	AGTMIGPGPGEYKIM	0.4	0.4	TNF non-inducer
heat	Pattern_92	92	106	GTMIGPGPGEYKIMP	0.41	0.41	TNF non-inducer
heat	Pattern_93	93	107	TMIGPGPGEYKIMPG	0.42	0.42	TNF non-inducer
heat	Pattern_94	94	108	MIGPGPGEYKIMPGL	0.42	0.42	TNF non-inducer
heat	Pattern_95	95	109	IGPGPGEYKIMPGLL	0.44	0.44	TNF non-inducer
heat	Pattern_96	96	110	GPGPGEYKIMPGLLP	0.43	0.43	TNF non-inducer
heat	Pattern_97	97	111	PGPGEYKIMPGLLPY	0.41	0.41	TNF non-inducer
heat	Pattern_98	98	112	GPGEYKIMPGLLPYA	0.37	0.37	TNF non-inducer
heat	Pattern_99	99	113	PGEYKIMPGLLPYAE	0.39	0.39	TNF non-inducer
heat	Pattern_100	100	114	GEYKIMPGLLPYAEI	0.39	0.39	TNF non-inducer
heat	Pattern_101	101	115	EYKIMPGLLPYAEIS	0.4	0.4	TNF non-inducer
heat	Pattern_102	102	116	YKIMPGLLPYAEISG	0.34	0.34	TNF non-inducer
heat	Pattern_103	103	117	KIMPGLLPYAEISGP	0.33	0.33	TNF non-inducer
heat	Pattern_104	104	118	IMPGLLPYAEISGPG	0.32	0.32	TNF non-inducer
heat	Pattern_105	105	119	MPGLLPYAEISGPGP	0.33	0.33	TNF non-inducer
heat	Pattern_106	106	120	PGLLPYAEISGPGPG	0.33	0.33	TNF non-inducer
heat	Pattern_107	107	121	GLLPYAEISGPGPGS	0.24	0.24	TNF non-inducer
heat	Pattern_108	108	122	LLPYAEISGPGPGSK	0.27	0.27	TNF non-inducer
heat	Pattern_109	109	123	LPYAEISGPGPGSKM	0.26	0.26	TNF non-inducer
heat	Pattern_110	110	124	PYAEISGPGPGSKMV	0.26	0.26	TNF non-inducer
heat	Pattern_111	111	125	YAEISGPGPGSKMVK	0.26	0.26	TNF non-inducer
heat	Pattern_112	112	126	AEISGPGPGSKMVKV	0.27	0.27	TNF non-inducer
heat	Pattern_113	113	127	EISGPGPGSKMVKVD	0.28	0.28	TNF non-inducer
heat	Pattern_114	114	128	ISGPGPGSKMVKVDY	0.29	0.29	TNF non-inducer
heat	Pattern_115	115	129	SGPGPGSKMVKVDYP	0.28	0.28	TNF non-inducer
heat	Pattern_116	116	130	GPGPGSKMVKVDYPF	0.35	0.35	TNF non-inducer
heat	Pattern_117	117	131	PGPGSKMVKVDYPFL	0.33	0.33	TNF non-inducer
heat	Pattern_118	118	132	GPGSKMVKVDYPFLI	0.33	0.33	TNF non-inducer
heat	Pattern_119	119	133	PGSKMVKVDYPFLIA	0.36	0.36	TNF non-inducer
heat	Pattern_120	120	134	GSKMVKVDYPFLIAD	0.37	0.37	TNF non-inducer

heat	Pattern_121	121	135	SKMVKVDYPFLIADN	0.49	0.49	TNF-inducer
heat	Pattern_122	122	136	KMVKVDYPFLIADNG	0.46	0.46	TNF-inducer
heat	Pattern_123	123	137	MVKVDYPFLIADNGP	0.45	0.45	TNF non-inducer
heat	Pattern_124	124	138	VKVDYPFLIADNGPG	0.4	0.4	TNF non-inducer
heat	Pattern_125	125	139	KVDYPFLIADNGPGP	0.46	0.46	TNF-inducer
heat	Pattern_126	126	140	VDYPFLIADNGPGPG	0.48	0.48	TNF-inducer
heat	Pattern_127	127	141	DYPFLIADNGPGPGY	0.46	0.46	TNF-inducer
heat	Pattern_128	128	142	YPFLIADNGPGPGYQ	0.43	0.43	TNF non-inducer
heat	Pattern_129	129	143	PFLIADNGPGPGYQL	0.39	0.39	TNF non-inducer
heat	Pattern_130	130	144	FLIADNGPGPGYQLS	0.33	0.33	TNF non-inducer
heat	Pattern_131	131	145	LIADNGPGPGYQLSL	0.39	0.39	TNF non-inducer
heat	Pattern_132	132	146	IADNGPGPGYQLSLG	0.38	0.38	TNF non-inducer
heat	Pattern_133	133	147	ADNGPGPGYQLSLGT	0.36	0.36	TNF non-inducer
heat	Pattern_134	134	148	DNGPGPGYQLSLGTS	0.34	0.34	TNF non-inducer
heat	Pattern_135	135	149	NGPGPGYQLSLGTSF	0.35	0.35	TNF non-inducer
heat	Pattern_136	136	150	GPGPGYQLSLGTSFE	0.35	0.35	TNF non-inducer
heat	Pattern_137	137	151	PGPGYQLSLGTSFEV	0.35	0.35	TNF non-inducer
heat	Pattern_138	138	152	GPGYQLSLGTSFEVA	0.33	0.33	TNF non-inducer
heat	Pattern_139	139	153	PGYQLSLGTSFEVAQ	0.33	0.33	TNF non-inducer
heat	Pattern_140	140	154	GYQLSLGTSFEVAQG	0.35	0.35	TNF non-inducer
heat	Pattern_141	141	155	YQLSLGTSFEVAQGV	0.38	0.38	TNF non-inducer
heat	Pattern_142	142	156	QLSLGTSFEVAQGVG	0.36	0.36	TNF non-inducer
heat	Pattern_143	143	157	LSLGTSFEVAQGVGP	0.36	0.36	TNF non-inducer
heat	Pattern_144	144	158	SLGTSFEVAQGVGPG	0.39	0.39	TNF non-inducer
heat	Pattern_145	145	159	LGTSFEVAQGVGPGP	0.37	0.37	TNF non-inducer
heat	Pattern_146	146	160	GTSFEVAQGVGPGPG	0.38	0.38	TNF non-inducer
heat	Pattern_147	147	161	TSFEVAQGVGPGPGM	0.38	0.38	TNF non-inducer
heat	Pattern_148	148	162	SFEVAQGVGPGPGMA	0.43	0.43	TNF non-inducer
heat	Pattern_149	149	163	FEVAQGVGPGPGMAL	0.44	0.44	TNF non-inducer
heat	Pattern_150	150	164	EVAQGVGPGPGMALK	0.52	0.52	TNF-inducer
heat	Pattern_151	151	165	VAQGVGPGPGMALKR	0.53	0.53	TNF-inducer
heat	Pattern_152	152	166	AQGVGPGPGMALKRD	0.51	0.51	TNF-inducer
heat	Pattern_153	153	167	QGVGPGPGMALKRDY	0.52	0.52	TNF-inducer
heat	Pattern_154	154	168	GVGPGPGMALKRDYP	0.53	0.53	TNF-inducer
heat	Pattern_155	155	169	VGPGPGMALKRDYPK	0.48	0.48	TNF-inducer
heat	Pattern_156	156	170	GPGPGMALKRDYPKY	0.5	0.5	TNF-inducer
heat	Pattern_157	157	171	PGPGMALKRDYPKYY	0.51	0.51	TNF-inducer
heat	Pattern_158	158	172	GPGMALKRDYPKYYP	0.53	0.53	TNF-inducer
heat	Pattern_159	159	173	PGMALKRDYPKYYPL	0.49	0.49	TNF-inducer
heat	Pattern_160	160	174	GMALKRDYPKYYPLF	0.56	0.56	TNF-inducer
heat	Pattern_161	161	175	MALKRDYPKYYPLFS	0.54	0.54	TNF-inducer

heat	Pattern_162	162	176	ALKRDYPKYYPLFSG	0.47	0.47	TNF-inducer
heat	Pattern_163	163	177	LKRDYPKYYPLFSGP	0.41	0.41	TNF non-inducer
heat	Pattern_164	164	178	KRDYPKYYPLFSGPG	0.32	0.32	TNF non-inducer
heat	Pattern_165	165	179	RDYPKYYPLFSGPGP	0.34	0.34	TNF non-inducer
heat	Pattern_166	166	180	DYPKYYPLFSGPGPG	0.37	0.37	TNF non-inducer
heat	Pattern_167	167	181	YPKYYPLFSGPGPGN	0.31	0.31	TNF non-inducer
heat	Pattern_168	168	182	PKYYPLFSGPGPGNS	0.3	0.3	TNF non-inducer
heat	Pattern_169	169	183	KYYPLFSGPGPGNSD	0.27	0.27	TNF non-inducer
heat	Pattern_170	170	184	YYPLFSGPGPGNSDT	0.29	0.29	TNF non-inducer
heat	Pattern_171	171	185	YPLFSGPGPGNSDTS	0.25	0.25	TNF non-inducer
heat	Pattern_172	172	186	PLFSGPGPGNSDTSI	0.21	0.21	TNF non-inducer
heat	Pattern_173	173	187	LFSGPGPGNSDTSIK	0.19	0.19	TNF non-inducer
heat	Pattern_174	174	188	FSGPGPGNSDTSIKL	0.22	0.22	TNF non-inducer
heat	Pattern_175	175	189	SGPGPGNSDTSIKLE	0.21	0.21	TNF non-inducer
heat	Pattern_176	176	190	GPGPGNSDTSIKLEG	0.25	0.25	TNF non-inducer
heat	Pattern_177	177	191	PGPGNSDTSIKLEGF	0.24	0.24	TNF non-inducer
heat	Pattern_178	178	192	GPGNSDTSIKLEGFY	0.22	0.22	TNF non-inducer
heat	Pattern_179	179	193	PGNSDTSIKLEGFYL	0.26	0.26	TNF non-inducer
heat	Pattern_180	180	194	GNSDTSIKLEGFYLL	0.27	0.27	TNF non-inducer
heat	Pattern_181	181	195	NSDTSIKLEGFYLLE	0.32	0.32	TNF non-inducer
heat	Pattern_182	182	196	SDTSIKLEGFYLLEG	0.31	0.31	TNF non-inducer
heat	Pattern_183	183	197	DTSIKLEGFYLLEGP	0.33	0.33	TNF non-inducer
heat	Pattern_184	184	198	TSIKLEGFYLLEGPG	0.31	0.31	TNF non-inducer
heat	Pattern_185	185	199	SIKLEGFYLLEGPGP	0.36	0.36	TNF non-inducer
heat	Pattern_186	186	200	IKLEGFYLLEGPGPG	0.43	0.43	TNF non-inducer
heat	Pattern_187	187	201	KLEGFYLLEGPGPGR	0.48	0.48	TNF-inducer
heat	Pattern_188	188	202	LEGFYLLEGPGPGRT	0.42	0.42	TNF non-inducer
heat	Pattern_189	189	203	EGFYLLEGPGPGRTN	0.39	0.39	TNF non-inducer
heat	Pattern_190	190	204	GFYLLEGPGPGRTNI	0.41	0.41	TNF non-inducer
heat	Pattern_191	191	205	FYLLEGPGPGRTNIS	0.39	0.39	TNF non-inducer
heat	Pattern_192	192	206	YLLEGPGPGRTNISY	0.44	0.44	TNF non-inducer
heat	Pattern_193	193	207	LLEGPGPGRTNISYK	0.42	0.42	TNF non-inducer
heat	Pattern_194	194	208	LEGPGPGRTNISYKL	0.46	0.46	TNF-inducer
heat	Pattern_195	195	209	EGPGPGRTNISYKLT	0.43	0.43	TNF non-inducer
heat	Pattern_196	196	210	GPGPGRTNISYKLTL	0.43	0.43	TNF non-inducer
heat	Pattern_197	197	211	PGPGRTNISYKLTLG	0.42	0.42	TNF non-inducer
heat	Pattern_198	198	212	GPGRTNISYKLTLGT	0.4	0.4	TNF non-inducer
heat	Pattern_199	199	213	PGRTNISYKLTLGTS	0.39	0.39	TNF non-inducer
heat	Pattern_200	200	214	GRTNISYKLTLGTSA	0.44	0.44	TNF non-inducer
heat	Pattern_201	201	215	RTNISYKLTLGTSAQ	0.43	0.43	TNF non-inducer
heat	Pattern_202	202	216	TNISYKLTLGTSAQG	0.45	0.45	TNF non-inducer

heat	Pattern_203	203	217	NISYKLTLGTSAQGP	0.43	0.43	TNF non-inducer
heat	Pattern_204	204	218	ISYKLTLGTSAQGPG	0.39	0.39	TNF non-inducer
heat	Pattern_205	205	219	SYKLTLGTSAQGPGP	0.41	0.41	TNF non-inducer
heat	Pattern_206	206	220	YKLTLGTSAQGPGPG	0.41	0.41	TNF non-inducer
heat	Pattern_207	207	221	KLTLGTSAQGPGPGI	0.42	0.42	TNF non-inducer
heat	Pattern_208	208	222	LTLGTSAQGPGPGIH	0.39	0.39	TNF non-inducer
heat	Pattern_209	209	223	TLGTSAQGPGPGIHT	0.39	0.39	TNF non-inducer
heat	Pattern_210	210	224	LGTSAQGPGPGIHTK	0.36	0.36	TNF non-inducer
heat	Pattern_211	211	225	GTSAQGPGPGIHTKM	0.36	0.36	TNF non-inducer
heat	Pattern_212	212	226	TSAQGPGPGIHTKMD	0.32	0.32	TNF non-inducer
heat	Pattern_213	213	227	SAQGPGPGIHTKMDR	0.34	0.34	TNF non-inducer
heat	Pattern_214	214	228	AQGPGPGIHTKMDRT	0.32	0.32	TNF non-inducer
heat	Pattern_215	215	229	QGPGPGIHTKMDRTK	0.32	0.32	TNF non-inducer
heat	Pattern_216	216	230	GPGPGIHTKMDRTKG	0.33	0.33	TNF non-inducer
heat	Pattern_217	217	231	PGPGIHTKMDRTKGV	0.35	0.35	TNF non-inducer
heat	Pattern_218	218	232	GPGIHTKMDRTKGVE	0.34	0.34	TNF non-inducer
heat	Pattern_219	219	233	PGIHTKMDRTKGVEV	0.33	0.33	TNF non-inducer
heat	Pattern_220	220	234	GIHTKMDRTKGVEVL	0.35	0.35	TNF non-inducer
heat	Pattern_221	221	235	IHTKMDRTKGVEVLN	0.42	0.42	TNF non-inducer
heat	Pattern_222	222	236	HTKMDRTKGVEVLNG	0.37	0.37	TNF non-inducer
heat	Pattern_223	223	237	TKMDRTKGVEVLNGP	0.39	0.39	TNF non-inducer
heat	Pattern_224	224	238	KMDRTKGVEVLNGPG	0.37	0.37	TNF non-inducer
heat	Pattern_225	225	239	MDRTKGVEVLNGPGP	0.41	0.41	TNF non-inducer
heat	Pattern_226	226	240	DRTKGVEVLNGPGPG	0.44	0.44	TNF non-inducer
heat	Pattern_227	227	241	RTKGVEVLNGPGPGG	0.45	0.45	TNF non-inducer
heat	Pattern_228	228	242	TKGVEVLNGPGPGGM	0.44	0.44	TNF non-inducer
heat	Pattern_229	229	243	KGVEVLNGPGPGGMH	0.47	0.47	TNF-inducer
heat	Pattern_230	230	244	GVEVLNGPGPGGMHI	0.49	0.49	TNF-inducer
heat	Pattern_231	231	245	VEVLNGPGPGGMHIM	0.48	0.48	TNF-inducer
heat	Pattern_232	232	246	EVLNGPGPGGMHIML	0.47	0.47	TNF-inducer
heat	Pattern_233	233	247	VLNGPGPGGMHIMLY	0.51	0.51	TNF-inducer
heat	Pattern_234	234	248	LNGPGPGGMHIMLYD	0.5	0.5	TNF-inducer
heat	Pattern_235	235	249	NGPGPGGMHIMLYDL	0.46	0.46	TNF-inducer
heat	Pattern_236	236	250	GPGPGGMHIMLYDLK	0.53	0.53	TNF-inducer
heat	Pattern_237	237	251	PGPGGMHIMLYDLKG	0.51	0.51	TNF-inducer
heat	Pattern_238	238	252	GPGGMHIMLYDLKGP	0.51	0.51	TNF-inducer
heat	Pattern_239	239	253	PGGMHIMLYDLKGPL	0.44	0.44	TNF non-inducer
heat	Pattern_240	240	254	GGMHIMLYDLKGPLN	0.49	0.49	TNF-inducer
heat	Pattern_241	241	255	GMHIMLYDLKGPLNV	0.52	0.52	TNF-inducer
heat	Pattern_242	242	256	MHIMLYDLKGPLNVA	0.55	0.55	TNF-inducer
heat	Pattern_243	243	257	HIMLYDLKGPLNVAA	0.55	0.55	TNF-inducer

heat	Pattern_244	244	258	IMLYDLKGPLNVAAY	0.54	0.54	TNF-inducer
heat	Pattern_245	245	259	MLYDLKGPLNVAAYR	0.52	0.52	TNF-inducer
heat	Pattern_246	246	260	LYDLKGPLNVAAYRV	0.49	0.49	TNF-inducer
heat	Pattern_247	247	261	YDLKGPLNVAAYRVN	0.48	0.48	TNF-inducer
heat	Pattern_248	248	262	DLKGPLNVAAYRVNF	0.48	0.48	TNF-inducer
heat	Pattern_249	249	263	LKGPLNVAAYRVNFF	0.49	0.49	TNF-inducer
heat	Pattern_250	250	264	KGPLNVAAYRVNFFT	0.45	0.45	TNF non-inducer
heat	Pattern_251	251	265	GPLNVAAYRVNFFTP	0.41	0.41	TNF non-inducer
heat	Pattern_252	252	266	PLNVAAYRVNFFTPK	0.42	0.42	TNF non-inducer
heat	Pattern_253	253	267	LNVAAYRVNFFTPKM	0.49	0.49	TNF-inducer
heat	Pattern_254	254	268	NVAAYRVNFFTPKMA	0.45	0.45	TNF non-inducer
heat	Pattern_255	255	269	VAAYRVNFFTPKMAA	0.43	0.43	TNF non-inducer
heat	Pattern_256	256	270	AAYRVNFFTPKMAAY	0.42	0.42	TNF non-inducer
heat	Pattern_257	257	271	AYRVNFFTPKMAAYS	0.35	0.35	TNF non-inducer
heat	Pattern_258	258	272	YRVNFFTPKMAAYSS	0.33	0.33	TNF non-inducer
heat	Pattern_259	259	273	RVNFFTPKMAAYSSM	0.29	0.29	TNF non-inducer
heat	Pattern_260	260	274	VNFFTPKMAAYSSME	0.31	0.31	TNF non-inducer
heat	Pattern_261	261	275	NFFTPKMAAYSSMEN	0.29	0.29	TNF non-inducer
heat	Pattern_262	262	276	FFTPKMAAYSSMENQ	0.3	0.3	TNF non-inducer
heat	Pattern_263	263	277	FTPKMAAYSSMENQW	0.29	0.29	TNF non-inducer
heat	Pattern_264	264	278	TPKMAAYSSMENQWY	0.42	0.42	TNF non-inducer
heat	Pattern_265	265	279	PKMAAYSSMENQWYL	0.42	0.42	TNF non-inducer
heat	Pattern_266	266	280	KMAAYSSMENQWYLA	0.42	0.42	TNF non-inducer
heat	Pattern_267	267	281	MAAYSSMENQWYLAA	0.44	0.44	TNF non-inducer
heat	Pattern_268	268	282	AAYSSMENQWYLAAY	0.41	0.41	TNF non-inducer
heat	Pattern_269	269	283	AYSSMENQWYLAAYQ	0.39	0.39	TNF non-inducer
heat	Pattern_270	270	284	YSSMENQWYLAAYQV	0.37	0.37	TNF non-inducer
heat	Pattern_271	271	285	SSMENQWYLAAYQVT	0.42	0.42	TNF non-inducer
heat	Pattern_272	272	286	SMENQWYLAAYQVTA	0.42	0.42	TNF non-inducer
heat	Pattern_273	273	287	MENQWYLAAYQVTAG	0.46	0.46	TNF-inducer
heat	Pattern_274	274	288	ENQWYLAAYQVTAGT	0.45	0.45	TNF non-inducer
heat	Pattern_275	275	289	NQWYLAAYQVTAGTG	0.45	0.45	TNF non-inducer
heat	Pattern_276	276	290	QWYLAAYQVTAGTGG	0.43	0.43	TNF non-inducer
heat	Pattern_277	277	291	WYLAAYQVTAGTGGW	0.43	0.43	TNF non-inducer
heat	Pattern_278	278	292	YLAAYQVTAGTGGWA	0.33	0.33	TNF non-inducer
heat	Pattern_279	279	293	LAAYQVTAGTGGWAA	0.35	0.35	TNF non-inducer
heat	Pattern_280	280	294	AAYQVTAGTGGWAAY	0.36	0.36	TNF non-inducer
heat	Pattern_281	281	295	AYQVTAGTGGWAAYT	0.34	0.34	TNF non-inducer
heat	Pattern_282	282	296	YQVTAGTGGWAAYTA	0.37	0.37	TNF non-inducer
heat	Pattern_283	283	297	QVTAGTGGWAAYTAN	0.37	0.37	TNF non-inducer
heat	Pattern_284	284	298	VTAGTGGWAAYTANT	0.39	0.39	TNF non-inducer

heat	Pattern_285	285	299	TAGTGGWAAYTANTG	0.43	0.43	TNF non-inducer
heat	Pattern_286	286	300	AGTGGWAAYTANTGG	0.41	0.41	TNF non-inducer
heat	Pattern_287	287	301	GTGGWAAYTANTGGN	0.38	0.38	TNF non-inducer
heat	Pattern_288	288	302	TGGWAAYTANTGGNR	0.39	0.39	TNF non-inducer
heat	Pattern_289	289	303	GGWAAYTANTGGNRY	0.36	0.36	TNF non-inducer
heat	Pattern_290	290	304	GWAAYTANTGGNRYA	0.33	0.33	TNF non-inducer
heat	Pattern_291	291	305	WAAYTANTGGNRYAA	0.33	0.33	TNF non-inducer
heat	Pattern_292	292	306	AAYTANTGGNRYAAY	0.33	0.33	TNF non-inducer
heat	Pattern_293	293	307	AYTANTGGNRYAAYK	0.33	0.33	TNF non-inducer
heat	Pattern_294	294	308	YTANTGGNRYAAYKS	0.35	0.35	TNF non-inducer
heat	Pattern_295	295	309	TANTGGNRYAAYKST	0.35	0.35	TNF non-inducer
heat	Pattern_296	296	310	ANTGGNRYAAYKSTR	0.36	0.36	TNF non-inducer
heat	Pattern_297	297	311	NTGGNRYAAYKSTRG	0.32	0.32	TNF non-inducer
heat	Pattern_298	298	312	TGGNRYAAYKSTRGN	0.34	0.34	TNF non-inducer
heat	Pattern_299	299	313	GGNRYAAYKSTRGNI	0.39	0.39	TNF non-inducer
heat	Pattern_300	300	314	GNRYAAYKSTRGNIV	0.41	0.41	TNF non-inducer
heat	Pattern_301	301	315	NRYAAYKSTRGNIVK	0.35	0.35	TNF non-inducer
heat	Pattern_302	302	316	RYAAYKSTRGNIVKA	0.35	0.35	TNF non-inducer
heat	Pattern_303	303	317	YAAYKSTRGNIVKAA	0.35	0.35	TNF non-inducer
heat	Pattern_304	304	318	AAYKSTRGNIVKAAY	0.38	0.38	TNF non-inducer
heat	Pattern_305	305	319	AYKSTRGNIVKAAYK	0.38	0.38	TNF non-inducer
heat	Pattern_306	306	320	YKSTRGNIVKAAYKT	0.36	0.36	TNF non-inducer
heat	Pattern_307	307	321	KSTRGNIVKAAYKTT	0.32	0.32	TNF non-inducer
heat	Pattern_308	308	322	STRGNIVKAAYKTTR	0.27	0.27	TNF non-inducer
heat	Pattern_309	309	323	TRGNIVKAAYKTTRG	0.3	0.3	TNF non-inducer
heat	Pattern_310	310	324	RGNIVKAAYKTTRGT	0.29	0.29	TNF non-inducer
heat	Pattern_311	311	325	GNIVKAAYKTTRGTV	0.27	0.27	TNF non-inducer
heat	Pattern_312	312	326	NIVKAAYKTTRGTVG	0.3	0.3	TNF non-inducer
heat	Pattern_313	313	327	IVKAAYKTTRGTVGL	0.29	0.29	TNF non-inducer
heat	Pattern_314	314	328	VKAAYKTTRGTVGLA	0.32	0.32	TNF non-inducer
heat	Pattern_315	315	329	KAAYKTTRGTVGLAA	0.35	0.35	TNF non-inducer
heat	Pattern_316	316	330	AAYKTTRGTVGLAAY	0.34	0.34	TNF non-inducer
heat	Pattern_317	317	331	AYKTTRGTVGLAAYS	0.3	0.3	TNF non-inducer
heat	Pattern_318	318	332	YKTTRGTVGLAAYSS	0.29	0.29	TNF non-inducer
heat	Pattern_319	319	333	KTTRGTVGLAAYSSF	0.32	0.32	TNF non-inducer
heat	Pattern_320	320	334	TTRGTVGLAAYSSFK	0.41	0.41	TNF non-inducer
heat	Pattern_321	321	335	TRGTVGLAAYSSFKI	0.42	0.42	TNF non-inducer
heat	Pattern_322	322	336	RGTVGLAAYSSFKIT	0.42	0.42	TNF non-inducer
heat	Pattern_323	323	337	GTVGLAAYSSFKITF	0.41	0.41	TNF non-inducer
heat	Pattern_324	324	338	TVGLAAYSSFKITFF	0.42	0.42	TNF non-inducer
heat	Pattern_325	325	339	VGLAAYSSFKITFFF	0.45	0.45	TNF non-inducer

heat	Pattern_326	326	340	GLAAYSSFKITFFFA	0.42	0.42	TNF non-inducer
heat	Pattern_327	327	341	LAAYSSFKITFFFAA	0.44	0.44	TNF non-inducer
heat	Pattern_328	328	342	AAYSSFKITFFFAAY	0.44	0.44	TNF non-inducer
heat	Pattern_329	329	343	AYSSFKITFFFAAYP	0.44	0.44	TNF non-inducer
heat	Pattern_330	330	344	YSSFKITFFFAAYPY	0.45	0.45	TNF non-inducer
heat	Pattern_331	331	345	SSFKITFFFAAYPYI	0.52	0.52	TNF-inducer
heat	Pattern_332	332	346	SFKITFFFAAYPYIF	0.54	0.54	TNF-inducer
heat	Pattern_333	333	347	FKITFFFAAYPYIFI	0.56	0.56	TNF-inducer
heat	Pattern_334	334	348	KITFFFAAYPYIFIA	0.46	0.46	TNF-inducer
heat	Pattern_335	335	349	ITFFFAAYPYIFIAT	0.49	0.49	TNF-inducer
heat	Pattern_336	336	350	TFFFAAYPYIFIATF	0.46	0.46	TNF-inducer
heat	Pattern_337	337	351	FFFAAYPYIFIATFF	0.47	0.47	TNF-inducer
heat	Pattern_338	338	352	FFAAYPYIFIATFFA	0.49	0.49	TNF-inducer
heat	Pattern_339	339	353	FAAYPYIFIATFFAA	0.49	0.49	TNF-inducer
heat	Pattern_340	340	354	AAYPYIFIATFFAAY	0.48	0.48	TNF-inducer
heat	Pattern_341	341	355	AYPYIFIATFFAAYF	0.47	0.47	TNF-inducer
heat	Pattern_342	342	356	YPYIFIATFFAAYFL	0.47	0.47	TNF-inducer
heat	Pattern_343	343	357	PYIFIATFFAAYFLL	0.44	0.44	TNF non-inducer
heat	Pattern_344	344	358	YIFIATFFAAYFLLP	0.45	0.45	TNF non-inducer
heat	Pattern_345	345	359	IFIATFFAAYFLLPY	0.49	0.49	TNF-inducer
heat	Pattern_346	346	360	FIATFFAAYFLLPYI	0.47	0.47	TNF-inducer
heat	Pattern_347	347	361	IATFFAAYFLLPYIF	0.44	0.44	TNF non-inducer
heat	Pattern_348	348	362	ATFFAAYFLLPYIFI	0.47	0.47	TNF-inducer
heat	Pattern_349	349	363	TFFAAYFLLPYIFIA	0.45	0.45	TNF non-inducer
heat	Pattern_350	350	364	FFAAYFLLPYIFIAA	0.52	0.52	TNF-inducer
heat	Pattern_351	351	365	FAAYFLLPYIFIAAA	0.54	0.54	TNF-inducer
heat	Pattern_352	352	366	AAYFLLPYIFIAAAY	0.53	0.53	TNF-inducer
heat	Pattern_353	353	367	AYFLLPYIFIAAAYG	0.54	0.54	TNF-inducer
heat	Pattern_354	354	368	YFLLPYIFIAAAYGQ	0.53	0.53	TNF-inducer
heat	Pattern_355	355	369	FLLPYIFIAAAYGQI	0.51	0.51	TNF-inducer
heat	Pattern_356	356	370	LLPYIFIAAAYGQIG	0.57	0.57	TNF-inducer
heat	Pattern_357	357	371	LPYIFIAAAYGQIGV	0.62	0.62	TNF-inducer
heat	Pattern_358	358	372	PYIFIAAAYGQIGVD	0.6	0.6	TNF-inducer
heat	Pattern_359	359	373	YIFIAAAYGQIGVDF	0.46	0.46	TNF-inducer
heat	Pattern_360	360	374	IFIAAAYGQIGVDFY	0.38	0.38	TNF non-inducer
heat	Pattern_361	361	375	FIAAAYGQIGVDFYA	0.33	0.33	TNF non-inducer
heat	Pattern_362	362	376	IAAAYGQIGVDFYAA	0.33	0.33	TNF non-inducer
heat	Pattern_363	363	377	AAAYGQIGVDFYAAA	0.33	0.33	TNF non-inducer
heat	Pattern_364	364	378	AAYGQIGVDFYAAAY	0.34	0.34	TNF non-inducer
heat	Pattern_365	365	379	AYGQIGVDFYAAAYN	0.33	0.33	TNF non-inducer
heat	Pattern_366	366	380	YGQIGVDFYAAAYNE	0.3	0.3	TNF non-inducer

heat	Pattern_367	367	381	GQIGVDFYAAAYNEC	0.28	0.28	TNF non-inducer
heat	Pattern_368	368	382	QIGVDFYAAAYNECG	0.28	0.28	TNF non-inducer
heat	Pattern_369	369	383	IGVDFYAAAYNECGE	0.28	0.28	TNF non-inducer
heat	Pattern_370	370	384	GVDFYAAAYNECGEV	0.25	0.25	TNF non-inducer
heat	Pattern_371	371	385	VDFYAAAYNECGEVN	0.22	0.22	TNF non-inducer
heat	Pattern_372	372	386	DFYAAAYNECGEVNP	0.25	0.25	TNF non-inducer
heat	Pattern_373	373	387	FYAAAYNECGEVNPI	0.33	0.33	TNF non-inducer
heat	Pattern_374	374	388	YAAAYNECGEVNPIA	0.38	0.38	TNF non-inducer
heat	Pattern_375	375	389	AAAYNECGEVNPIAA	0.41	0.41	TNF non-inducer
heat	Pattern_376	376	390	AAYNECGEVNPIAAY	0.4	0.4	TNF non-inducer
heat	Pattern_377	377	391	AYNECGEVNPIAAYG	0.38	0.38	TNF non-inducer
heat	Pattern_378	378	392	YNECGEVNPIAAYGT	0.34	0.34	TNF non-inducer
heat	Pattern_379	379	393	NECGEVNPIAAYGTV	0.36	0.36	TNF non-inducer
heat	Pattern_380	380	394	ECGEVNPIAAYGTVI	0.35	0.35	TNF non-inducer
heat	Pattern_381	381	395	CGEVNPIAAYGTVIN	0.45	0.45	TNF non-inducer
heat	Pattern_382	382	396	GEVNPIAAYGTVINV	0.5	0.5	TNF-inducer
heat	Pattern_383	383	397	EVNPIAAYGTVINVH	0.47	0.47	TNF-inducer
heat	Pattern_384	384	398	VNPIAAYGTVINVHL	0.5	0.5	TNF-inducer
heat	Pattern_385	385	399	NPIAAYGTVINVHLK	0.49	0.49	TNF-inducer
heat	Pattern_386	386	400	PIAAYGTVINVHLKA	0.49	0.49	TNF-inducer
heat	Pattern_387	387	401	IAAYGTVINVHLKAA	0.51	0.51	TNF-inducer
heat	Pattern_388	388	402	AAYGTVINVHLKAAY	0.52	0.52	TNF-inducer
heat	Pattern_389	389	403	AYGTVINVHLKAAYT	0.5	0.5	TNF-inducer
heat	Pattern_390	390	404	YGTVINVHLKAAYTG	0.44	0.44	TNF non-inducer
heat	Pattern_391	391	405	GTVINVHLKAAYTGQ	0.41	0.41	TNF non-inducer
heat	Pattern_392	392	406	TVINVHLKAAYTGQF	0.38	0.38	TNF non-inducer
heat	Pattern_393	393	407	VINVHLKAAYTGQFH	0.43	0.43	TNF non-inducer
heat	Pattern_394	394	408	INVHLKAAYTGQFHI	0.45	0.45	TNF non-inducer
heat	Pattern_395	395	409	NVHLKAAYTGQFHIA	0.39	0.39	TNF non-inducer
heat	Pattern_396	396	410	VHLKAAYTGQFHIAP	0.35	0.35	TNF non-inducer
heat	Pattern_397	397	411	HLKAAYTGQFHIAPY	0.35	0.35	TNF non-inducer
